# Enhancing activity and selectivity of palladium catalysts in ketone α-arylation by tailoring the imine chelate of pyridinium amidate (PYA) ligands†

**DOI:** 10.1039/d4cy01337a

**Published:** 2024-12-18

**Authors:** Esaïe Reusser, Michael Aeschlimann, Martin Albrecht

**Affiliations:** a Department of Chemistry, Biochemistry and Pharmaceutical Sciences, University of Bern Freiestrasse 3 3012 Bern Switzerland martin.albrecht@unibe.ch

## Abstract

Even though α-arylation of ketones is attractive for direct C–H functionalization of organic substrates, the method largely relies on phosphine-ligated palladium complexes. Only recently, efforts have focused on developing nitrogen-based ligands as a more sustainable alternative to phosphines, with pyridine-functionalized pyridinium amidate (pyr-PYA) *N*,*N*′-bidentate ligands displaying good selectivity and activity. Here, we report on a second generation set of catalyst precursors that feature a 5-membered N-heterocycle instead of a pyridine as chelating unit of the PYA ligand to provide less steric congestion for the rate-limiting transmetalation of the enolate. To this end, new heterocycle-functionalized PYA palladium(ii) complexes containing an oxazole (5b), *N*-phenyl-triazole (5c), *N*-methyl pyrazole (5d), *N*-phenyl-pyrazole, (5e), *N*-xylyl-pyrazole (5f), and *N*-isopropyl-pyrazole (5g) were synthesized compared to the parent pyr-PYA complex 5a. Less packing of the palladium coordination sphere was evidenced from solid state X-ray diffraction analysis. While the catalytic activity of the oxazole system was lower, all other complexes showed higher activity. In particular, complex 5g comprised of an electron-donating and sterically demanding iPr-pyrazole chelating PYA ligand is remarkably stable towards air and moisture and shows outstanding catalytic activity with complete selectivity (>99% yield) and turnover frequencies up to 1200 h^−1^, surpassing that of parent 5a by one order of magnitude and rivalling the most active phosphine-based palladium systems. Kinetic studies demonstrate a first order rate-dependence on palladium and the substrate. Some deviation of linearity together with poisoning experiments suggest a mixed homogeneous/heterogeneous pathway, though the reproducible kinetics of *in situ* catalyst recycling experiments strongly point to a molecularly defined active species, demonstrating the high potential of PYA-based ligands.

## Abstract 2

Palladium complexes with chelating pyridinium amidate ligands are efficient catalysts for the α-arylation of ketones. Their catalytic activity depends on the chelating group at the PYA, with iPr-substituted pyrazole PYAs reaching high turnover frequencies.

## Graphical abstract

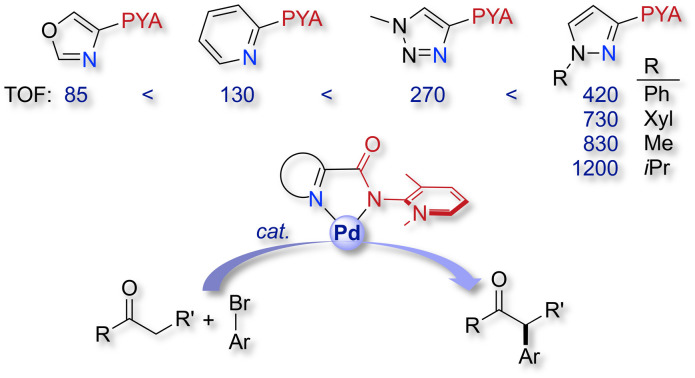

## Introduction

**Fig. 1:**
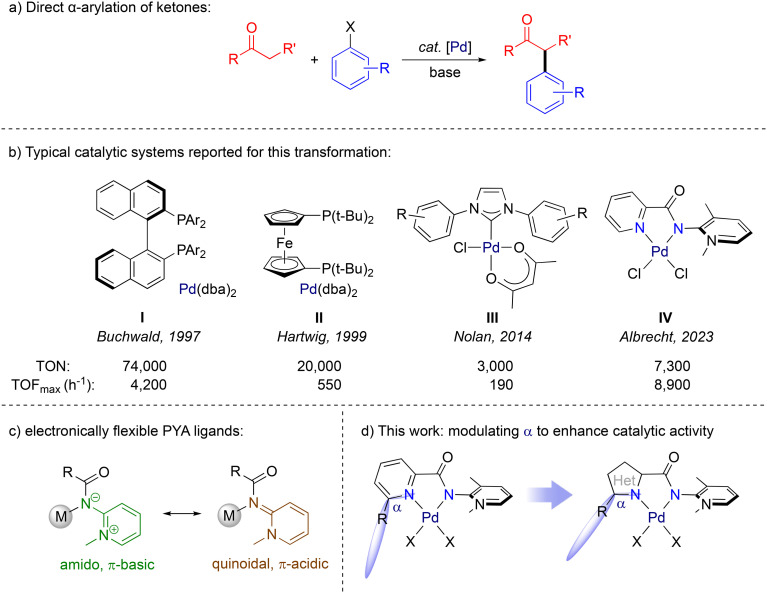
a) General reaction scheme of ketone α-arylation; b) previously reported catalytic systems for the ketone α-arylation reaction and their optimized performance; c) limiting resonance structures of *ortho*-pyridinium amidate (PYA) ligand; d) structural modification of the precatalyst targeted in this work and depiction of the *α*-angle.

Transition-metal-catalyzed cross-coupling methodologies have become a fundamental strategy in the synthesis of pharmaceuticals, natural products, and fine chemicals.^1–3^ In the late 1990s, Buchwald and Hartwig broadened the scope of cross-coupling reactions to include the direct α-arylation of enolizable ketones (Fig. 1a).^4–7^ This expansion offers access to versatile chemical intermediates and to a structural motif that is abundant in many active pharmaceutical ingredients.^8–11^ The previously used stoichiometric methods often suffered from narrow scope and selectivity, whereas transition metal catalysts overcome these problems and additionally make the process considerably more sustainable by reducing the amount of (hazardous) waste.^12,13^ Early work demonstrated the relevance of palladium for the catalytic α-arylation of ketones, especially when employed in conjunction with sterically hindered, strongly electron-donating phosphine ligands.^14–16^ Specifically, wide bite angle diphosphine ligands such as **I** or **II** (Fig. 1b) impart high steric hindrance and therefore disfavor the β-H elimination side reaction.^4,5,17^ However, phosphine-based catalysts often suffer from low thermal stability and high sensitivity towards oxidation. Furthermore, due to their highly apolar nature, these ligands are often challenging to separate from the reaction mixture. As alternative ligands to phosphines, N-heterocyclic carbenes (NHCs) have emerged as valuable ligands to palladium for α-arylation catalysis and have shown remarkable potency in activating aryl chloride (complex **III**).^18–21^ They also offer access to molecularly well-defined pre-catalysts, in contrast to first-generation catalytic systems that were generated *in situ*.^22–26^ Notably, though, catalysts based on NHCs generally exhibit moderate turnover frequencies (TOFs) compared to chelating phosphine systems.^21^ Furthermore, earlier investigations on carbene-based ligands in alkene oligomerization showed that those ligands tend to reductively eliminate from the hydride intermediate *via* β-hydrogen elimination, yielding the corresponding imidazolium salt and ensuing deactivation, although such processes might be prevented under basic conditions.^27–29^

Similar to NHCs, pyridinium amidates (PYAs) are characterized by strong σ-donation that is beneficial in stabilizing metal complexes under harsh conditions. Indeed, initial investigations suggested that the donor strength of these ligands is comparable to that of traditional NHC ligands.^30–32^ Furthermore, a typical feature of PYA ligands is their ability to adopt different resonance structures that exhibit either π-basic or π-acidic properties, implying electronic donor flexibility that responds to the electronic environment of the coordinated metal center (Fig. 1c).^33^ This adaptability may be particularly beneficial in palladium-catalyzed cross-coupling processes involving both oxidative addition and reductive elimination steps. Ligands capable of flexibly adjusting their donor properties are expected to enhance the accessibility of both zero-valent and +II oxidation states, thus contributing to the efficiency of the catalytic system. The beneficial role of PYA ligands has been demonstrated in previous studies on palladium-catalyzed cross-coupling reactions, such as Suzuki–Miyaura reactions.^34^ These PYA ligands are readily synthesized from cheap aminopyridine, and offer vast opportunities for introducing potentially chelating donor sites.^33,35^ We recently exploited the facile accessibility by introducing a set of air- and moisture-stable palladium(ii) complexes featuring a *N*,*N*′-bidentate chelating ligand based on electronically flexible PYA ligands linked to pyridine (**IV**, Fig. 1b)^36^ and showed their activity in catalytic α-arylation of ketones.^37^ Their performance is remarkably high for nitrogen-based ligands, with turnover numbers reaching up to 7300 and turnover frequencies approaching 9000 h^−1^, placing these catalysts on a competitive level with the best NHC and phosphine-based systems. Mechanistic investigations indicated that both oxidative addition and reductive elimination proceed swiftly in PYA-based catalysts, likely facilitated by the donor flexibility of the PYA ligand, while enolate coordination was identified as the turnover-limiting step. The previously observed ligand effects strongly support a catalytically active species that maintains coordination with the pyridyl–PYA ligand.^37^ These observations suggest that further customization of the imine donor, such as replacing the pyridyl unit by other donor units holds promise for enhancing catalytic performance. Specifically, the introduction of 5-membered heterocyclic ligand scaffolds affects the space available for coordination at the *cis*-position by alleviating the steric impact of substituents, therefore strongly modulating activity and selectivity of the catalyst. The more open *α* angle (Fig. 1d) might suppress the deleterious effect of bulky ligand substituents on the catalyst selectivity while retaining the high activity.

Here we demonstrate that palladium(ii) complexes with a chelating PYA ligand comprised of a pyrazole or triazole donor constitute precursors to highly active and selective ketone α-arylation catalysts, enabling full conversions in less than 10 min. This work illustrates the usefulness of ligand design to tailor selectivity and activity of existing catalysts.

## Results and discussion

### Synthesis and characterization of PYA palladium complexes

**Fig. 2:**
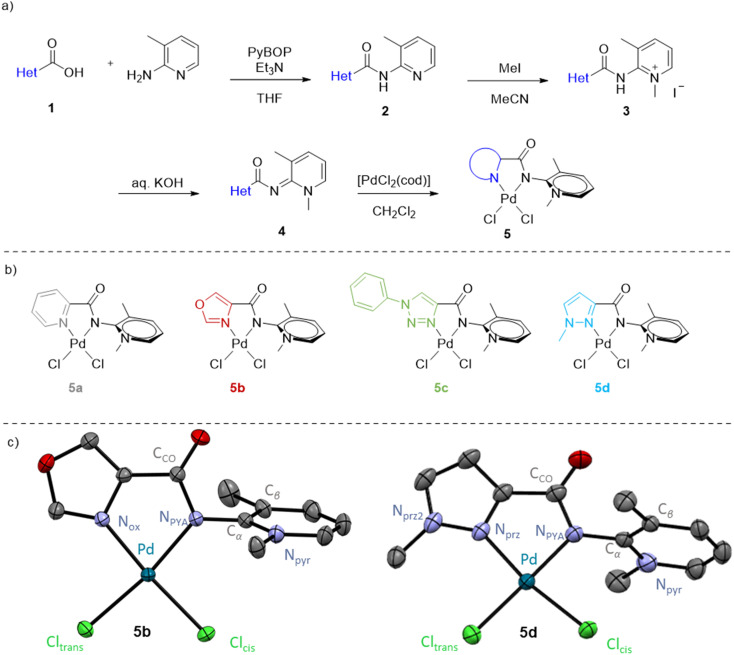
(a) General synthetic scheme for the preparation of the Pd-PYA complexes 5a to 5d; (b) schematic drawings of complexes 5a–d; (c) molecular structures of complexes 5b and 5d determined by X-ray diffraction analysis (30% probability ellipsoids, hydrogen atoms and co-crystallized solvent molecules omitted for clarity).

A series of novel PYA palladium complexes were prepared starting from commercially available 2-amino-3-methylpyridine in four straightforward synthetic steps (Fig. 2a). In the presence of benzotriazol-1-yloxytripyrrolidinophosphonium hexafluorophosphate (PyBOP) as coupling agent and NEt_3_, the aminopyridine reacted with the appropriate heterocyclic carboxylic acid 1a–1d to afford the corresponding amides 2a–2d in good yields (62–79%). The reaction was followed by ^1^H NMR spectroscopy, since the appearance of the NH resonance at *δ*_H_ ∼10 ppm together with the disappearance of the broad singlet at *δ*_H_ = 5.8 ppm due to the aniline NH_2_ group were diagnostic for the formation of the corresponding amides 2. Subsequent methylation of the pyridine was selective even in the presence of an excess of methyl iodide, indicated by the appearance of a new *N*-methyl resonance around 4.2 ppm in the ^1^H NMR spectrum together with a marked downfield shift of the aromatic pyridine signals. Upon precipitation with Et_2_O, iodide salts 3a–3d were isolated in 60–78% yield. Deprotonation of these pyridinium salts proceeded smoothly in the presence of an excess aqueous KOH and yielded the neutral PYA ligands 4a–4d in excellent yields (79–94%), indicated by the diagnostic upfield shift of the pyridyl *N*-methyl resonance from 4.3 to 3.8 ppm together with the disappearance of the NH signal. Coordination of palladium using [PdCl_2_(cod)] was essentially quantitative and afforded complexes 5a–5d as yellow solids (Fig. 2b). Upon palladium coordination, the PYA N–CH_3_ signal shifted back to ∼4.3 ppm suggesting a similar electronic influence of a proton on the PYA unit as the palladium(ii) fragment. All the palladium complexes were stable towards air, light and moisture for weeks. Crystals suitable for X-ray diffraction were grown for complexes 5b and 5d by slow diffusion of Et_2_O into a complex solution in CH_2_Cl_2_ and MeOH, respectively (Fig. 2c).

Both complexes displayed a palladium center in a slightly distorted square planar geometry (*τ*_4_ = 0.094 and 0.034 for 5b and 5d, respectively)^38^ similar to the configuration of 5a (Table 1). For all complexes Pd–Cl_*trans*_ was slightly longer compared to Pd–Cl_*cis*_, indicating a stronger *trans* influence of the PYA donor site compared with the heterocyclic imine donor. Ligand bite angles, which are known to be highly relevant in cross-coupling processes,^39^ are similar for 5a, 5b and 5d (N_het_–Pd–N_PYA_ 79.8 ± 0.6°), despite the transition from a 6- to a 5-membered heterocyclic scaffold. Analysis of the buried volume (%*V*_bur_)^40^ induced by the different ligands revealed that switching from 6- to 5-membered heterocyclic coordination sites effectively reduces the %*V*_bur_ (38.6% for 5b*vs.* 40.2% for 5a). The steric hindrance of the pyridine ligand was restored upon adding a methyl substituent at the pseudo-*ortho* position of the heterocycle, despite its distal orientation from the Pd center (%*V*_bur_ = 40.6 for 5d). The wider *α* angle in 5b and 5d (128.8(13)° and 123.3(15)° *vs.* 119.1(1)° in 5a) illustrates the larger space created by the 5- *vs.* 6-membered heterocycle. Consequently, the distance between the (pseudo-)*ortho* proton of the heterocycle and the adjacent chloride Cl_*trans*_ is some 2.69 Å for 5a, yet markedly longer at 3.08 Å in 5b.

**Table 1:** Selected crystallographic metrics for complex 5a, 5b and 5d

	5a^a^ (Het = py)	5b (Het = ox)	5d (Het = prz)
Pd–N_Het_/Å	2.013(3)	1.9979(15)	2.0369(14)
Pd–N_PYA_/Å	2.006(3)	2.0479(15)	2.0253(15)
Pd–Cl_*trans*_/Å	2.3030(9)	2.3086(5)	2.2990(5)
Pd–Cl_*cis*_/Å	2.2935(11)	2.2977(5)	2.2861(5)
N_PYA_–C_α_/Å	1.395(4)	1.392(2)	1.392(2)
N_PYA_–C_CO_/Å	1.340(4)	1.356(2)	1.349(2)
N_PYA_–Pd–N_Het_ /°	80.35(10)	80.33(6)	79.20(6)
%*V*_bur_^b^	40.2	38.6	40.6
*α*/°^c^	119.1(3)	128.8(13)	123.3(15)
*τ*_4_ d	0.084	0.094	0.034

a Data from ref. 37.

b Percentage buried volume %*V*_bur_ determined according to ref. 40, see ESI† for details

c The *α* angle was determined as N_py_–C_py_–H for 5a, N_ox_–C_ox_–H for 5b, and N_prz_–N_prz2_–C_Me_ for 5d (see also Fig. 1d).

d Tetrahedral distortion parameter *τ*_4_ determined according to ref. 38.

### Evaluation in the catalytic α-arylation of propiophenone

The catalytic activity of complexes 5b–5d was probed in the α-arylation of ketones using propiophenone and bromobenzene as model substrates (Table 2).^37^ Comparison to complex 5a as benchmark catalyst precursor revealed a substantial influence of the heterocyclic ligand on the catalytic activity. Substitution of pyridine with the oxazole moiety (5b) had a negative effect on the reaction rate, reducing the TOF_max_ from 130 to 85 h^−1^ as well as a reduced yield at full conversion of ArBr from 87% to 70% (entries 1,2). In contrast, insertion of either a triazole or a pyrazole unit (5c and 5d) improved the activity, affording TOF_max_ = 270 and 830 h^−1^, respectively (entries 3, 4). However, triazole complex 5c suffered from a diminished selectivity as it only reached a moderate yield of 77% at full conversion, while the pyrazole-substituted analogue 5d gave 91% yield after 1 h. The higher activity of *N*-methyl pyrazolyl-functionalized complex 5d might arise from the stronger donor properties of pyrazole compared to the oxazole and triazole analogues. Indeed, the lower p*K*_a_ of the parent *N*-methyl pyrazole heterocycle (p*K*_a_ 2.06) suggests a higher Lewis basicity than that of oxazole (p*K*_a_ 0.8) or triazole (p*K*_a_ 1.25).^41–44^ Steric arguments may also play a role, especially due to the methyl substituent on the pyrazole unit, though the %*V*_bur_ of 5d is similar to 5a (see above).

**Table 2:** Activity of complexes 5a–5d in propiophenone α-arylation^a^

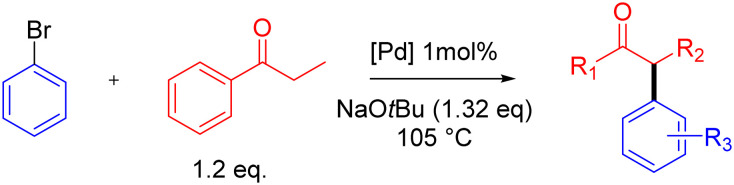					
Entry	[Pd]	Optimized yield^b^ (time)	Yield	TOF_max_/h^−1^	*k*_ini_/h^−1^
1	5a	87% (2 h)	82%	130	1.7
2	5b	70% (2 h)	67%	85	1.0
3	5c	77% (1 h)	77%	270	3.3
4	5d	91% (1 h)	84%	830	8.3

a Reaction conditions: PhBr (1.0 mmol), propiophenone (1.2 mmol), [Pd] (0.01 mmol), NaO*t*Bu (1.32 mmol), 1,4-dioxane (2.0 mL), 105 °C under N_2_, yield determined by GC analysis using hexamethylbenzene as internal standard; for TOF_max_ evaluations see Fig. S63;† for calculation of initial rate constants *k*_ini_ see Fig. S64;†

b Optimized yields determined without sampling, using identical conditions except propiophenone (1.0 mmol) NaO*t*Bu (1.1 mmol), dioxane (1.0 mL).

### Kinetic and mechanistic investigations

**Fig. 3:**
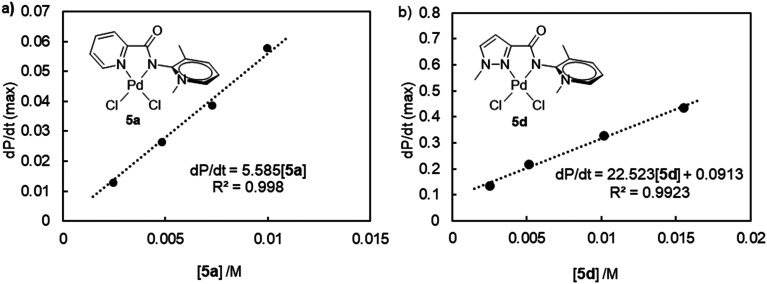
Rate-dependence with respect to catalyst concentration a) for complex 5a, and b) for complex 5d, indicating a first-order dependence for both catalytic systems.

Due to its enhanced activity, complex 5d containing a pyrazole donor was investigated in more detail as a catalyst. Running the ketone arylation at different reaction temperatures allowed for an Eyring analysis of the initial reaction rates (Fig. S79†) and furnished activation parameters Δ*H*^‡^ = 109 ± 3 kJ mol^−1^ and Δ*S*^‡^ = −34 ± 8 J K^−1^ mol^−1^. These values are very similar to those established for 5a (Δ*H*^‡^ = 105 ± 10 kJ mol^−1^ and Δ*S*^‡^ = −89 ± 26 J K^−1^ mol^−1^),^37^ suggesting a similar catalytic cycle and corroborating an associative turnover-limiting step.^45^ Variation of the catalyst concentration indicated a first-order rate-dependence in catalyst, both for 5a and 5d (Fig. 3, S67–S69†).^46^ These observations demonstrate the involvement of the catalyst in the turnover-limiting step and strongly point towards a molecularly defined catalytically active species. Notably, the linear fit for 5d deviates significantly from the expected zero *y*-intercept, pointing to some curvature and a partial order in catalyst at higher concentrations. Such a behavior may be caused by an off-cycle monomer-dimer equilibrium, or by the involvement of nanoparticles, with ligand dissociation as a fast pre-equilibrium. In agreement with a more intricate role of the catalyst, log–log plots revealed a broken order in [5d], yet clear first order in [5a] (Fig. S74†).

**Fig. 4:**
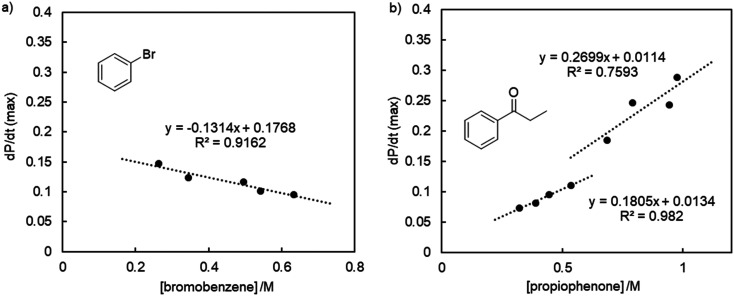
Rate-dependence of the 5d-catalyzed α-arylation on a) bromobenzene, and b) propiophenone, revealing essentially zero-order dependence on the aryl halide, and different regimes of first-order dependence on the ketone.

Modulating the concentration of bromobenzene suggests a pseudo zero order dependence of the rate (Fig. 4a, S76†), corroborating previous conclusions that oxidative addition is not rate-limiting with these PYA palladium catalysts.^37,47^ The slightly negative slope indicates an inhibitory effect, which may be linked to the formation of radical species or the involvement of nanoparticles.^48–50^

In contrast, variation of the propiophenone concentration gave a less clear picture. At low concentration (<0.65 M), a linear correlation and first-order reaction kinetics were established (Fig. 4, S75†), corroborating the involvement of the ketone in the rate-limiting step as previously deduced from Hammett studies.^37,51^ However, at higher propiophenone concentrations (>0.65 M), the correlation is less obvious and the slope different. This deviation might be attributed to the changed conditions, as higher ketone concentrations require larger amounts of NaO*t*Bu. Alternatively, the data may be correlated to a sigmoidal curve, which would point to a departure from a homogeneous model with a molecular catalyst towards the formation of heterogeneous aggregates.

In an attempt to distinguish a homogeneous from a heterogeneous mode of action, poisoning experiments were performed. To this end, catalytic runs were carried out under standard conditions,^52^ yet at approximately 30% conversion, either PPh_3_ (10 eq. relative to Pd) or Hg (>70 eq. relative to Pd) was added to the reaction mixture.^53^ The pertinent conversion profiles indicate that either of the two additives effectively inhibit the catalytic activity of both 5a and 5d (Fig. S77 and S78†). This outcome seems controversial at first sight as an excess of phosphine is supposed to competitively bind to the metal center and thus inhibit substrate coordination in a homogeneous system, whereas mercury is assumed to act on a heterogeneous catalyst through the formation of amalgams and ensuing passivation of reactive surfaces.^45^ Both poisoning tests have their limitations,^54,55^ and furthermore, we noted that the activity was not immediately suppressed, but only some 30 min after addition of the additive for both PPh_3_ and Hg, suggesting some residual activity even after poisoning. These observations therefore suggest mixed homo- and heterogeneous mechanisms, in which colloidal—possibly ligand-stabilized^56^—palladium(0) constitutes a resting state, from which PYA–palladium species can dissociate and operate as homogeneous catalysts, a mode of action that has sometimes been referred to as pseudo-homogeneous mechanism,^57–60^ It also hints to higher complexity than the simplistic oxidative addition–transmetalation–reductive elimination cycle generally portrayed in textbooks.^61^

**Fig. 5:**
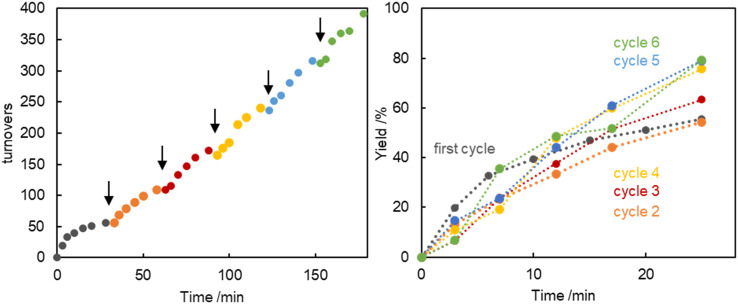
Catalyst recycling experiment for the arylation of propiophenone with complex 5d. Left: turnover increase upon addition of new batches of substrates every 30 min (each addition point marked with an arrow); right: superimposed time-yield profiles of the different catalyst recycling runs indicating no substantial change in reaction kinetics. Initial reaction conditions: PhBr (1.0 mmol), propiophenone (1.0 mmol), 5d (0.01 mmol), NaO*t*Bu (1.1 mmol), 1,4-dioxane (2.0 mL), 105 °C under N_2_. At each addition point, *i.e.* every 30 min, a pre-heated solution of PhBr (1.0 mmol) and NaO*t*Bu (1.1 mmol) in 1,4-dioxane (1.0 mL) followed by propiophenone (1.0 mmol) was added. Yields determined by GC analysis using hexamethylbenzene as internal standard.

The robustness of the catalytically active species derived from 5d was further probed by a set of *in situ* catalyst recycling experiments. When adding a new batch of substrates to the reaction mixture every 30 min, no significant loss of activity nor any modification of the conversion profile was noted for up to five additional cycles (Fig. 5). These runs indicate a persistently active species. This conclusion was further reinforced by a filtration experiment, in which the reaction mixture was filtered through a 200 nm membrane after 30 min and charged with a new batch of substrates. Conversion of this new batch was essentially identical to the conversion before filtration (Fig. S80†), suggesting either small colloids or molecular palladium species as catalyst resting state. Notably, when the filtration was performed under air rather than a N_2_ atmosphere, the catalytic activity markedly decreased (Fig. S80†), which points to a palladium(0) resting state and thus corroborates the catalyst poisoning data.

### Catalyst activation pathways

While the reduction of Pd^II^ catalyst precursors in the presence of phosphine ligands has been extensively studied,^62–66^ much less is known about phosphine-free reduction pathways.^67–70^ We therefore investigated two specific scenarios for palladium(0) generation from complex 5d, namely i) enolate coordination to palladium(ii) *via* a ligand substitution process followed by reductive C–Cl bond elimination, and ii) β hydrogen elimination from the coordinated enolate with formation of a meta-stable palladium hydride intermediate (Fig. S81†). In a series of stoichiometric experiments, addition of either propiophenone or NaO*t*Bu immediately afforded a new species according to the downfield shift of the PYA resonances in the ^1^H NMR spectrum, which was attributed to coordination of the additive (Fig. S82†).^71^ When using 4-fluoropropiophenone, the new species was very similar to the one formed upon propiophenone addition, with slight ^1^H NMR shift differences attributed to the electronic effect of the fluorine substituent. For instance, the PYA *N*-methyl signal moved from 3.48 ppm in 5d to 3.66 and 3.64 upon addition of propiophenone and 4-fluoropropiophenone, respectively.^72^ These solutions were stable also when heated to 80 °C. However, when mixing 5d with stoichiometric amounts of the ketone and NaO*t*Bu, an immediate reaction occurred, resulting in the formation of a species characterized by broad signals in the ^1^H NMR spectrum, along with the appearance of a precipitate. MS analysis did not show any chlorinated propiophenone, thus discarding a C–Cl reductive elimination process as the predominant catalyst activation step. Remarkably, when using 4-fluoropropriophenone as the ketone together with the base and complex 5d, all ketone was consumed within 10 min according to ^1^H NMR spectroscopy, and the ^19^F NMR spectrum did not reveal any signal, suggesting either precipitation of the product or massive broadening of the signal (Fig. S83 and S84†). This loss of ^19^F NMR signal and the broadened ^1^H NMR resonances might concur with β-hydrogen elimination as catalyst activation pathway, if the formed unsubstituted enone is unstable.

### Pyrazole functionalization for catalyst optimization

**Fig. 6:**
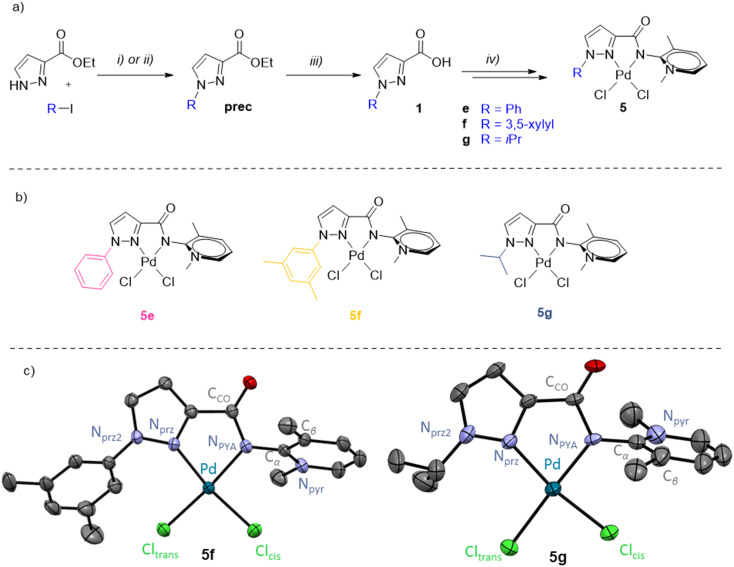
a) Synthetic scheme for the preparation of the carboxylic acids 1e–1g and complexes 5e–5g. Reactions and conditions: i) DMEDA (0.2 eq.), CuI 0.1 eq., K_2_CO_3_ 1.2 eq., 1,4-dioxane; ii) Cs_2_CO_3_ 2 eq., MeCN; iii) first LiOH 2.0 eq. MeOH/H_2_O, then HCl_aq_. 2.2 eq.; iv) according to Fig. 2a. **Prec-g** and 1g were not isolated and directly used for the next step; b) schematic drawing of pyrazole-functionalized PYA palladium complexes 5e–5g; c) molecular structures of complexes 5f and 5g determined by X-ray diffraction analysis (30% probability ellipsoids, hydrogen atoms and co-crystallized solvent molecules omitted for clarity).

The mechanistic experiments strongly suggest a molecularly defined active species, also supported by conversion rates that are strongly influenced by the ligand design. For this reason, the best-performing ligand 4d was further modified to improve the catalytic activity. Variation of the pyrazole wingtip substituent from Me in 5d to phenyl (5e), 3,5-dimethylphenyl (5f), and iso-propyl (5g) aimed at probing both the electronic and steric implications of this substituent on the catalytic performance. The corresponding ligand precursors were prepared starting from commercially available ethyl 1*H*-pyrazole-3-carboxylate by nucleophilic substitution (R = alkyl) or copper-catalyzed Ullmann coupling (R = aryl),^73^ and subsequent ester hydrolysis to yield the carboxylic acids 1e–1g (Fig. 6a). Subsequent coupling with aminopyridine followed by pyridine methylation and palladation was performed according to methodologies used for 5d and afforded complexes 5e–5g (Fig. 6b).

While the NMR spectra of 5e and 5f are unremarkable, ^1^H NMR spectroscopy of 5g showed a marked downfield shift of the isopropyl C*H* proton upon complexation from *δ*_H_ = 4.53 in ligand 4g to 5.96 ppm in the complex. Such strong deshielding is indicative of an electrostatic interaction with the chloride ligand.^33,74^ Crystals suitable for X-ray diffraction of complexes 5f and 5g were obtained by slow diffusion of Et_2_O into a CH_2_Cl_2_ solution of the complexes (Fig. 6c). Similarly to complexes 5a and 5d, the molecular structures of 5f and 5g feature a square planar palladium center with the Pd–Cl_*trans*_ bond consistently longer than Pd–Cl_*cis*_ due to the higher *trans* influence of the PYA donor (Table 3). The steric impact of the bidentate PYA ligand in complexes 5f and 5g is moderately higher than in the pyridyl–PYA system of 5a (%*V*_bur_ = 42.3% *vs.* 40.2%). For comparison, the introduction of an aryl-substituent at the pyridine 6-position increases the %*V*_bur_ by >8%.^37^ The structure of 5g features a close contact between the iPr CH hydrogen and Cl_*trans*_ (H⋯Cl 2.525(3) Å), in agreement with the marked downfield shift of this resonance in solution NMR spectroscopy.

**Table 3:** Selected crystallographic metrics for complexes 5f and 5g

	5f	5g
Pd–N_prz_/Å	2.0375(17)	2.033(11)
Pd–N_PYA_/Å	2.044(2)	2.006(10)
Pd–Cl_*trans*_/Å	2.2942(7)	2.299(3)
Pd–Cl_*cis*_/Å	2.2812(6)	2.2794(9)
N_PYA_–C_α_/Å	1.385(3)	1.377(10)
N_PYA_–C_CO_/Å	1.362(3)	1.383(11)
%*V*_bur_^a^	42.3	42.3
*α*/°^b^	123.44(18)	121.0(8)
*τ*_4_ c	0.086	0.074

a Percentage buried volume %*V*_bur_ determined according to ref. 40.

b The *α* angle was determined as angle N_prz_–N_prz2_–C_R_ (see also Fig. 1d).

c Tetrahedral distortion parameter *τ*_4_ determined according to ref. 38.

The functionalized pyrazolyl complexes 5e–5g were evaluated in catalytic propiophenone arylation applying the same conditions that were used for complex 5d (Table 4, Fig. S65†). All pyrazole-substituted complexes 5d–f exhibited higher catalytic activity compared to the parent pyridyl–PYA precatalyst 5a, with TOF_max_ values ranging from 420 to 1180 h^−1^ as opposed to 130 h^−1^ for 5a. However, this increased activity did not always result in improved selectivity. Only the alkyl-substituted pyrazolyl complexes 5d and 5g demonstrated superior selectivity, achieving 91% and >99% yield, respectively, compared to 87% for 5a (Table 4*vs.*Table 2, entry 1). While there is no steric correlation between the pyrazolyl substituent and catalytic activity nor yield, there is an obvious correlation with the electronic properties of the R group with TOF_max_ increasing along the series 5e < 5f < 5d < 5g. Thus, electron-donating alkyl groups induce higher activity than aryl substituents, and within both the aryl and the alkyl substituents, the more donating xylyl and iPr groups impart better performance than Ph and Me, respectively. The most active catalyst derived from 5g reached full conversion in just 6 min at 105 °C and afforded quantitative yields, which is rather rare in ketone α-arylation catalysis^75–77^ and indicates a very effective suppression of side reactions by the iPr substituted pyrazolyl–PYA ligand.^14,15,78–80^ The beneficial role of strong donor ligands corroborates the conclusions from studies focusing on modulation of the PYA unit of 5a.^30^ Mechanistically, this behavior may be rationalized with the higher *trans* effect of stronger donor ligands, which labilizes the bromide ligand after ArBr oxidative addition^81^ and hence facilitates enolate binding.

**Table 4:** Propiophenone α-arylation with pyrazole-PYA palladium complexes 5d–5g^a^

Entry	Catalyst	R	Optimized yield^b^	Yield	TOF_max_/h^−1^	*k*_ini_/h^−1^
1	5d	Me	91%	84%	830	8.3
2	5e	Ph	79%	72%	420	4.2
3	5f	Xyl	83%	74%	730	8.1
4	5g	iPr	>99%	91%	1200	15

a Reaction conditions: PhBr (1.0 mmol), propiophenone (1.2 mmol), [Pd] (0.01 mmol), NaO*t*Bu (1.32 mmol), 1,4-dioxane (2.0 mL), 105 °C under N_2_, 60 min; yield determined by GC analysis using hexamethylbenzene as internal standard; for TOF_max_ evaluations see Fig. S65;† for calculation of initial rate constant *k*_ini_ see Fig. S66.†

b Optimized yields determined without sampling, using identical conditions except propiophenone (1.0 mmol) NaO*t*Bu (1.1 mmol), dioxane (1.0 mL).

Interestingly, the improved selectivity derived from steric shielding of the imine side contrasts with the reactivity trend observed when substituents were introduced to the pyridine in 5a.^37^ Introduction of a bulky xylyl group at the pyridyl 6-position resulted in a more active yet less selective catalyst. A reduced selectivity may be rationalized by less efficient enolate transmetalation due to the more prominent orientations of the pyridyl substituents into the catalytically relevant space, as juxtaposed by the pertinent *α*-angles.

To assess the potential of the most active catalyst 5g, the model reaction was performed under various conditions that are typically posing challenges for palladium-catalyzed α-arylation (Table 5). For example, catalysis under an atmosphere of air instead of N_2_ gave still decent activity with complex 5g (82% conversion *vs.* 99% under N_2_, entries 1, 2).^82^ In contrast, complex 5a is only poorly active under these conditions (27%, entry 3).^37^ Running the reaction in bench-grade dioxane that was stored in air and contained 0.3% H_2_O according to a Karl-Fischer titration gave 80% product (entry 4), similar to reactions performed in air and indicating a high robustness of the catalytic system towards air and moisture. This robustness is particularly relevant when considering that dry solvents and anaerobic conditions contribute significantly to the overall costs of a synthetic process. Reducing the reaction temperature from 105 to 60 °C afforded almost quantitative yields, albeit with a considerable extension of the reaction time to 72 h (entry 5). While complex 5a reached low TONs with (activated) aryl chlorides, complex 5g was inactive towards these substrates, even upon increasing the reaction temperature to 125 °C (entries 6, 7). A competition experiment using equimolar quantities of bromo- and chlorobenzene revealed almost full conversion of the aryl bromide exclusively (entry 8), indicating that aryl chlorides are catalytic bystanders without any significant inhibition effect. Such behavior is further confirmed by the chemoselective transformation of 1-bromo-4-chlorobenzene with full retention of the chloride (entry 9, Fig. S85†), which might be a desirable feature for late-stage functionalization and synthetic diversification.^83–86^ Notably, phosphine- and NHC-based palladium catalysts lack such selectivity between aryl bromides and chlorides.^21,87^ Reducing the catalyst loading to 100 ppm resulted in a lowered 50% yield (entry 10). While this outcome corresponds to a respectable 5000 TON with a TOF_max_ = 2300 h^−1^, the incomplete 69% conversion of PhBr suggests gradual deactivation of the catalyst.

**Table 5:** Catalytic activity of 5g under selected more challenging conditions^a^

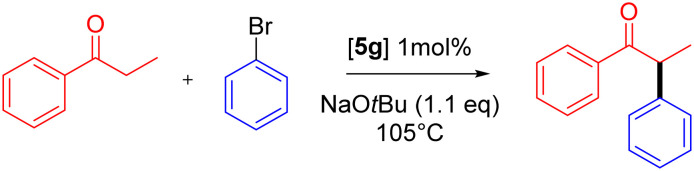			
Entry	Deviation from above	Yield	Reaction time/h
1	None	>99%	1.0
2	Air instead of N_2_ atmosphere	82%	1.5
3	Air instead of N_2_, 5a instead of 5g	27%	1.5
4	Moist dioxane (0.3% H_2_O)^b^	80%	1.5
5	60 °C instead of 105 °C	92%	72
6	PhCl instead of PhBr^c^	<1%	1.5
7	4-CF_3_–C_6_H_4_Cl instead of PhBr^c^	<1%	1.5
8	PhCl (100 mol%) as additive	80%	1.5
9	4-Cl–C_6_H_4_Br instead of PhBr	88%	1.5
10	100 ppm 5g, 125 °C	50%	24

a General reaction conditions: aryl halide (1.0 mmol), propiophenone (1.0 mmol), 5g (0.01 mmol), NaO*t*Bu (1.1 mmol), dry 1,4-dioxane (2.0 mL) in a 10 mL vial, 105 °C under N_2_, yield determined by GC analysis using hexamethylbenzene as internal standard.

b Determined by Karl-Fischer titration.

c ArCl (1.2 mmol), no conversion also at 125 °C.

**Fig. 7:**
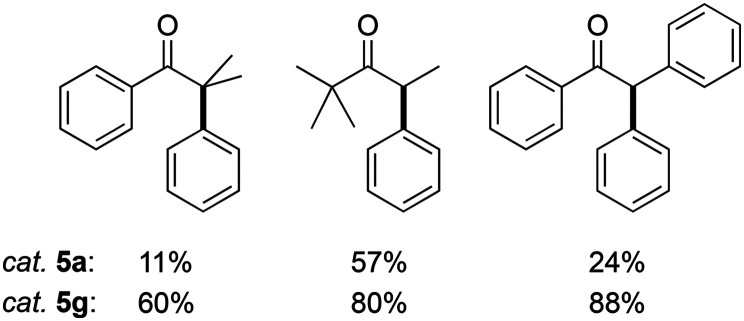
Products from α-arylation of challenging ketones under standard reaction conditions, *i.e.*, phenyl bromide (1.0 mmol), ketone (1.0 mmol), 5g (0.01 mmol), NaO*t*Bu (1.1 mmol), dry 1,4-dioxane (2.0 mL), 105 °C under N_2_, 2 h.

Moreover, the enhanced activity of 5g compared to 5a was confirmed by comparing the catalytic performance with challenging substrates (Fig. 7). Under standard conditions and using PhBr as the arylating agent, 5g gave consistently higher product yields than 5a. For example, phenyl acetophenone was arylated by 5a in a modest 24% yield, while 5g induced 88% of α-arylation within 2 h. This enhanced activity also reinforces enolate transmetalation as critical step on the catalytic cycle.

## Conclusions

By using the synthetic versatility of PYA functionalization, a set of new palladium(ii) complexes were prepared containing a *N*,*N′*-bidentate chelating PYA ligand substituted with a N-heterocyclic imine donor. While oxazole functionalization of the PYA ligand results in lower catalytic activity in ketone α-arylation, triazole and especially pyrazole donors increase the activity. Tailoring of the N-substituent of the pyrazole provided complex 5g featuring catalytic activity that is about 10 times higher than that of the parent complex with a pyridine–PYA ligand. Moreover, the selectivity of the arylation approaches an ideal pathway with quantitative yields, and therefore provides a catalyst precursor that is competitive to some of the best systems based on phosphine or NHC ligands. Mechanistic work including kinetic experiments, catalyst poisoning studies, and catalyst recycling supports a homogeneous reaction trajectory with a molecularly defined species as active catalyst and an oxygen-sensitive catalyst resting state. These conclusions underpin the relevance of ligand design and advocate for further optimization of the chelating PYA ligand scaffold in order to address limitations of 5g such as the inactivity towards aryl chlorides and the increase of turnover numbers. Notably, these principles also warrant the development of new N-based ligand systems for improving other challenging C–C bond formation catalysis.

## Data availability

The data supporting this article have been included as part of the ESI.†

Crystallographic data for compounds 5b, 5d, 5f, and 5g has been deposited at the CCDC under No. 2394912–2394915 and can be obtained from https://www.ccdc.cam.ac.uk free of charge.

## Conflicts of interest

The authors declare no competing financial interest.

## Supplementary Material

CY-015-D4CY01337A-s001

CY-015-D4CY01337A-s002
