# Supplementary material for: Enhancing activity and selectivity of palladium catalysts in ketone α-arylation by tailoring the imine chelate of pyridinium amidate (PYA) ligands
Source: Catal Sci Technol. 2024 Dec 18;15(3):867–77. doi: 10.1039/d4cy01337a (PMC11701426; doi:10.1039/d4cy01337a)
Supplement: CY-015-D4CY01337A-s001 [file CY-015-D4CY01337A-s001.pdf]

Supplementary Information (SI) for Catalysis Science & Technology.
This journal is © The Royal Society of Chemistry 2024

Electronic Supporting Information

belonging to

Enhancing activity and selectivity of palladium catalysts in ketone α-arylation by tailoring the imine chelate of pyridinium amidate (PYA) ligands

Esaïe Reusser[a], Michael Aeschlimann[a] and Martin Albrecht*[a]

[a] Department of Chemistry, Biochemistry and Pharmaceutical Sciences, University of Bern, Freiestrasse 3, 3012 Bern, Switzerland

Email: martin.albrecht@unibe.ch

Contents

S.1 Synthetic procedures 3

S.1.1 General information 3

S.1.2 Synthesis of precursor esters 4

S.1.3 Synthesis of carboxylic acids **1c–1g** 9

S.1.4 Synthesis of pyridine carboxamides **2a–2f** 14

S.1.5 Synthesis of pyridinium iodide salts **3a–3g** 23

S.1.6 Synthesis of pyridinium amidate (PYA) **4a–4g** 33

S.1.7 Synthesis of PYA palladium complexes **5a–5g** 43

S.2 Crystal structure determinations 54

S.3 Buried volume calculations 58

S.4 Catalytic procedures 62

S.4.1 General procedure for propiophenone α-arylation 62

S.4.2 Procedure for the acquisition of time-conversion profiles and TOF_{max} determination 62

S.4.3 General procedure for determining the order in palladium complex 65

S.4.4 Procedure for determining the rate order in substrates 69

S.4.5 Procedures for catalyst poisoning experiments 71

S.4.6 General procedure for temperature screening experiments 73

S.4.7 Procedure for evaluating air and moisture tolerance 75

S.4.8 Procedure for substrates refilling experiment 75

S.4.9 Procedure for catalyst recycling experiments 75

S.4.10 Studies on catalyst activation using **5d** 77

S.4.11 General procedure for aryl chloride activation and competitive C–Br vs C–Cl conversion 80

S.5 References 84

S.1 Synthetic procedures

S.1.1 General information

Phenyl azide and complex **5a** were prepared according to previously reported procedures.[S1,S2] MeCN, tetrahydrofuran (THF) and CH_2Cl_2 were dried by passage through a solvent purification column. All other reagents and solvents were purchased from commercial vendors and used as received. Unless otherwise stated, all the reactions were run under exclusion of air using standard Schlenk techniques under an atmosphere of dry nitrogen and extra dry, stabilized 1,4-dioxane (99.8%) purchased from Thermoscientific was used for catalysis.

Unless otherwise specified, NMR spectra were recorded at 25 °C on Bruker spectrometers operating at 300 or 400 MHz (^1H NMR) and 75 or 101 MHz ($^{13}C\{^1H\}$ NMR), respectively. Chemical shifts (δ) are expressed in ppm downfield from $SiMe_4$ using the residual protonated solvent as an internal standard and coupling constants in Hz. Assignments are based on homo- and heteronuclear shift correlation spectroscopy.

High-resolution mass spectrometry was carried out with a Thermo Scientific LTQ Orbitrap XL (ESI-TOF) or a Thermo Scientific Q Exactive GC directly used with a direct injection probe (DIP). Elemental analyses were performed on a Thermo Scientific Flash 2000 CHNS-O elemental analyzer.. GC-FID reaction follow-up was achieved using an Agilent technologies 7820A GC System. GC-MS reaction follow-up was carried out on a Thermofisher ISQ7000 GC fitted with a Macherey-Nagel Optima delta-3-0.25 μm capillary column using Helium as a carrier gas, coupled to a quadrupole mass analyzer using EI mode at 70 eV.

S.1.2 Synthesis of precursor esters

Synthesis of prec-c

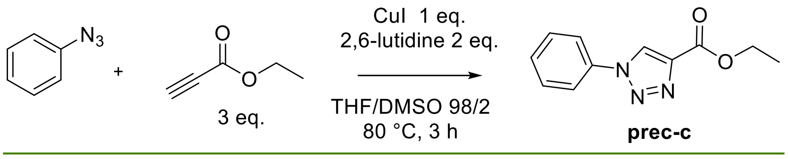

Phenyl azide (1.67 g, 14.0 mmol), copper(I) iodide (2.67 g, 14.0 mmol), THF (15 mL), and DMSO (0.3 mL) were charged in a pressure flask. Then 2,6-lutidine (3.25 mL, 28.0 mmol) was added followed by ethyl propiolate (4.26 mL, 42.0 mmol). The resulting mixture was stirred at 80 °C for 3 h. After cooling to room temperature the reaction mixture was poured into H_2O (50 mL) and extracted with EtOAc (3 x 30 mL). The combined organic layers were washed with HCl_{aq} (1 M, 100 mL) and brine (100 mL), dried over $MgSO_4$, concentrated to dryness, triturated with pentane (3 x 10 mL) and dried under vacuum to yield **prec-c** as an off-white solid. (2.46 g, 81%). Spectroscopic data are in agreement with literature values.[S3] ^{1}H NMR (300 MHz, DMSO-d_6, 298K) δ = 9.48 (s, 1H, CH_{trz}), 7.99 (dd, J_{HH} = 7.6, 1.8 Hz, 2H, CH_{Ph}), 7.57 (m, 3H, CH_{Ph}), 4.36 (q, $^3J_{HH}$ = 7.1 Hz, 2H, CH_2), 1.34 (t, $^3J_{HH}$ = 7.1 Hz, 3H, CH_3) ppm.

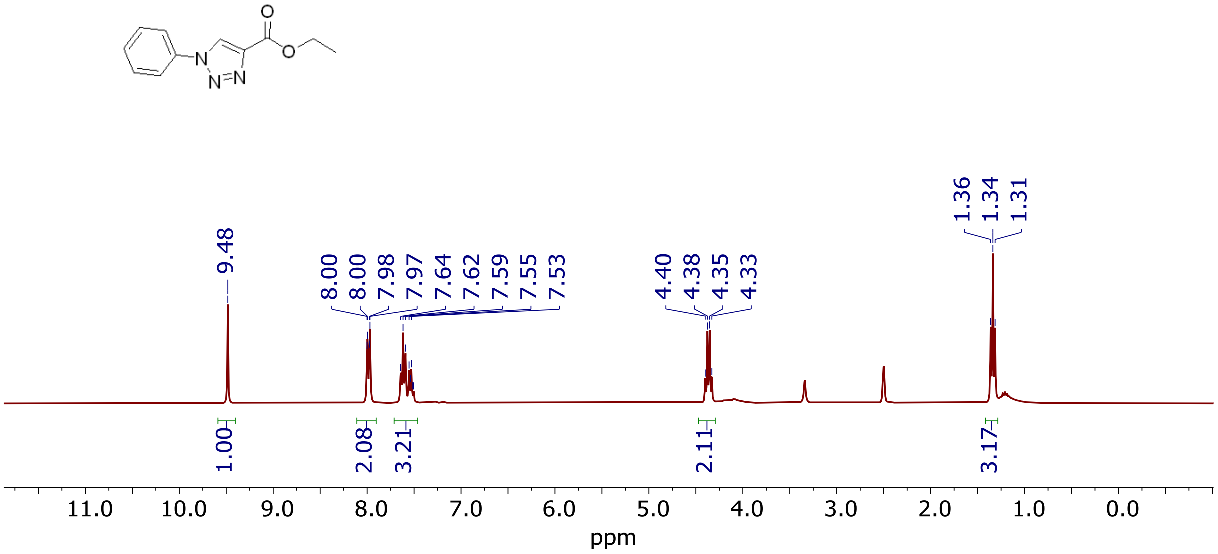

Figure S1: ^{1}H NMR spectrum (DMSO-d_6, 298 K, 300 MHz) of **prec-c**.

Synthesis of prec-e

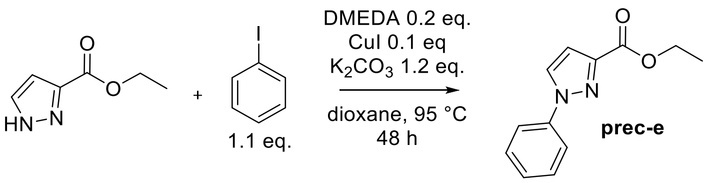

Ethyl 1H-pyrazole-3-carboxylate (3.00 g, 21.4 mmol), potassium carbonate (3.55 g, 25.7 mmol), and copper(I) iodide (408 mg, 2.14 mmol) were charged in a two-neck flask equipped with a condenser. The flask was then placed under a nitrogen atmosphere and 1,4-dioxane (30 mL) was added, followed by N,N'-dimethylethane-1,2-diamine (460 µL, 4.28 mmol) and iodobenzene (2.63 mL, 23.5 mmol). The resulting suspension was heated under vigorous stirring to 95 °C for 48 h. After cooling to room temperature, the suspension was washed with NH_4Cl_{aq} (sat., 2 x 100 mL) and brine (50 mL). The organic phase was concentrated under vacuum, and the residue was purified by gradient flash column chromatography (FCC; SiO_2; EtOAc:pentane, 0 to 100%) to yield **prec-e** as a yellow oil (3.68 g, 79 %). ^{1}H NMR (300 MHz, DMSO-d_6, 298K) δ = 8.63 (d, $^3J_{HH}$ = 2.6 Hz, 1H, CH_{prz}), 7.94 – 7.82 (m, 2H, CH_{phe}), 7.57 – 7.40 (m, 2H, CH_{phe}), 7.46 – 7.34 (m, 1H, CH_{phe}), 7.01 (d, $^3J_{HH}$ = 2.6 Hz, 1H, CH_{prz}), 4.33 (q, $^3J_{HH}$ = 7.0 Hz, 2H, CH_2), 1.32 (t, $^3J_{HH}$ = 7.0 Hz, 3H, CH_3) ppm. ^{13}C{^{1}H} NMR (75 MHz, DMSO-d_6, 298K) δ = 161.48 (*C*O), 144.29 (*C*$_{prz}$), 139.16 (*C*$_{phe}$), 129.83 (*C*H$_{prz}$), 129.67 (*C*H$_{phe}$) 127.48 (*C*H$_{phe}$), 119.22 (*C*H$_{phe}$), 110.20 (*C*H$_{prz}$), 60.49 (*C*H$_2$), 14.21 (*C*H$_3$) ppm. HR-MS (m/z): calculated for $C_{12}H_{13}N_2O_2$ $[M+H]^+$ = 217.0977; found: 217.0968.

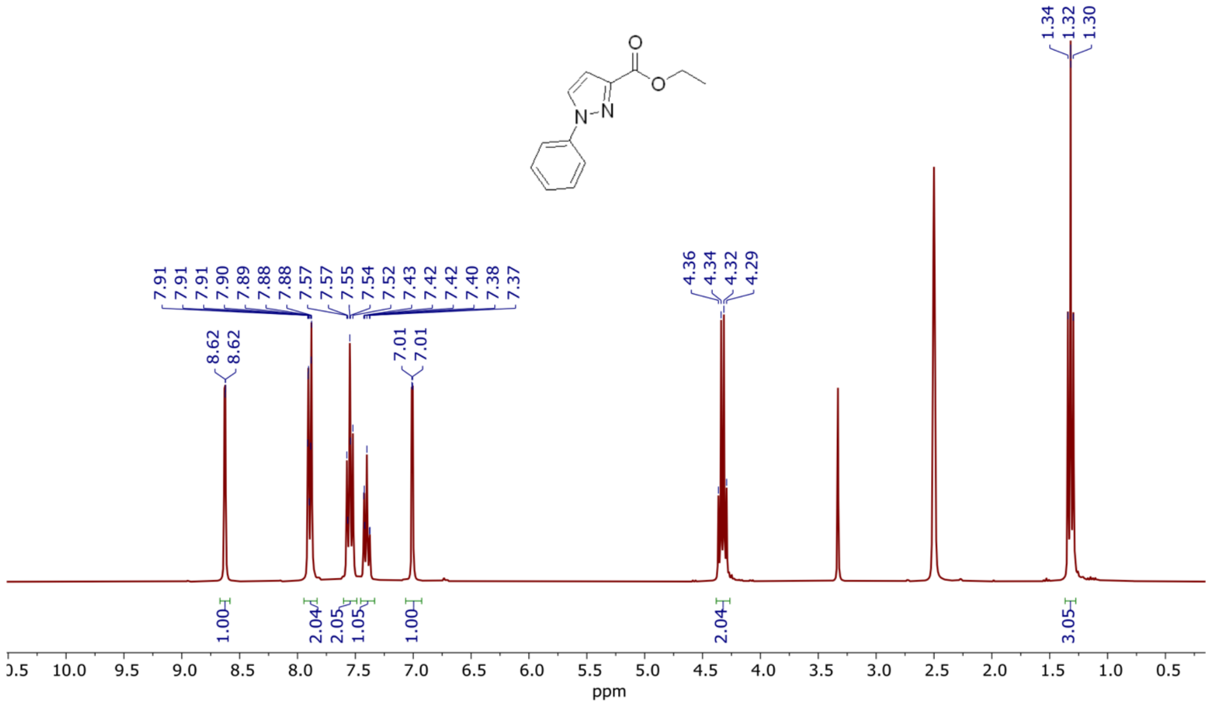

Figure S2: ^{1}H NMR spectrum (DMSO-d_6, 298 K, 300 MHz) of **prec-e**.

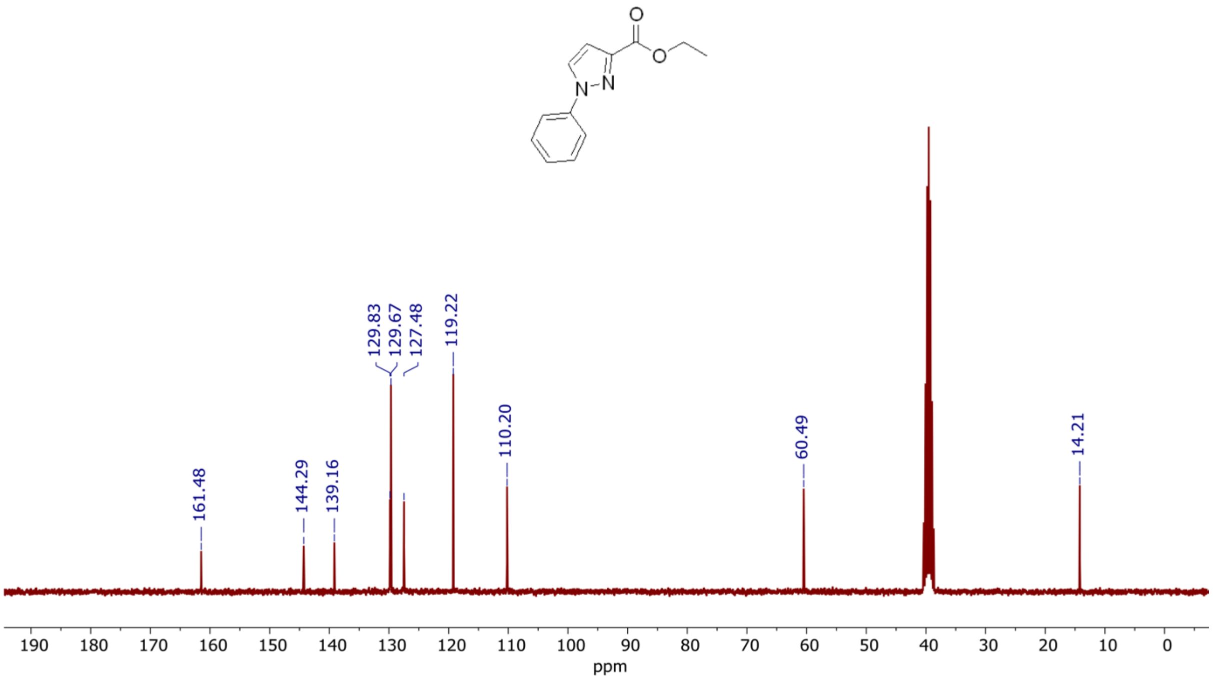

Figure S3: $^{13}C\{^1H\}$ NMR spectrum (DMSO-d_6, 298 K, 75 MHz) of **prec-e**.

Synthesis of prec-f

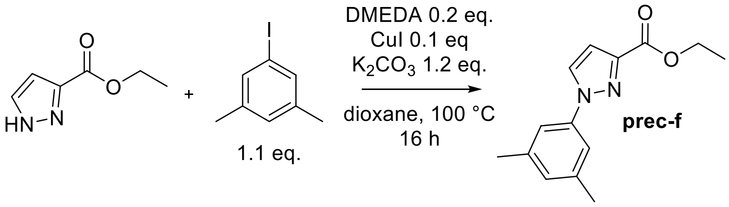

Ethyl 1H-pyrazole-3-carboxylate (2.00 g, 14.3 mmol), potassium carbonate (2.37 g, 17.1 mmol), copper(I) iodide (272 mg, 1.43 mmol) were charged in a 20 mL vial then 1,4-dioxane (10 mL), N^1,N^2-dimethylethane-1,2-diamine (307 µL, 2.85 mmol) and iodobenzene (2.27 mL, 15.7 mmol) were added under N_2. The resulting suspension was heated to 100°C and was vigourously stirred at that temperature for 16 h. After cooling down to room temperature, the suspension was washed twice with NH_4Cl_{aq} (sat., 2 x 50ml) and brine (25 mL). The organic phase was then concentrated under vacuo and purified by gradient FCC (SiO_2; EtOAc:pentane, 0 to 100%; rf 0.8 in 25 % EtOAc; 3rd fraction) to yield **prec-f** (3.16 g, 90 %) as a white powder. ^1H NMR (300 MHz, CD_3CN, 298K) δ = 8.15 (d, $^3J_{HH}$ = 2.6 Hz, 1H, CH_{prz}), 7.43 (s, 2H, CH_{xyl}), 7.06 (s, 1H, CH_{xyl}), 6.95 (d, $^3J_{HH}$ = 2.6 Hz, 1H, CH_{prz}) 4.37 (q, $^3J_{HH}$ = 7.1 Hz, 2H, CH_2), 2.39 (s, 6H, xylyl-CH_3), 1.38 (t, $^3J_{HH}$ = 7.1 Hz 3H, CH_2CH_3) ppm. $^{13}C\{^1H\}$ NMR (75 MHz, CD_3CN, 298K) δ = 162.57 (*C*O), 145.72 (C_{prz}), 140.69 (C_{xyl}-Me), 140.63 (C_{phe}), 130.01(CH_{xyl}), 129.56 (CH_{prz}), 118.15 (CH_{xyl}), 110.81 (CH_{prz}), 61.67 (CH_2), 21.35 (xylyl-CH_3), 14.62 (CH_2CH_3) ppm. HR-MS (m/z): calculated for $C_{14}H_{17}N_2O_2$ $[M+H]^+$ = 245.1290; found: 245.1280.

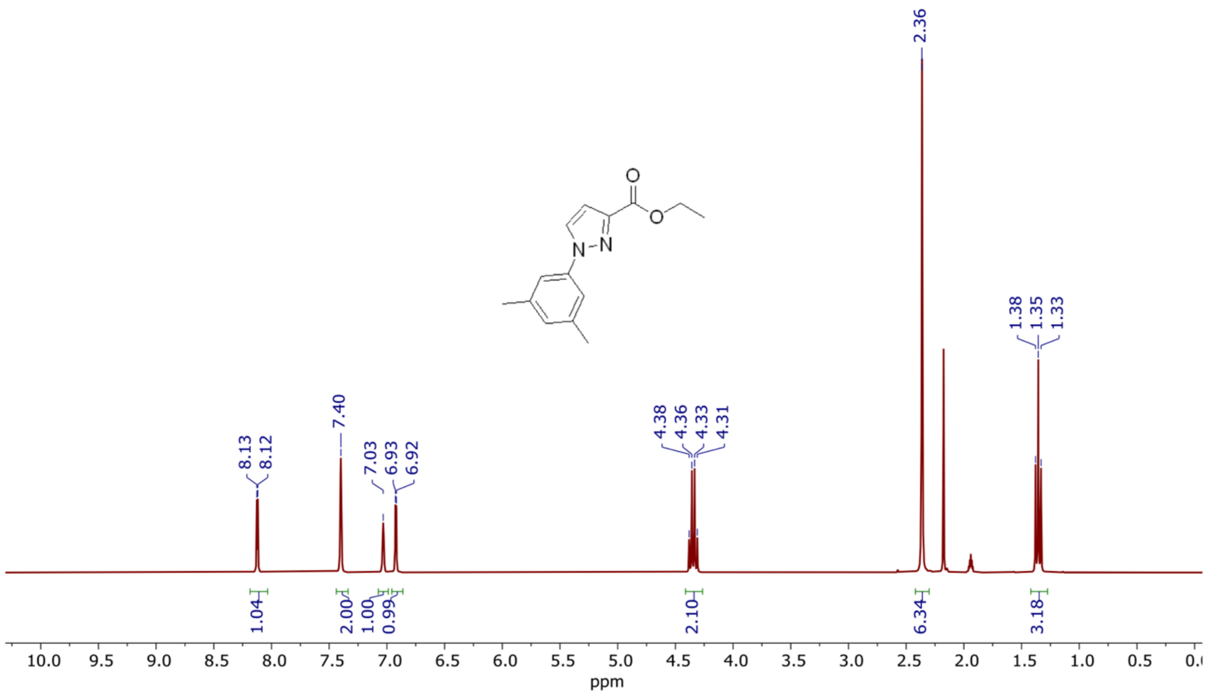

Figure S4: ^{1}H NMR spectrum (CD_3CN, 298 K, 300 MHz) of **prec-f**.

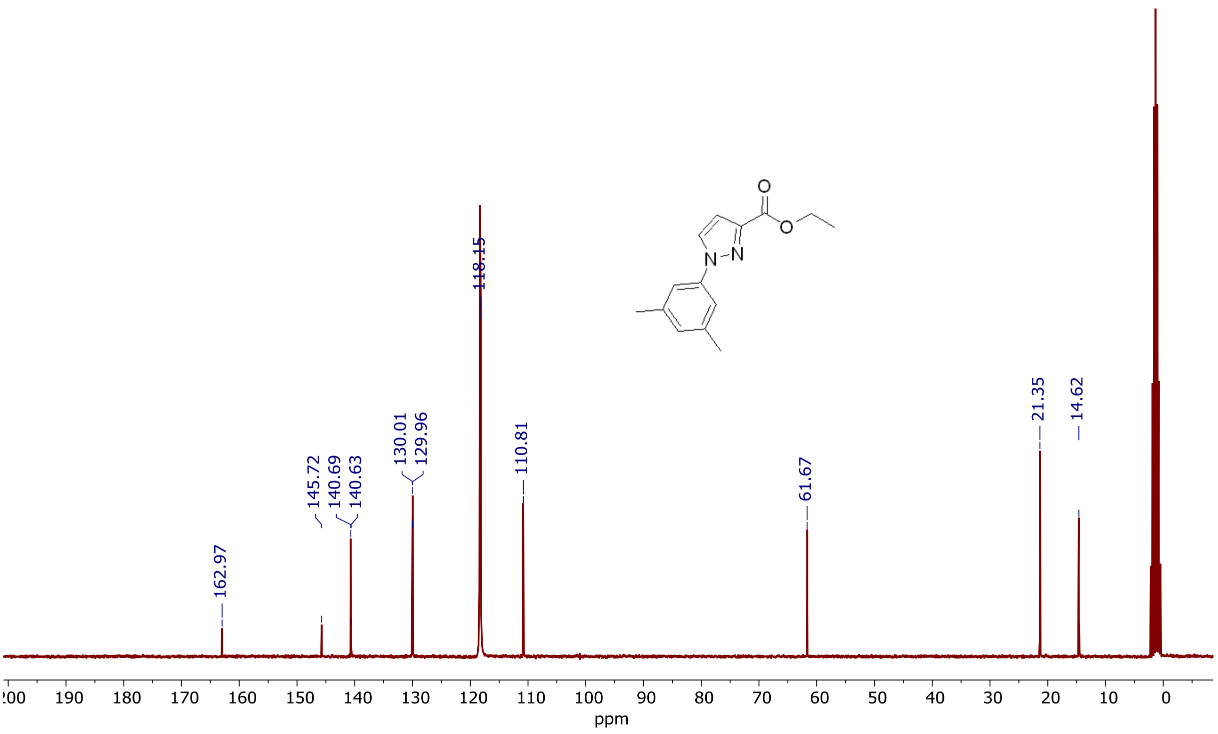

Figure S5: ^{13}C{^{1}H} NMR spectrum (CD_3CN, 298 K, 75 MHz) of **prec-f**.

Synthesis of prec-g

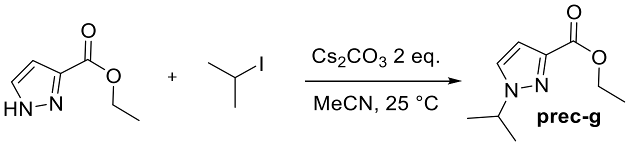

Ethyl 1H-pyrazole-3-carboxylate (1.5 g, 10.7 mmol) and Cs_2CO_3 (6.9 g, 21.4 mmol) were dissolved in dry MeCN (40 mL) under constant N_2 flow. 2-iodopropane (1.1 mL, 10.7 mmol) was added and the mixture stirred at rt for 16 h. The reaction mixture was then concentrated under vacuo followed by gradient FCC (SiO_2; EtOAc:pentane, 0 to 30%; 2nd fraction) to yield **prec-g** as a colorless oil (0.80 g, 41%). Note: **prec-g** is also commercially available. The ^{1}H NMR data (Figure S6) match those of commercial samples and purity is sufficient for further reactions.[S4] ^{1}H NMR (300 MHz, CD_3CN, 298K) δ = 7.63 (d, $^3J_{HH}$ = 2.4 Hz, 1H, CH_{prz}), 6.74 (d, $^3J_{HH}$ = 2.4 Hz, 1H, CH_{prz}), 4.59 (hept, $^3J_{HH}$ = 6.7 Hz, 1H, *i*Pr-C*H*), 4.31 (q, $^3J_{HH}$ = 7.1 Hz, 2H, CH_2), 1.49 (d, $^3J_{HH}$ = 6.7 Hz, 6H, *i*Pr-CH_3), 1.34 (t, $^3J_{HH}$ = 7.1 Hz 3H, CH_2CH_3) ppm.

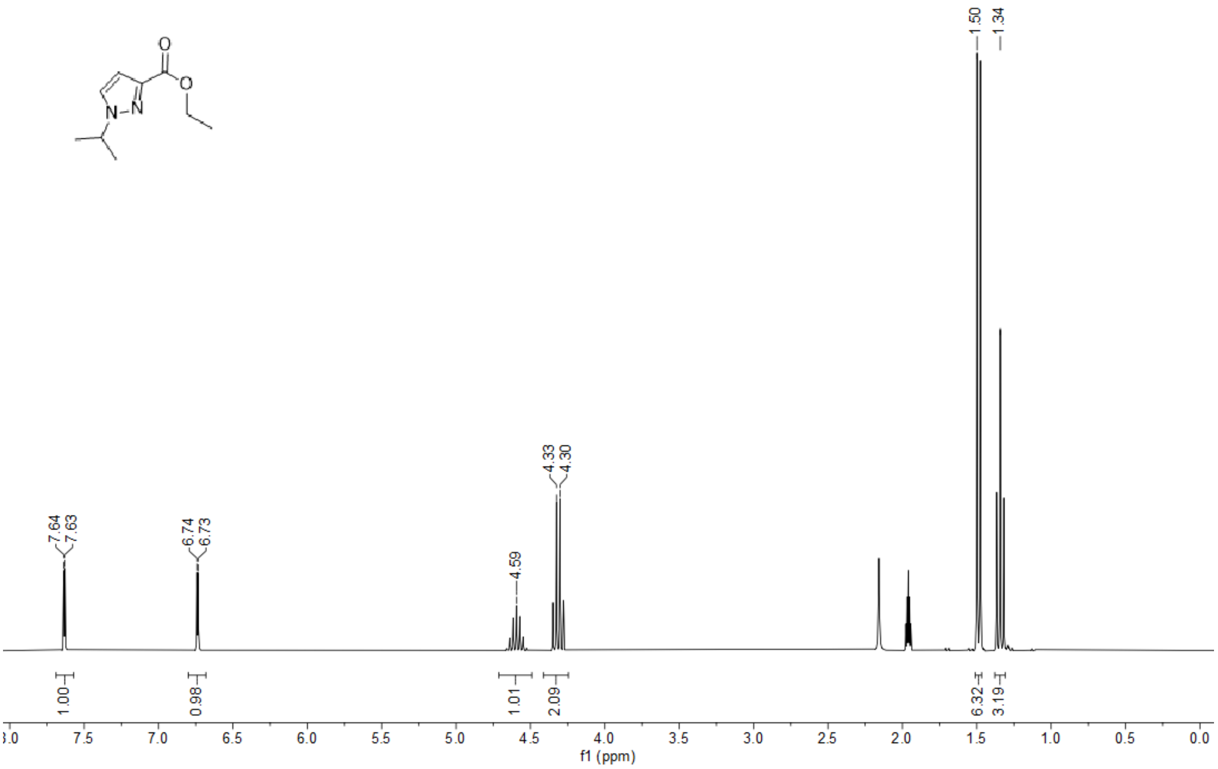

Figure S6: ^{1}H NMR spectrum (CD_3CN, 298 K, 300 MHz) of **prec-g**.

S.1.3 Synthesis of carboxylic acids 1c–1g

Synthesis of 1c

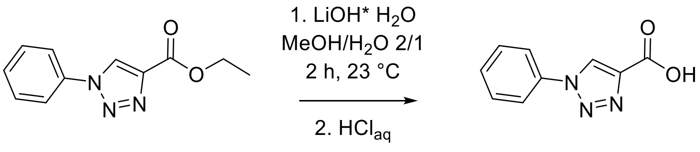

prec-c **1c**

Prec-c (1.5 g, 6.9 mmol) and lithium hydroxide monohydrate (0.58 g, 13.8 mmol) were stirred for 1 h at 23 °C in a mixture of MeOH (10 mL) and H_2O (5 mL). At full conversion (TLC monitoring), HCl_{aq} (1.0 M, 15 mL) was added slowly and the resulting mixture was stirred for 20 min. The reaction mixture was then extracted with CH_2Cl_2 (3 x 30 mL), the organic fractions were combined, dried over $MgSO_4$, filtered, and concentrated to dryness to yield **1c** as a off-white solid (1.1 g, 84%). Spectroscopical data are in agreement with literature values.[S5] ^{1}H NMR (300 MHz, DMSO-d_6, 298K) δ = 13.30 (s, 1H, COO*H*), 9.39 (s, 1H, CH_{trz}), 7.98 (dd, J_{HH} =7.7, 1.7 Hz, 2H, CH_{Ph}), 7.68 – 7.47 (m, 3H, CH_{Ph}) ppm.

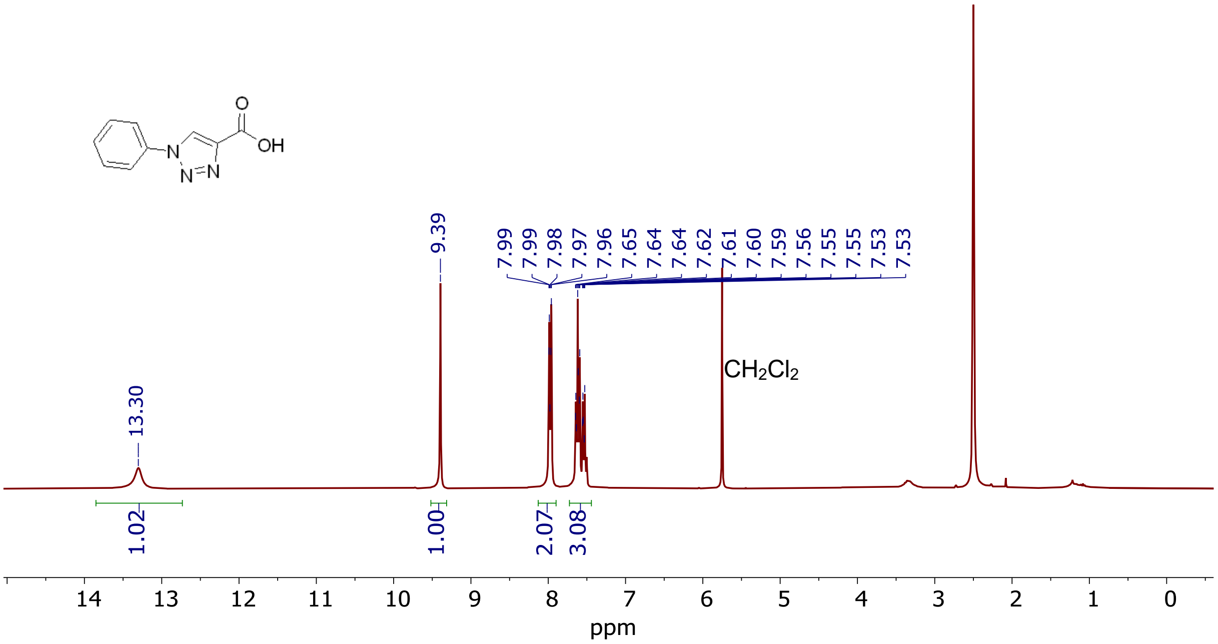

Figure S7: ^{1}H NMR spectrum (DMSO-d_6, 298 K, 300 MHz) of **1c**.

Synthesis of 1e

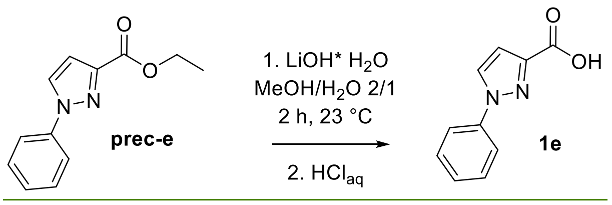

Prec-e (3.33 g, 15.4 mmol) and lithium hydroxide monohydrate (1.29 g, 30.8 mmol were stirred for 1 h at 23 °C in a mixture of MeOH (10 mL) and H_2O (5 mL). At full conversion (TLC monitoring), HCl_{aq} (1.0 M, 35 mL) was added slowly and the resulting mixture was stirred for 20 min. The reaction mixture was then extracted with CH_2Cl_2 (3 x 30 mL), the organic fractions were combined, dried over $MgSO_4$, filtered, and concentrated to dryness to yield **1e** as an off-white solid (2.68 g, 92%). ^{1}H NMR (300 MHz, DMSO-d_6, 298K) δ = 12.97 (s, 1H, COO*H*), 8.61 (d, $^3J_{HH}$ = 2.6 Hz, 1H, CH_{prz}), 7.95 – 7.84 (m, 2H, CH_{phe}), 7.62 – 7.49 (m, 2H, CH_{phe}), 7.46 – 7.34 (m, 1H, CH_{phe}), 6.96 (d, $^3J_{HH}$ = 2.6 Hz, 1H, CH_{prz}), ppm. ^{13}C{^{1}H} NMR (75 MHz, DMSO-d_6, 298K) δ = 163.05 (*C*O), 145.28 (C_{prz}), 139.33 (C_{phe}), 129.76 (CH$_{phe}$), 129.71 (CH$_{prz}$), 127.44 (CH$_{phe}$), 119.20 (CH$_{phe}$), 110.32 (CH$_{prz}$). HR-MS (m/z): calculated for $C_{10}H_9N_2O_2$ [M+H]$^+$ = 189.0664; found: 189.0654.

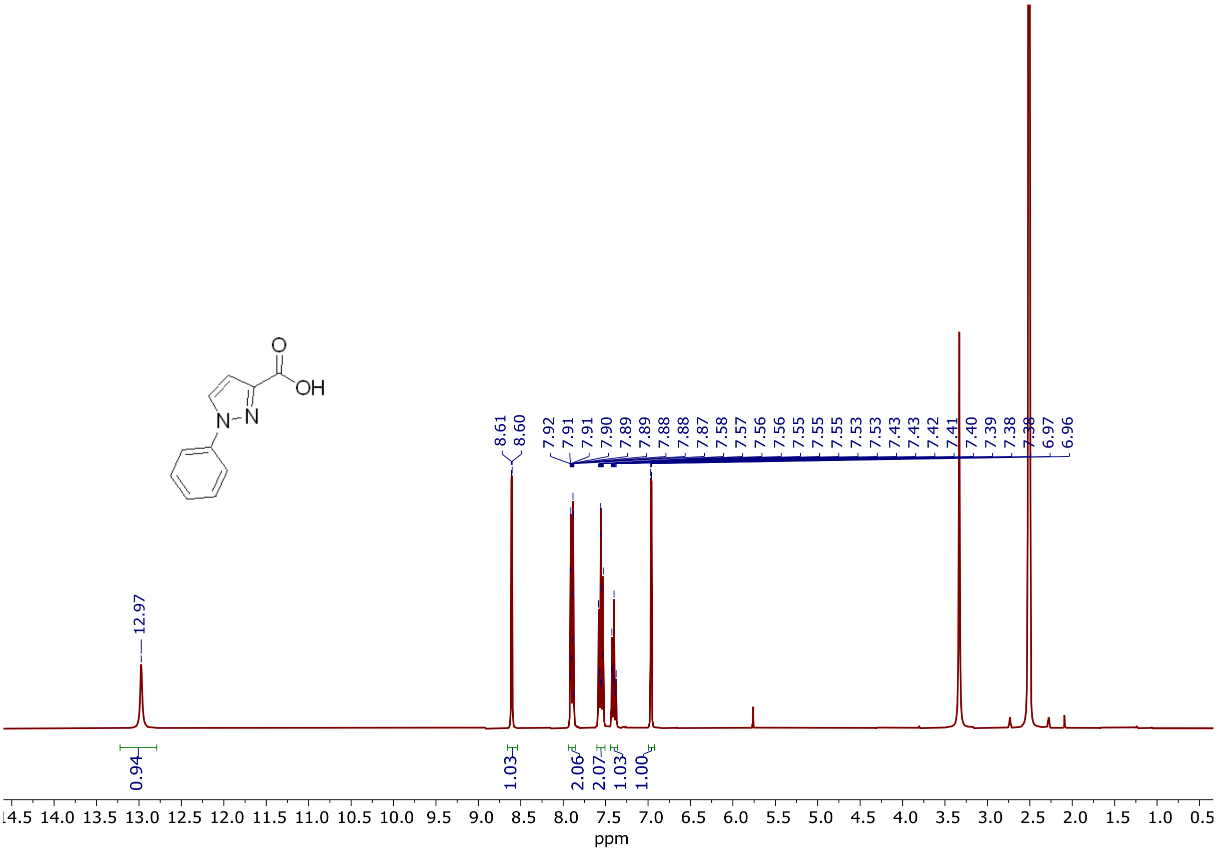

Figure S8: ^{1}H NMR spectrum (DMSO-d_6, 298 K, 300 MHz) of **1e**.

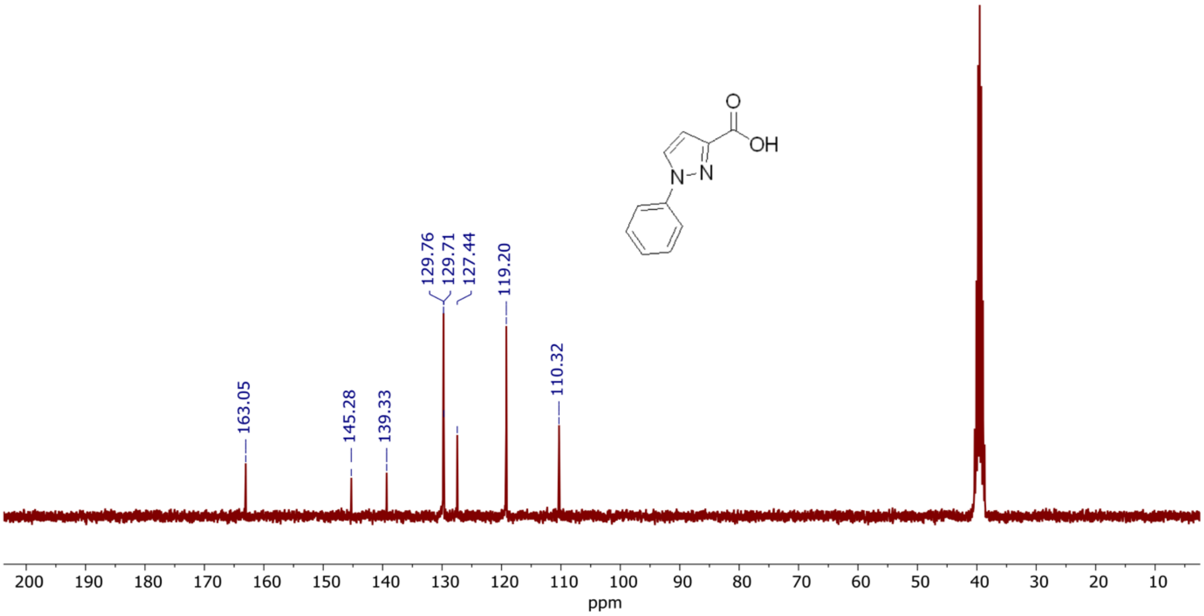

Figure S9: $^{13}C\{^1H\}$ NMR spectrum (DMSO-d_6, 298 K, 75 MHz) of **1e**.

Synthesis of 1f

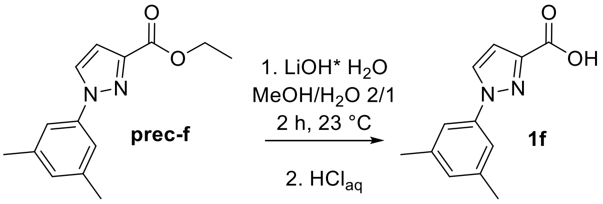

Prec-f (2.80 g, 11.5 mmol) and lithium hydroxide monohydrate (0.96 g, 23.0 mmol were stirred for 1h at 23 °C in a mixture of MeOH (20 mL) and H_2O (10 mL). At full conversion (TLC monitoring), HCl_{aq} (1.0 M, 25 mL) was added slowly and the resulting mixture was stirred for 20 more min. The reaction mixture was then extracted in CH_2Cl_2 (3 x 30 mL), the organic fractions were combined, dried over $MgSO_4$, filtered, and concentrated to dryness to yield **1f** as a white solid (2.43 g, 98%). ^1H NMR (300 MHz, DMSO-d_6, 298K) δ = 12.94 (s, 1H, COO*H*), 8.53 (d, $^3J_{HH}$ = 2.6 Hz, 1H, CH_{prz}), 7.51 (s, 2H, CH_{xyl}), 7.01 (s, 1H, CH_{xyl}), 6.92 (d, $^3J_{HH}$ = 2.6 Hz, 1H, CH_{prz}) 2.35 (s, 6H, xylyl-CH_3) ppm. $^{13}C\{^1H\}$ NMR (75 MHz, DMSO-d_6, 298K) δ = 163.01 (*C*OOH), 144.98 (C_{prz}), 139.23 (C_{xylyl}), 139.07 (C_{xyl}-Me), 129.49 (CH_{phe}), 128.69 (CH_{prz}), 116.78 (CH_{xyl}), 110.07 (CH_{prz}), 20.92 (xylyl-CH_3) ppm. HR-MS (m/z): calculated for $C_{12}H_{13}N_2O_2$ $[M+H]^+$ = 217.0977; found: 217.0968.

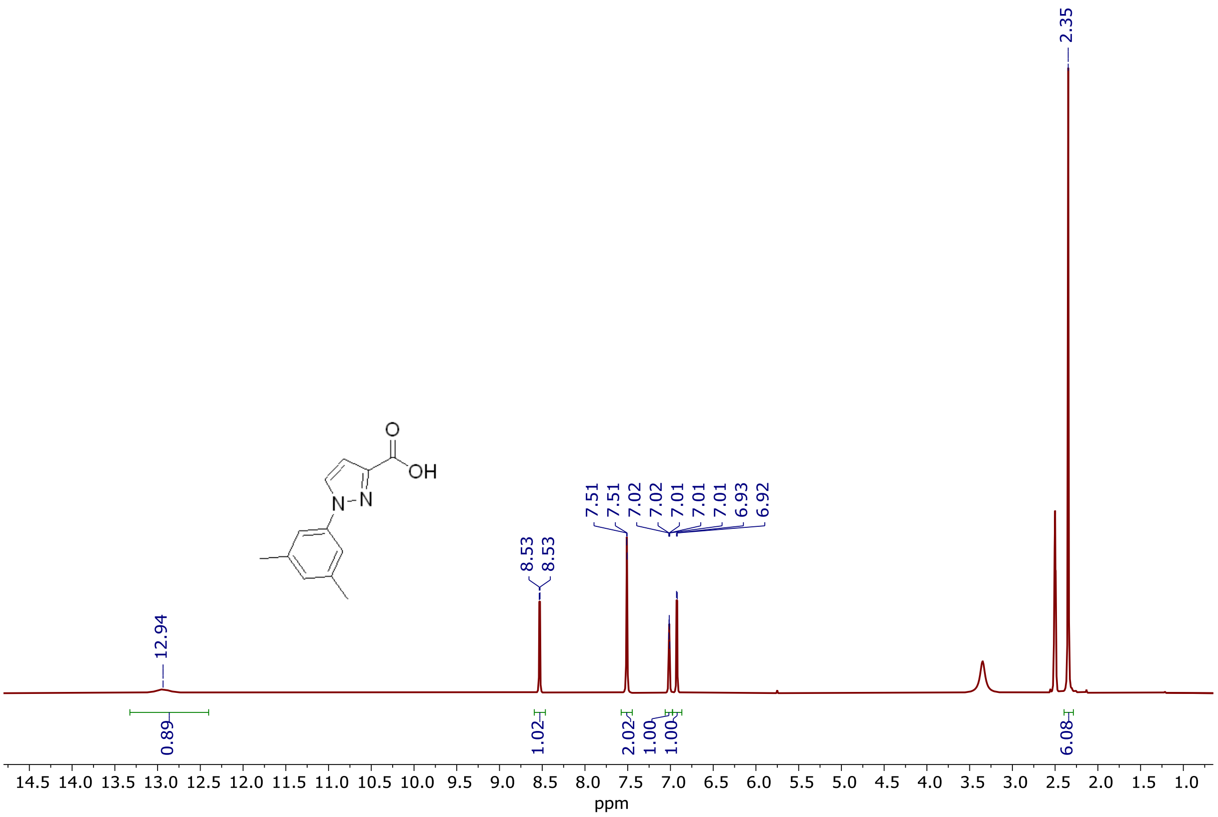

Figure S10: ^{1}H NMR spectrum (DMSO-d_6, 298 K, 300 MHz) of **1f**.

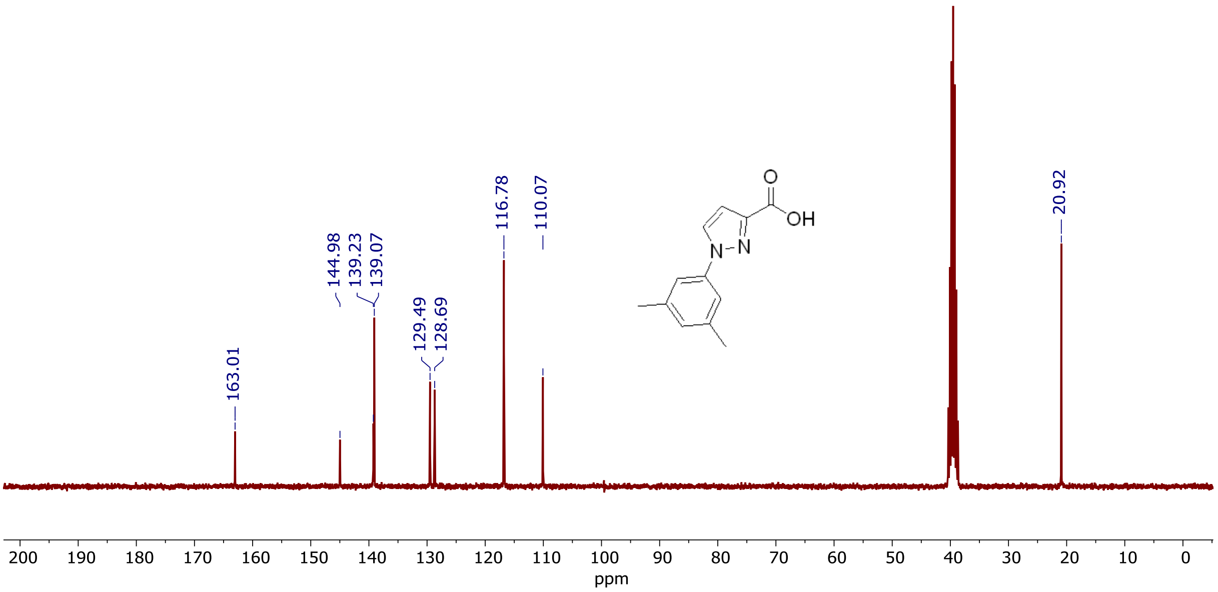

Figure S11: ^{13}C{^{1}H} NMR spectrum (DMSO-d_6, 298 K, 75 MHz) of **1f**.

Synthesis of carboxylic acid 1g

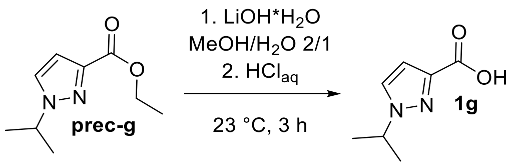

Prec-g (500 mg, 2.75 mmol) was dissolved in a mixture of MeOH/H_2O (2:1 v/v, 5 mL) and lithium hydroxide monohydrate (230 mg, 5.00 mmol) was added. After stirring the mixture for 3 h at rt, it was extracted with CH_2Cl_2 (3 x 20 mL). The organic fractions were combined, dried over $MgSO_4$, filtered, and dried under vacuo to yield **1g** as a colorless solid (405 mg, 95%). The carboxylic acid **1g** was used for the next step without any further purification. NMR data in agreement with those in the literature.[S6]
^{1}H NMR (300 MHz, DMSO-d_6, 298K) δ = 7.85 (d, $^3J_{HH}$ = 2.4 Hz, 1H, CH_{prz}), 6.66 (d, $^3J_{HH}$ = 2.3 Hz, 1H, CH_{prz}), 4.56 (hept, $^3J_{HH}$ = 6.7 Hz, 1H, *i*Pr-C*H*), 1.49 (d, $^3J_{HH}$ = 6.7 Hz, 6H, *i*Pr-CH_3) ppm.

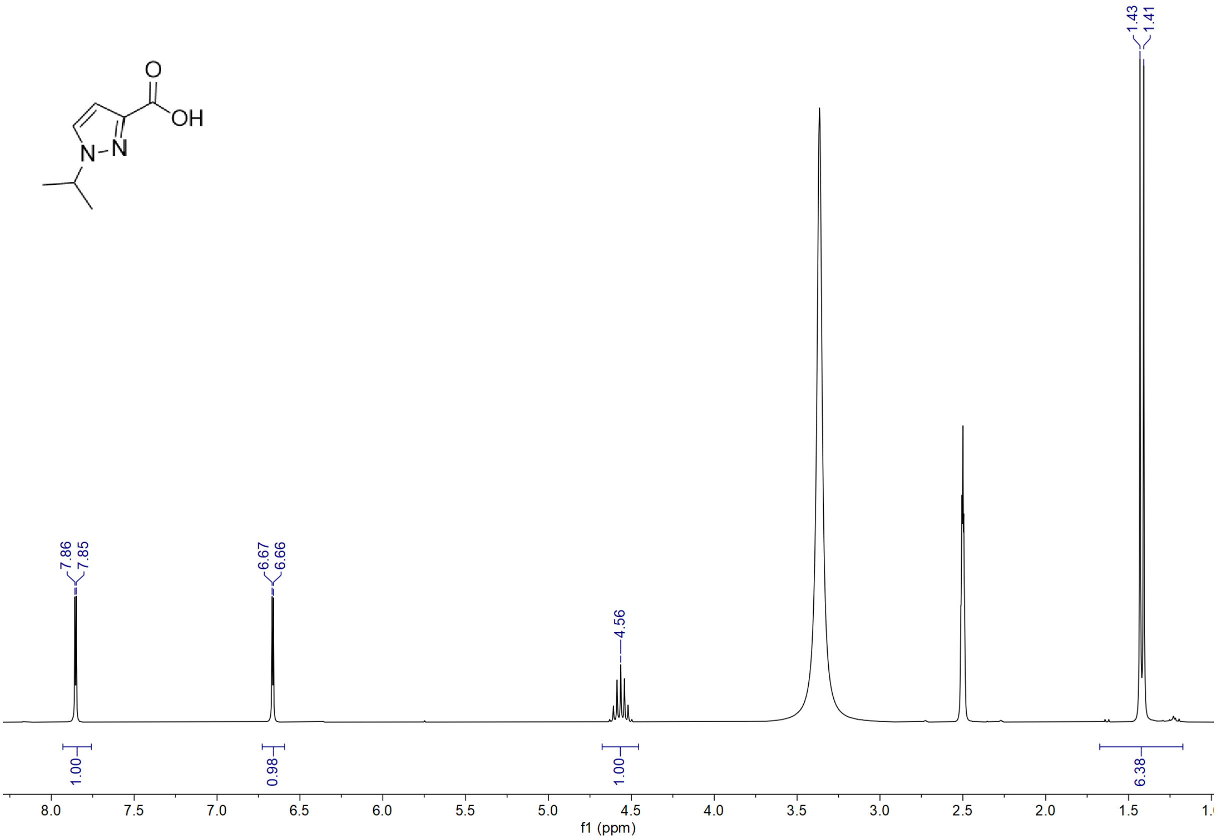

Figure S12: ^{1}H NMR spectrum (DMSO-d_6, 298 K, 300 MHz) of **1g**.

S.1.4 Synthesis of pyridine carboxamides 2a–2f

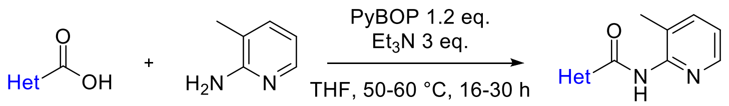

1 **2**

Scheme S1: General procedure for the preparation of pyridine carboxamides **2a–f**.

General procedure I: To a solution of the carboxylic acid (10 mmol, 1 eq.), benzotriazol-1-yloxytripyrrolidinophosphonium hexafluorophosphate (PyBOP; 6.24 g, 12.5 mmol, 1.2 eq.) and the appropriate aminopyridine (10 to 11 mmol, 1 to 1.1 eq.) in dry THF (20 mL) was added Et_3N (4.18 mL, 30 mmol, 3 eq.). The resulting mixture was stirred under the conditions (temperature, time) as specified. After cooling to room temperature, H_2O (40 mL) was added and the mixture was extracted with EtOAc (3 x 20 mL). The combined organic layers were washed with brine (20 mL), dried over $MgSO_4$, filtered, and concentrated under vacuo. The resulting brownish residue was purified by flash column chromatography (SiO_2) to yield **2**.

Synthesis of pyridine carboxamide 2a

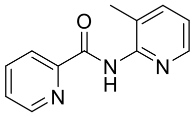

2a

2a was prepared based on the previously reported procedure[S7] from picolinic acid (2.0 g, 16.3 mmol) and 3-methylpyridin-2-amine (1.65 mL, 16.3 mmol) at 50°C for 16 h. The resulting waxy solid was purified by gradient FCC (0 to 100% EtOAc in n-hexane) to yield compound **2a** as an off-white solid (2.77 g, 80%). Analytical data are in accordance with literature.[S7] ^{1}H NMR (300 MHz, DMSO-d_6, 298K) δ = 10.53 (s, 1H, N*H*), 8.74 (ddd, J_{HH} = 4.8, 1.7, 1.0 Hz, 1H, CH_{py}), 8.31 (dd, J_{HH} = 5.0, 1.8 Hz, 1H, CH_{pyr}), 8.14 (d, $^3J_{HH}$ = 7.7 Hz, 1H, CH_{py}), 8.07 (td, J_{HH} = 7.6, 1.7 Hz, 1H, CH_{py}), 7.74 (dd, J_{HH} = 7.6, 1.8 Hz, 1H, CH_{pyr}), 7.69 (ddd, J_{HH} = 7.4, 4.8, 1.4 Hz, 1H, CH_{py}), 7.26 (dd, $^3J_{HH}$ = 7.5, 4.8 Hz, 1H, CH_{pyr}), 2.25 (s, 3H, CH_3) ppm.

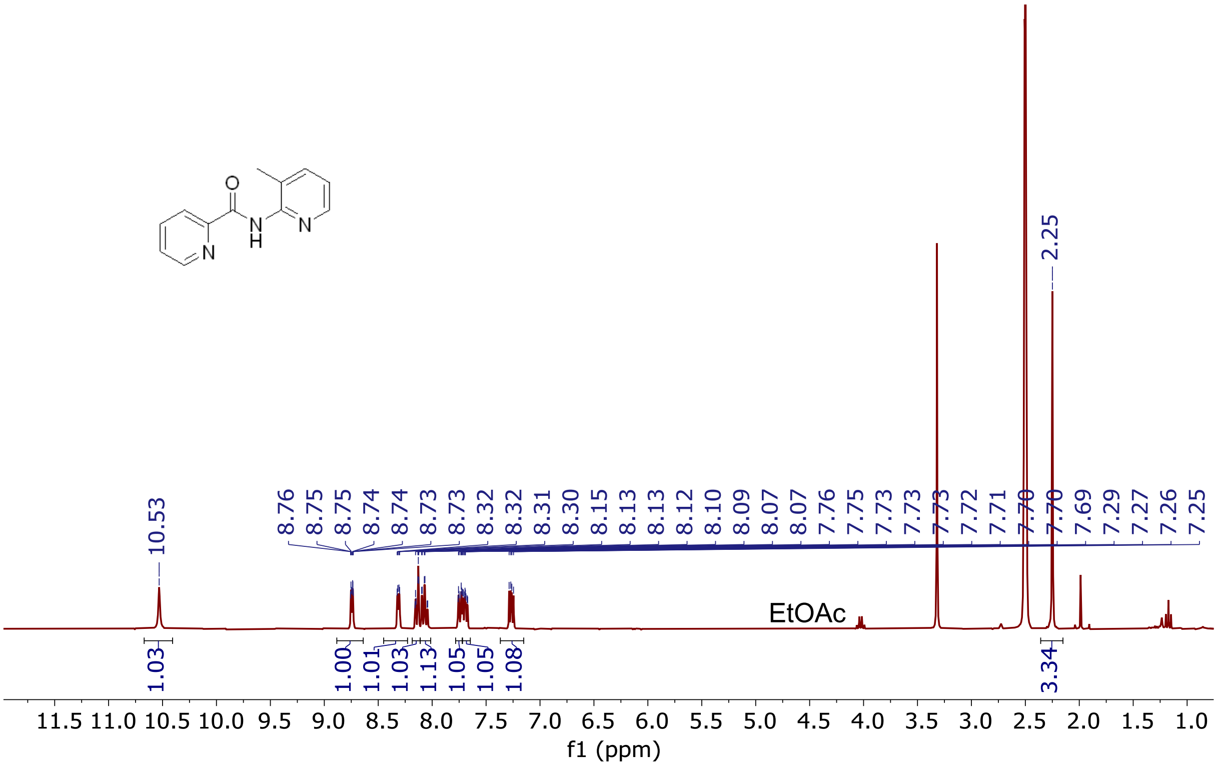

Figure S13: ^{1}H NMR spectrum (DMSO-d_6, 298 K, 300 MHz) of **2a**.

Synthesis of pyridine carboxamide 2b

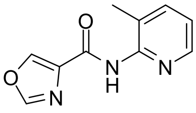

2b

2b was prepared according to the general procedure I from oxazol-4-carboxylic acid (1.13 g, 10 mmol) and 2-amino-3-methylpyridine (1.01 mL, 10 mmol) at 60 °C for 30 h. The residue was purified by gradient FCC (0 to 100% EtOAc in pentane; rf 0.5 in EtOAc) to yield **2b** as an off-white solid (1.36 g, 67%). ^{1}H NMR (400 MHz, DMSO-d_6, 298K) δ = 10.20 (s, 1H, NH), 8.81 (d, $^{4}J_{HH}$ = 1.0 Hz, 1H, CH_{oxa}), 8.60 (d, $^{4}J_{HH}$ = 1.0 Hz, 1H, CH_{oxa}), 8.31 (dd, J_{HH} = 4.8, 1.8 Hz, 1H, CH_{pyr}), 7.73 (dd, J_{HH} = 7.5, 1.8 Hz, 1H, CH_{pyr}), 7.27 (dd, J_{HH} = 7.5, 4.8 Hz, 1H, CH_{pyr}), 2.22 (s, 3H, CH_3) ppm. ^{13}C{^{1}H} NMR (101 MHz, DMSO-d_6, 298K) δ = 159.13 (CO), 152.90 (CH$_{oxa}$), 149.74 (C_{pyr}), 146.48 (CH$_{pyr}$), 143.32 (CH$_{oxa}$), 139.78 (CH$_{pyr}$), 135.67(C_{oxa}), 130.16 (C_{pyr}–Me), 122.84 (CH$_{pyr}$), 17.92 (pyr-CH$_3$) ppm. HR-MS (m/z): calculated for $C_{10}H_{10}N_3O_2$ [M+H]$^+$ = 204.0768; found: 204.0759.

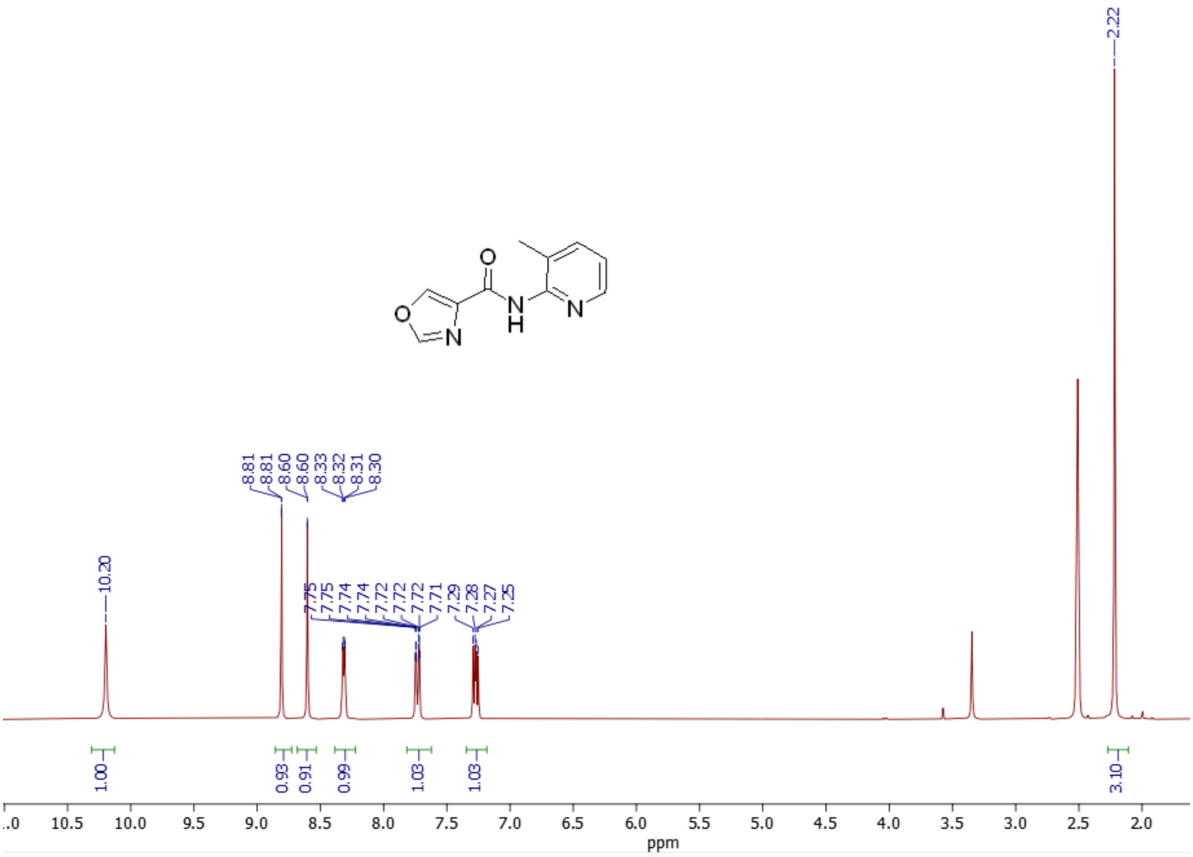

Figure S14: ^{1}H NMR spectrum (DMSO-d_6, 298 K, 400 MHz) of **2b**.

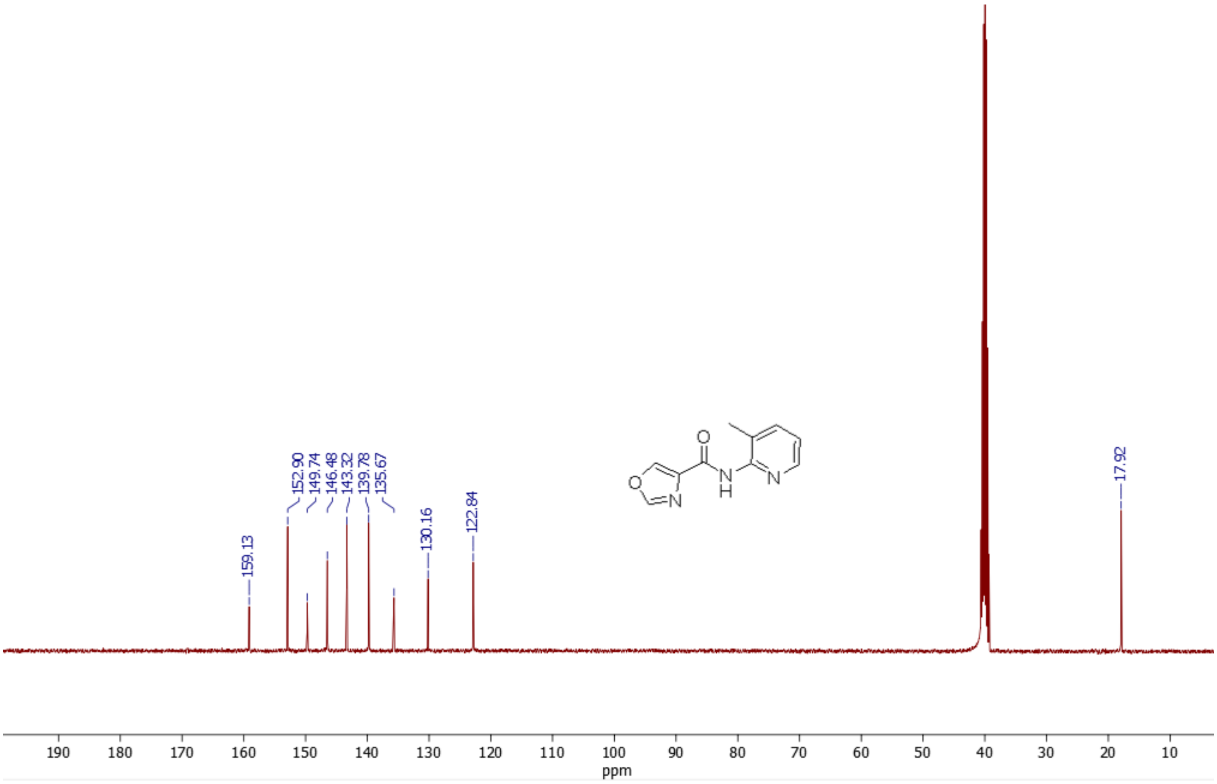

Figure S15: $^{13}C\{^{1}H\}$ NMR spectrum (DMSO-d_6, 298 K, 101 MHz) of **2b**.

Synthesis of pyridine carboxamide 2c

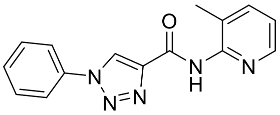

2c

2c was prepared according to the general procedure I from 1-phenyl-1H-1,2,3-triazole-4-carboxylic acid (1.10 g, 5.81 mmol) and 2-amino-3-methylpyridine (650 µL, 6.40 mmol) at 23 °C for 18 h. The residue was purified by gradient FCC (50 to 100% EtOAc in pentane, rf 0.2 in pentane/EtOAc 1:1) to yield **2c** as a white solid (1.25 g, 77%). ^{1}H NMR (400 MHz, DMSO-d_6, 298K) δ = 10.58 (s, 1H, NH), 9.45 (s, 1H, CH_{trz}), 8.34 (d, $^3J_{HH}$ = 4.8 Hz, 1H, CH_{pyr}), 8.01 (d, $^3J_{HH}$ = 7.8 Hz, 2H, CH_{phe}), 7.75 (d, $^3J_{HH}$ = 7.6 Hz, 1H, CH_{pyr}), 7.65 (t, $^3J_{HH}$ = 7.8 Hz, 2H, CH_{phe}), 7.56 (t, $^3J_{HH}$ = 7.8 Hz, 1H, CH_{phe}), 7.29 (dd, $^3J_{HH}$ = 7.6, 4.8 Hz, 1H, CH_{pyr}), 2.27 (s, 3H, CH_3) ppm. ^{13}C{^{1}H} NMR (101 MHz, DMSO-d_6, 298K) δ = 158.73 (CO), 149.88 (C_{pyr}), 146.54 (CH$_{pyr}$), 143.69 (C_{trz}), 139.80 (CH$_{pyr}$), 136.77 (C_{phe}), 130.44 (CH$_{phe}$), 130.25 (C_{pyr}-Me), 129.73(CH$_{phe}$), 126.03 (CH$_{trz}$), 122.87 (CH$_{pyr}$), 121.05 (CH$_{phe}$), 17.98 (CH$_3$) ppm. HR-MS (m/z): calcd. for $C_{15}H_{14}N_5O$ $[M+H]^+$ = 280.1193; found: 280.1182.

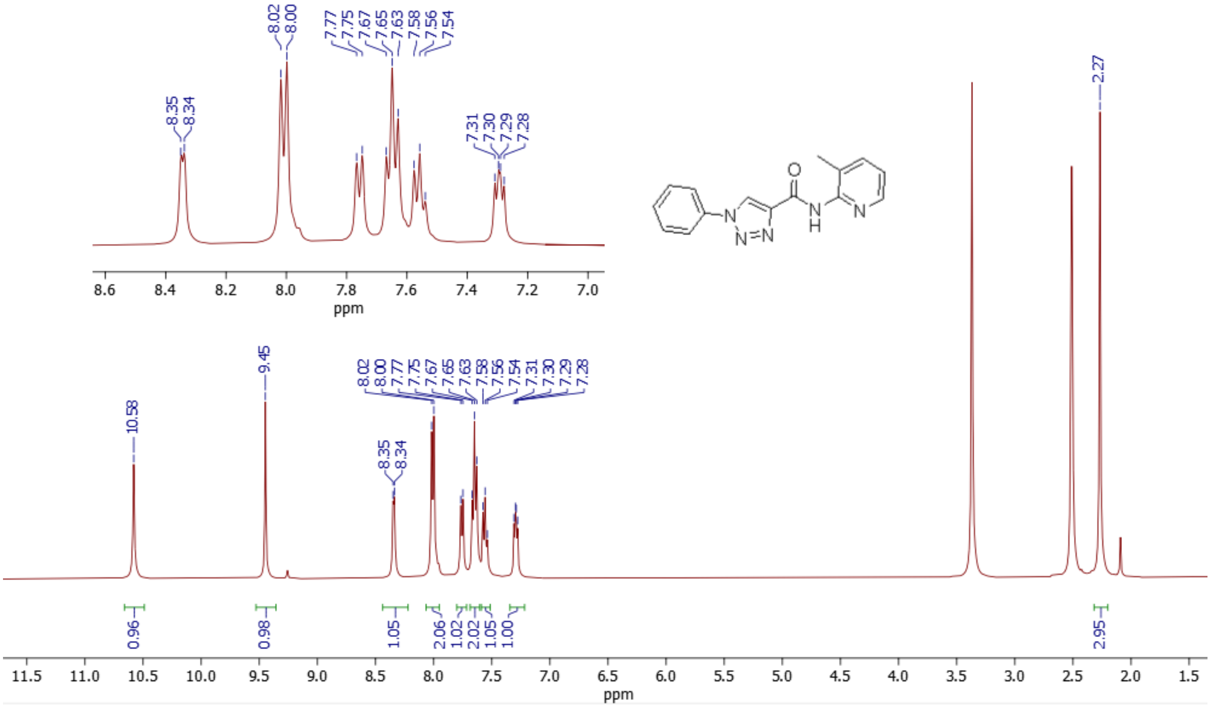

Figure S16: ^{1}H NMR spectrum (DMSO-d_6, 298 K, 400 MHz) of **2c**

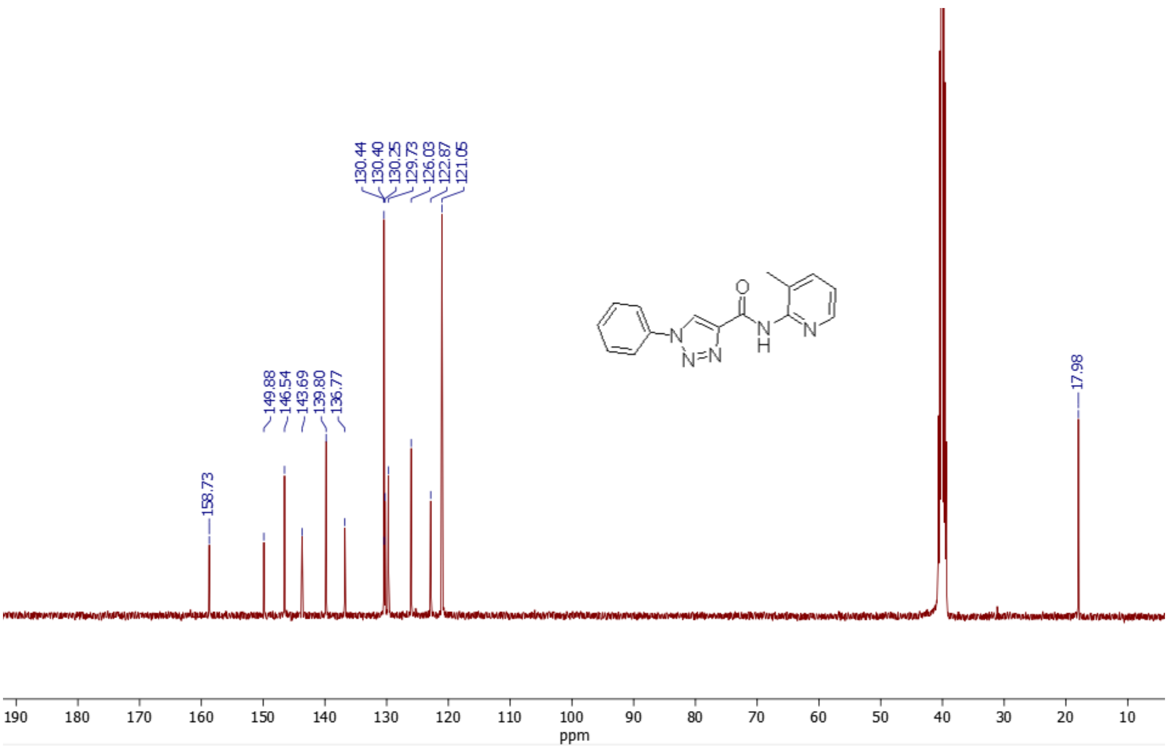

Figure S17: $^{13}C\{^{1}H\}$ NMR spectrum (DMSO-d_6, 298 K, 101 MHz) of **2c**

Synthesis of pyridine carboxamide 2d

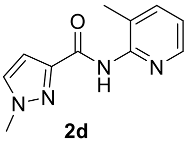

2d

2d was prepared according to the general procedure I from 1-methyl-1H-pyrazole-3-carboxylic acid (950 mg, 10 mmol) and 2-amino-3-methylpyridine (1 mL, 10 mmol) at 60 °C for 30 h. The resulting waxy solid was purified by gradient FCC (0 to 8% MeOH in EtOAc; 3rd fraction) to yield compound **2d** as a pale-yellow waxy solid (1.59 g, 62%). ^{1}H NMR (400 MHz, DMSO-d_6, 298 K): δ = 9.91 (s, 1H, N*H*), 8.29 (dd, J_{HH} = 4.8, 1.8 Hz, 1H, CH_{pyr}, 7.85 (d, $^{3}J_{HH}$ = 2.3 Hz, 1H, C*H*), 7.71 (dd, J_{HH} = 7.5, 1.8 Hz, 1H, CH_{pyr}), 7.24 (dd, $^{3}J_{HH}$ = 7.5, 4.8 Hz, 1H, CH_{pyr}), 6.78 (d, $^{3}J_{HH}$ = 2.3 Hz, 1H, CH_{prz}), 3.96 (s, 3H, NCH_3), 2.21 (s, 3H, pyr-CH_3) ppm. $^{13}C\{^{1}H\}$ NMR (101 MHz, DMSO-d_6, 298K) δ = 159.85 (*C*O), 149.77 (C_{pyr}), 145.86 (*C*H_{pyr}), 145.62 (C_{prz}), 139.11 (*C*H_{pyr}), 132.79 (*C*H_{prz}), 129.50 (C_{pyr}-Me), 121.97 (*C*H_{pyr}), 106.55 (*C*H_{prz}), 39.02 (N-*C*H_3), 17.56 (pyr-*C*H_3) ppm. HR-MS (m/z): calculated for $C_{11}H_{13}N_4O$ $[M+H]^+$ = 217.1084; found: 217.1078.

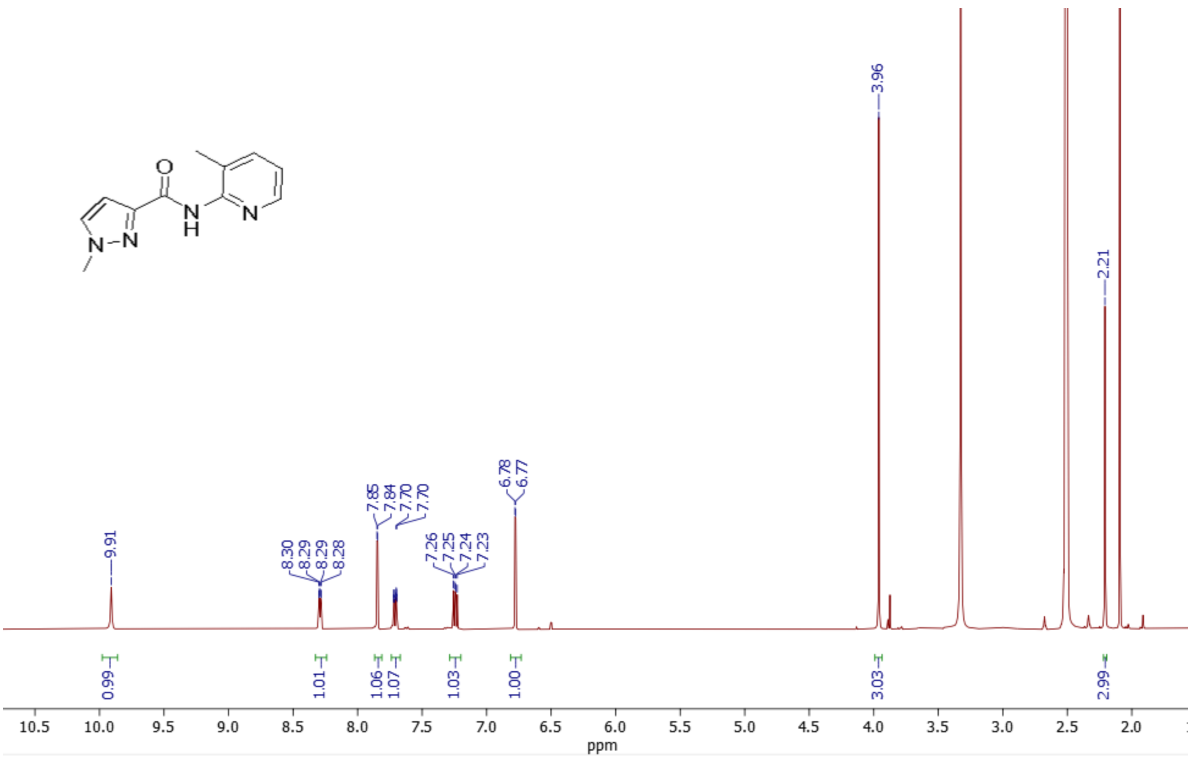

Figure S18: ^{1}H NMR spectrum (DMSO-d_6, 298 K, 400 MHz) of **2d**

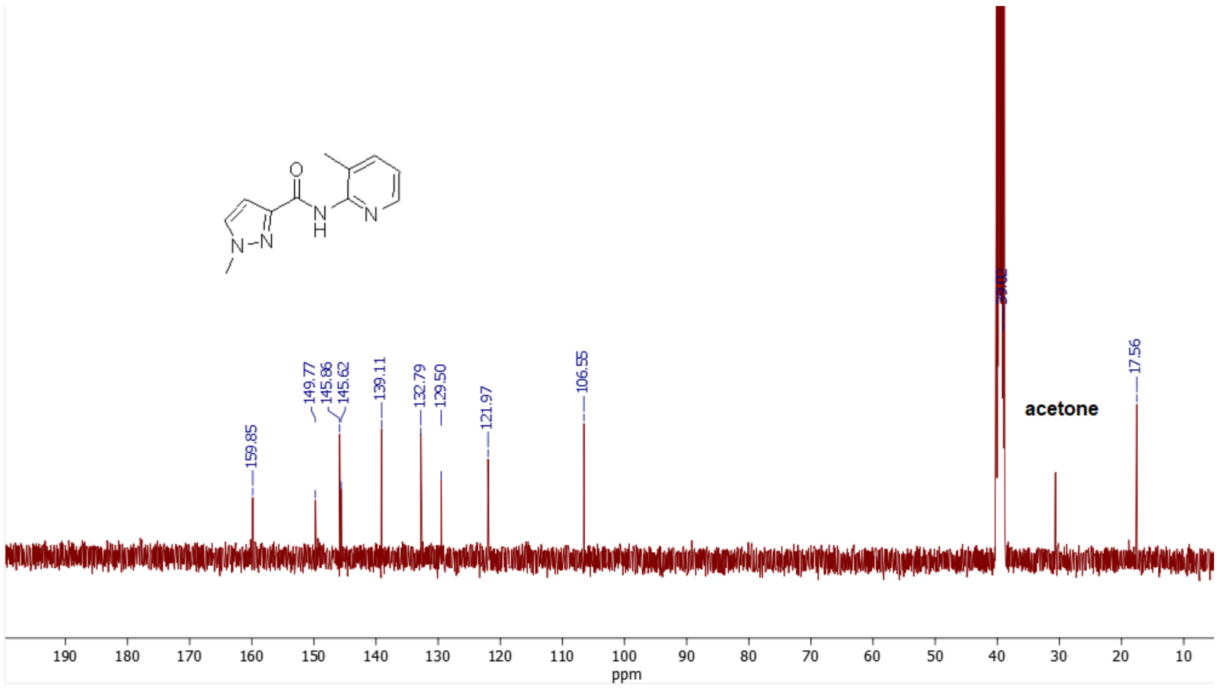

Figure S19: $^{13}C\{^{1}H\}$ NMR spectrum (DMSO-d_6, 298 K, 101 MHz) of **2d**

Synthesis of pyridine carboxamide 2e

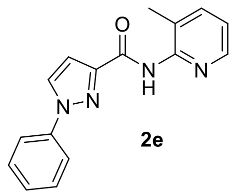

2e was prepared according to the general procedure I from **1e** (1.50 g, 8.0 mmol) and 2-amino-3-methylpyridine (890 µL, 8.8 mmol) at 45 °C for 30 h. The residue was purified by FCC (0 to 100% EtOAc in pentane) to yield **2e** as a yellow solid (1.69 g, 76%). ^{1}H NMR (400 MHz, DMSO-d_6, 298K) δ = 10.29 (s, 1H, NH), 8.67 (d, $^3J_{HH}$ = 2.6 Hz, 1H, CH_{prz}), 8.34 (dd, J_{HH} = 4.8, 1.9 Hz, 1H, CH_{pyr}), 8.08 – 7.97 (m, 2H, CH_{phe}), 7.75 (dd, J_{HH} = 7.5, 1.9 Hz, 1H, CH_{pyr}), 7.63 – 7.49 (m, 2H, CH_{phe}), 7.46 – 7.35 (m, 1H, CH_{phe}), 7.28 (dd, $^3J_{HH}$ = 7.5, 4.8 Hz, 1H, CH_{pyr}), 7.06 (d, $^3J_{HH}$ = 2.6 Hz, 1H, CH_{prz}), 2.25 (s, 3H, pyridine-CH_3) ppm. ^{13}C{^{1}H} NMR (101 MHz, DMSO-d_6, 298K) δ = 159.65 (CO), 149.65(C_{pyr}), 147.21 (C_{prz}), 145.94 (CH$_{\text{pyr}}$), 139.26 (CH$_{\text{pyr}}$), 139.23 (C_{phe}), 129.74 (CH$_{\text{prz}}$), 129.67 (C_{pyr}-Me), 129.55 (CH$_{\text{phe}}$) 127.16 (CH$_{\text{phe}}$), 122.16 (CH$_{\text{pyr}}$), 119.00 (CH$_{\text{phe}}$), 108.45 (CH$_{\text{prz}}$), 17.61 (CH$_3$) ppm. HR-MS (m/z): calculated for $C_{16}H_{15}N_4O$ [M+H]$^+$ = 279.1241; found: 279.1228.

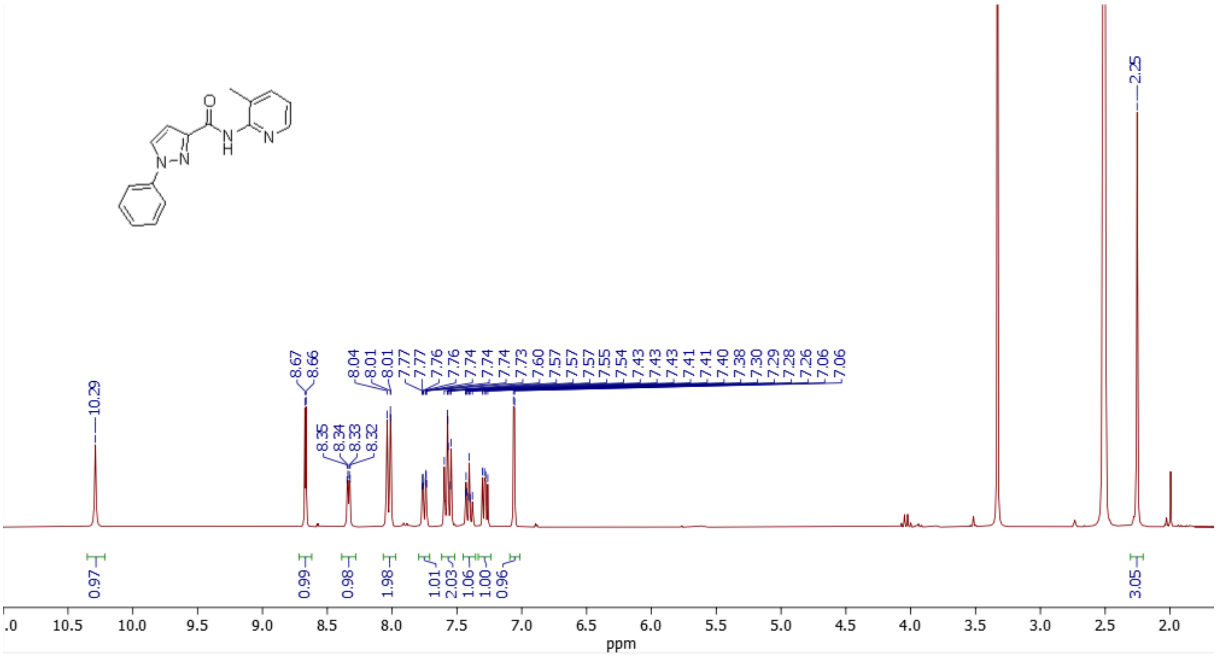

Figure S20: ^{1}H NMR spectrum (DMSO-d_6, 298 K, 400 MHz) of **2e**

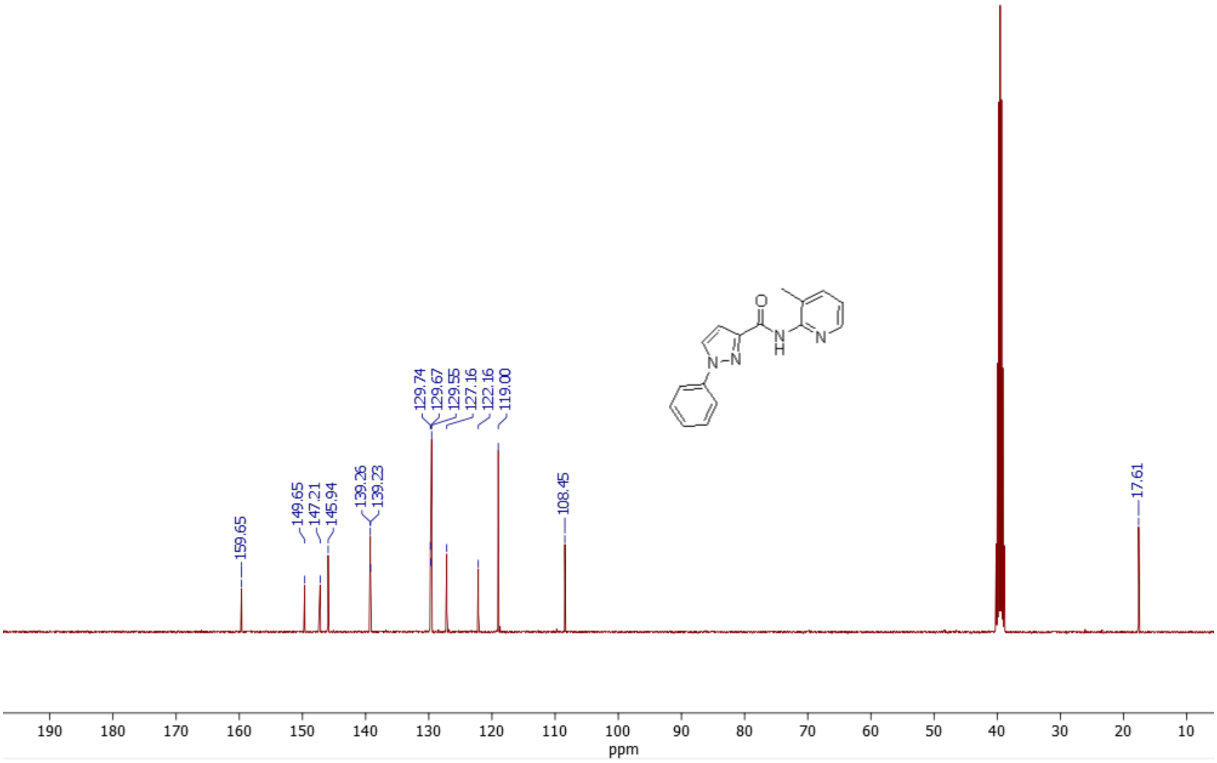

Figure S21: $^{13}C\{^{1}H\}$ NMR spectrum (DMSO-d_6, 298 K, 101 MHz) of **2e**

Synthesis of pyridine carboxamide 2f

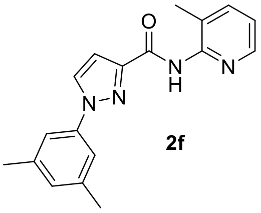

2f

2f was prepared according to the general procedure I from **1f** (2.00 g, 9.25 mmol) and 2-amino-3-methylpyridine (1.00 mL, 9,88 mmol) at 45 °C for 18 h. The residue was purified by gradient FCC (20 to 50% EtOAc in pentane, rf 0.7 in EtOAc/pentane 1:1) to yield **2f** as a whitish solid (1.90 g, 67%). ^{1}H NMR (400 MHz, DMSO-d_6, 298K) δ = 10.28 (s, 1H, N*H*), 8.60 (d, $^{3}J_{HH}$ = 2.6 Hz, 1H, CH_{prz}), 8.33 (dd, J_{HH} = 4.8, 1.8 Hz, 1H, CH_{pyr}), 7.75 (dd, J_{HH} = 7.5, 1.8 , 1H, CH_{pyr}), 7.66 (s, 2H, CH_{phe}), 7.28 (dd, $^{3}J_{HH}$= 7.5, 4.8 Hz, 1H, CH_{pyr}), 7.04 (m, 1H, CH_{phe}), 7.02 (d, $^{3}J_{HH}$ = 2.6 Hz, 1H, CH_{prz}), 2.37 (s, 6H, phe-CH_3), 2.25 (s, 3H, pyr-CH_3) ppm. $^{13}C\{^{1}H\}$ NMR (101 MHz, DMSO-d_6, 298K) δ = 159.69 (*C*O), 149.67 (C_{pyr}), 146.94 (C_{prz}), 145.90 (CH_{pyr}), 139.24 (CH_{pyr}), 139.16 (C_{phe}), 138.90 (C_{phe}-Me) 129.71 (C_{pyr}-Me), 129.56 (CH_{prz}), 128.47(CH_{phe}), 122.14 (CH_{pyr}), 116.66 (CH_{phe}), 108.24 (CH_{prz}), 20.89 (phe-CH_3), 17.62(pyr-CH_3) ppm. HR-MS (m/z): calculated for $C_{18}H_{19}N_4O$ $[M+H]^+$ = 307.1554; found: 307.1541.

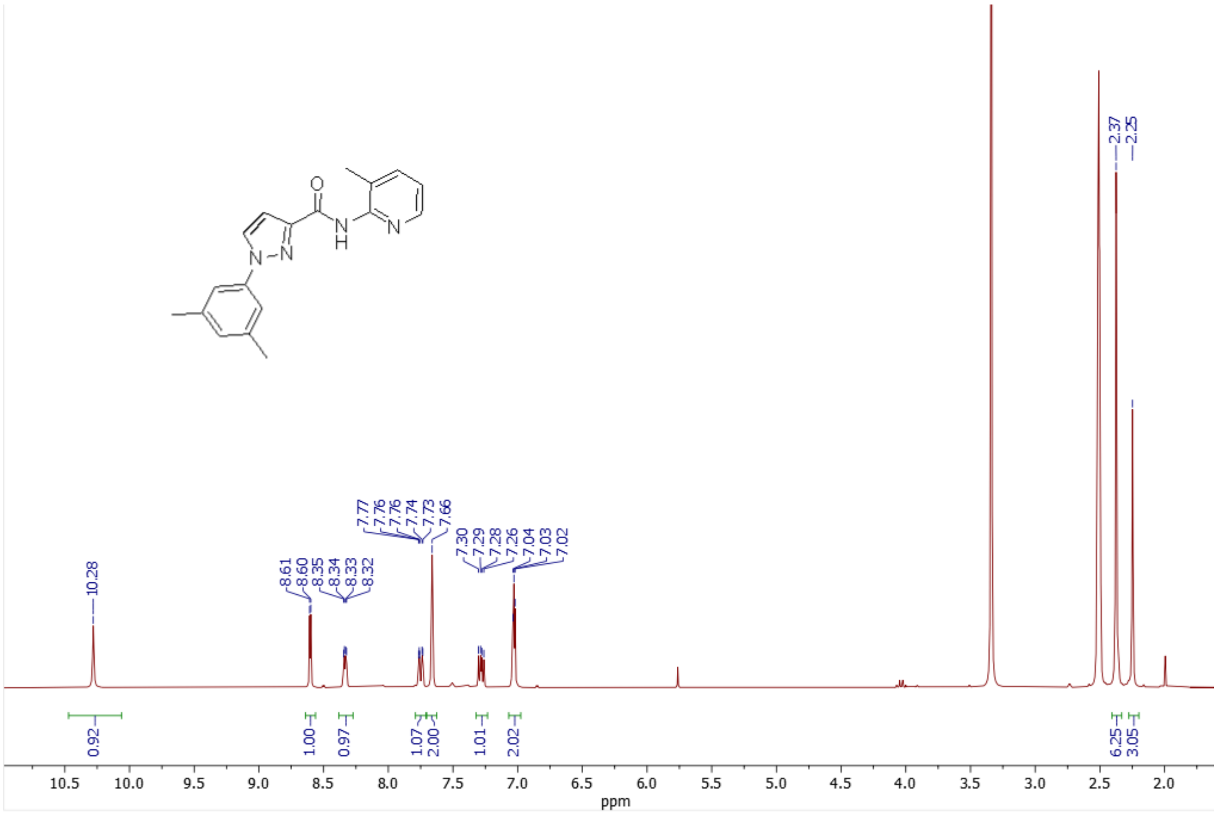

Figure S22: ^{1}H NMR spectrum (DMSO-d_6, 298 K, 400 MHz) of **2f**

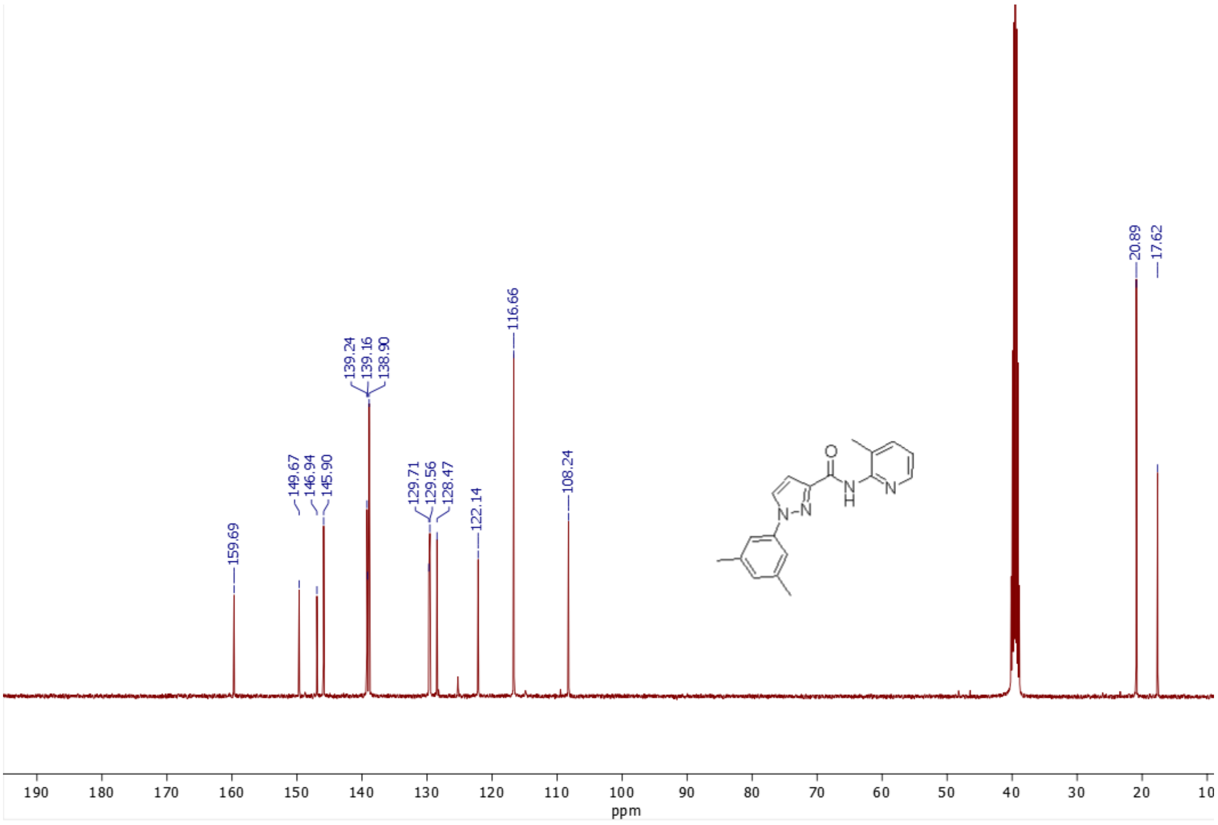

Figure S23: ^{13}C{^{1}H} NMR spectrum (DMSO-d_6, 298 K, 101 MHz) of **2f**

S.1.5 Synthesis of pyridinium iodide salts 3a–3g

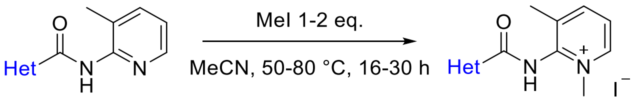

2 **3**

Scheme S2: General procedure for the preparation pyridinium iodide salts **3a–g**.

General procedure II: To a solution of the pyridine carboxamide **2** (5.0 mmol, 1 eq.) in MeCN (15 mL) was added MeI (620 µL, 10.0 mmol, 2 eq.). The resulting mixture was stirred under the conditions (temperature, time) specified, then concentrated to 5 mL, and the product was precipitated by addition of Et_2O. The precipitate was collected by filtration, washed with cold Et_2O, and dried in vacuo to give the title product **3**.

Synthesis of 3a

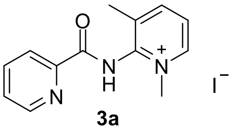

3a

3a has been reported previously.[S7] According to the general procedure II from **2a** (2.0 g, 9.4 mmol) and MeI (1.2 mL, 18.8 mmol) at 60 °C for 16 h, salt **3a** was obtained as a yellow solid (3.1 g, 93%). Analytical data are in agreement with literature values.[S7] ^{1}H NMR (300 MHz, CD_3CN, 298K) δ = 10.43 (s, 1H, NH), 8.79 – 8.71 (m, 1H, CH_{py}), 8.59 (d, $^3J_{HH}$ = 6.1 Hz, 1H, CH_{pyr}), 8.44 (d, $^3J_{HH}$ = 7.9 Hz, 1H, CH_{pyr}), 8.26 – 8.17 (m, 1H, CH_{py}), 8.08 (td, J_{HH} = 7.9, 1.7 Hz, 1H, CH_{py}), 7.86 (dd, $^3J_{HH}$ = 8.0, 6.2 Hz, 1H, CH_{pyr}), 7.72 (ddd, J_{HH} = 7.6, 4.8, 1.3 Hz, 1H, CH_{py}), 4.22 (s, 3H NCH_3), 2.46 (s, 3H, pyrCH_3) ppm.

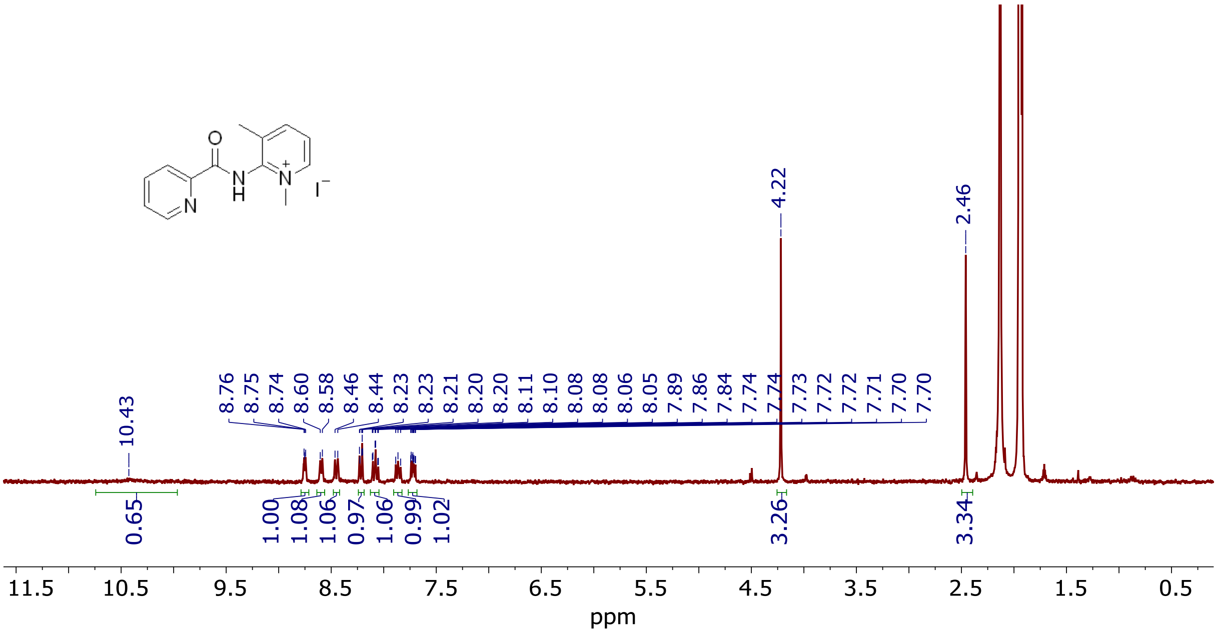

Figure S24: ^{1}H NMR spectrum (CD_3CN, 298 K, 300 MHz) of **3a**

Synthesis of 3b

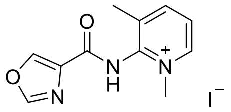

3b

According to the general procedure II from **2b** (1.02 g, 5.0 mmol) at 70°C for 16 h, salt **3b** was obtained as a yellow solid (1.35 g, 78%). ^{1}H NMR (400 MHz, CD_3CN, 298K) δ = 9.66 (s, 1H, NH), 8.66 (d, $^{3}J_{HH}$ = 6.2 Hz, 1H, CH_{pyr}), 8.64 (d, $^{4}J_{HH}$ = 1.0 Hz, 1H, CH_{oxa}), 8.46 (d, $^{3}J_{HH}$ = 8.0 Hz, 1H, CH_{pyr}), 8.25 (d, $^{4}J_{HH}$ = 1.0 Hz, 1H, CH_{oxa}), 7.88 (dd, $^{3}J_{HH}$ = 8.0, 6.2 Hz, 1H, CH_{pyr}), 4.24 (s, 3H NCH_3), 2.46 (s, 3H, pyrCH_3) ppm. ^{13}C{^{1}H} NMR (101 MHz, CD_3CN, 298K) δ = 159.94 (CO), 153.22 ($C$$H_{oxa}$), 149.40 ($C$$H_{pyr}$), 146.55 ($C_{pyr}$) 145.48 ($C$$H_{pyr}$), 145.01 ($C$$H_{oxa}$), 139.55 ($C_{pyr}$-Me), 134.89 ($C_{oxa}$), 126.74 ($C$$H_{pyr}$), 46.53 (N$C$$H_3$), 18.29 (pyr$C$$H_3$) ppm. HR-MS (m/z): calculated for $C_{11}H_{12}N_3O_2$ $[M-I]^+$ = 218.0925 ; found: 218.0929.

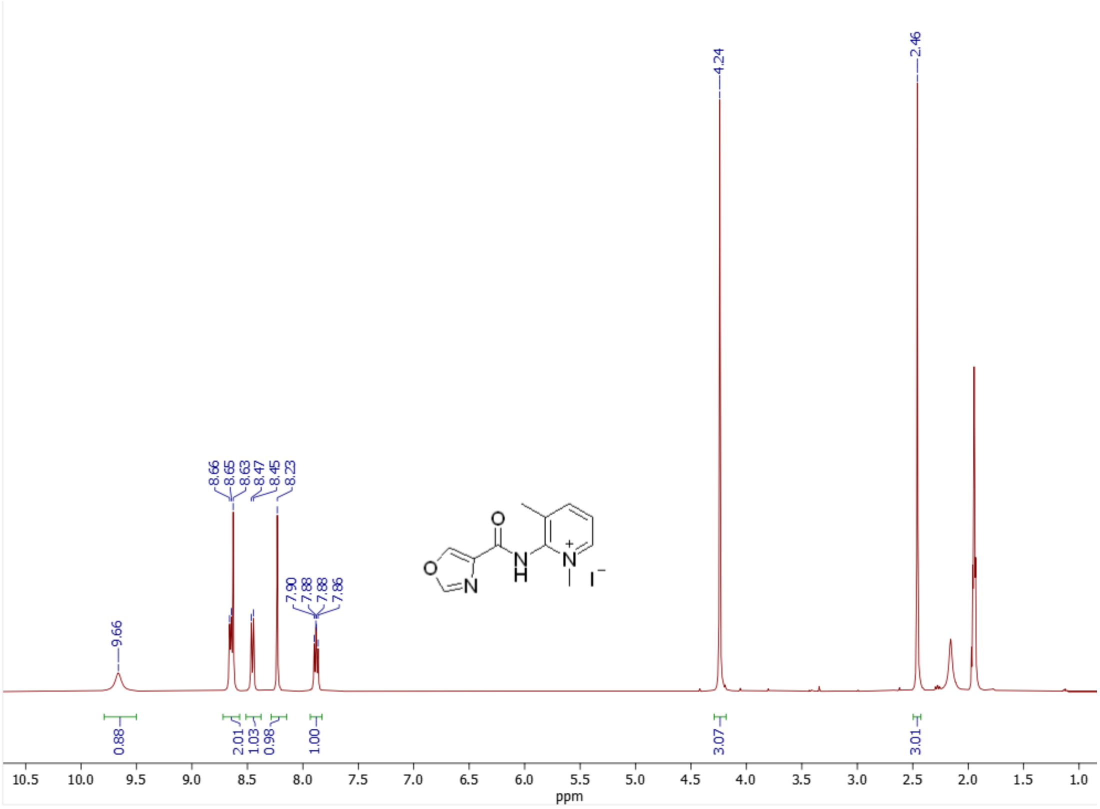

Figure S25: ^{1}H NMR spectrum (CD_3CN, 298 K, 400 MHz) of **3b**

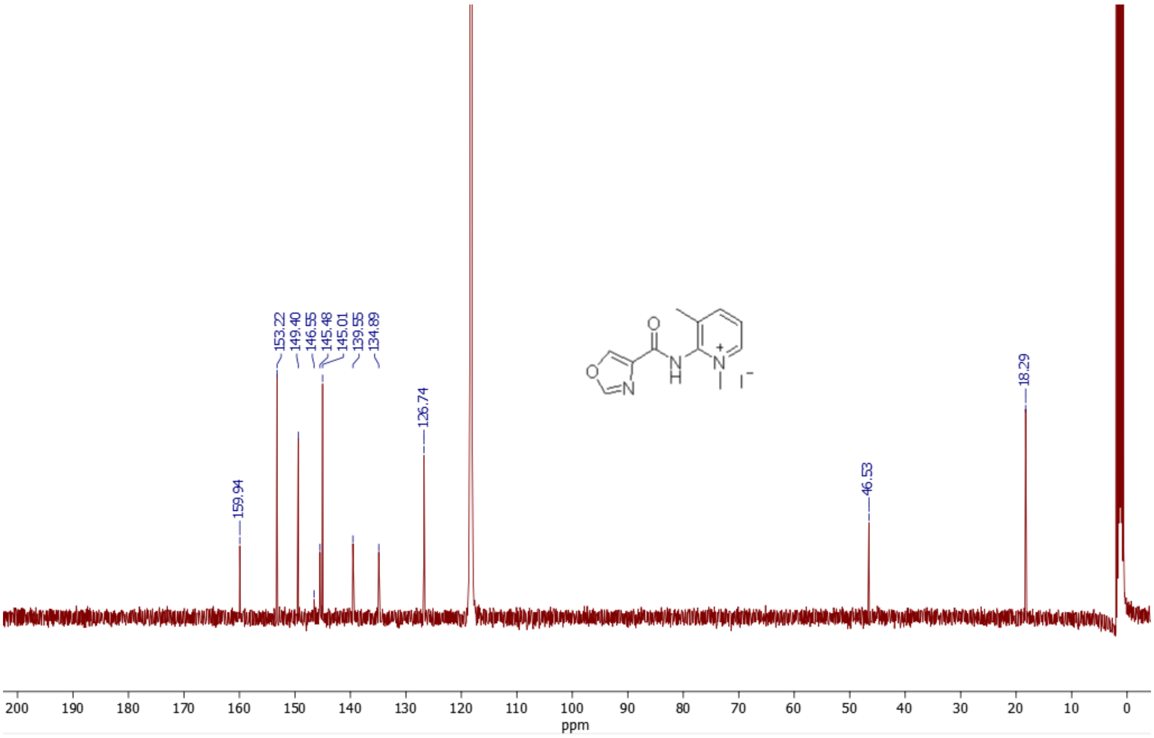

Figure S26: $^{13}C\{^1H\}$ NMR spectrum (CD_3CN, 298 K, 101 MHz) of **3b**

Synthesis of 3c

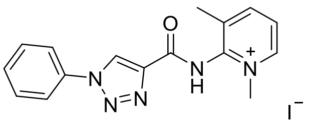

3c

According to the general procedure II from **2c** (1.12 g, 4.0 mmol) at 60 °C for 30 h , salt **3c** was obtained as an off-white solid (1.01 g, 60%). ^1H NMR (300 MHz, CD_3CN, 298K) δ = 9.95 (s, 1H, N*H*), 9.08 (s, 1H, CH_{trz}) 8.67 (d, $^3J_{HH}$ = 6.3 Hz, 1H, CH_{pyr}), 8.49 (d, $^3J_{HH}$ = 8.0 Hz, 1H, CH_{pyr}), 7.96 - 7.85 (m, 3H, CH_{phenyl} + 1 CH_{pyr}) 7.77 - 7.52 (m, 3H, CH_{phe}), 4.30 (s, 3H, NCH_3), 2.51 (s, 3H, pyrCH_3) ppm. $^{13}C\{^1H\}$ NMR (101 MHz, CD_2Cl_2, 298K) δ = 159.41 (*C*O), 149.61 (*C*H_{pyr}), 146.27 (C_{pyr}) 145.66 (*C*H_{pyr}), 142.22 (C_{trz}), 139.73 (C_{pyr}-Me), 137.44 (C_{phe}), 131.00 (*C*H_{phe}), 130.78 (*C*H_{phe}), 127.30 (*C*H_{trz}), 126.98 (*C*H_{pyr}), 122.07 (*C*H_{phe}), 46.65 (N*C*H_3), 18.34 (pyr*C*H_3) ppm. HR-MS (m/z): calculated for $C_{16}H_{16}N_5O$ $[M-I]^+$ = 294.1350 ; found: 294.1347.

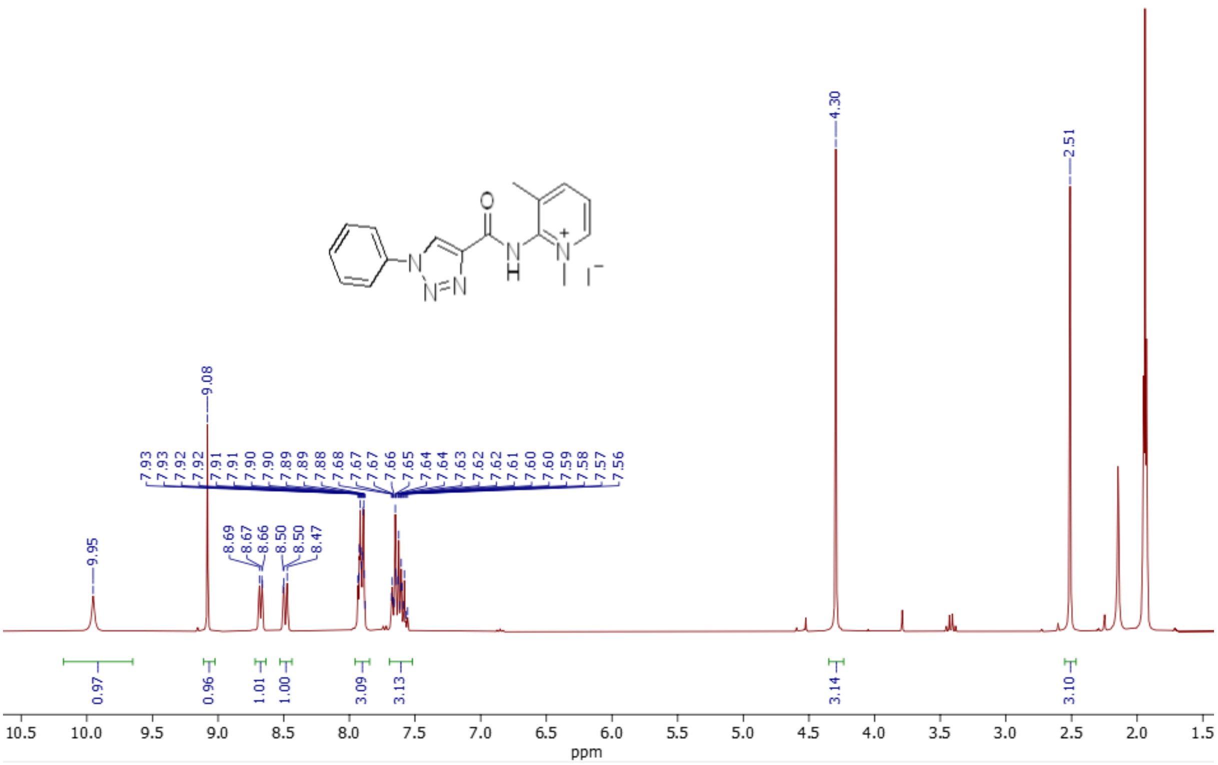

Figure S27: ^{1}H NMR spectrum (CD_3CN, 298 K, 300 MHz) of **3c**

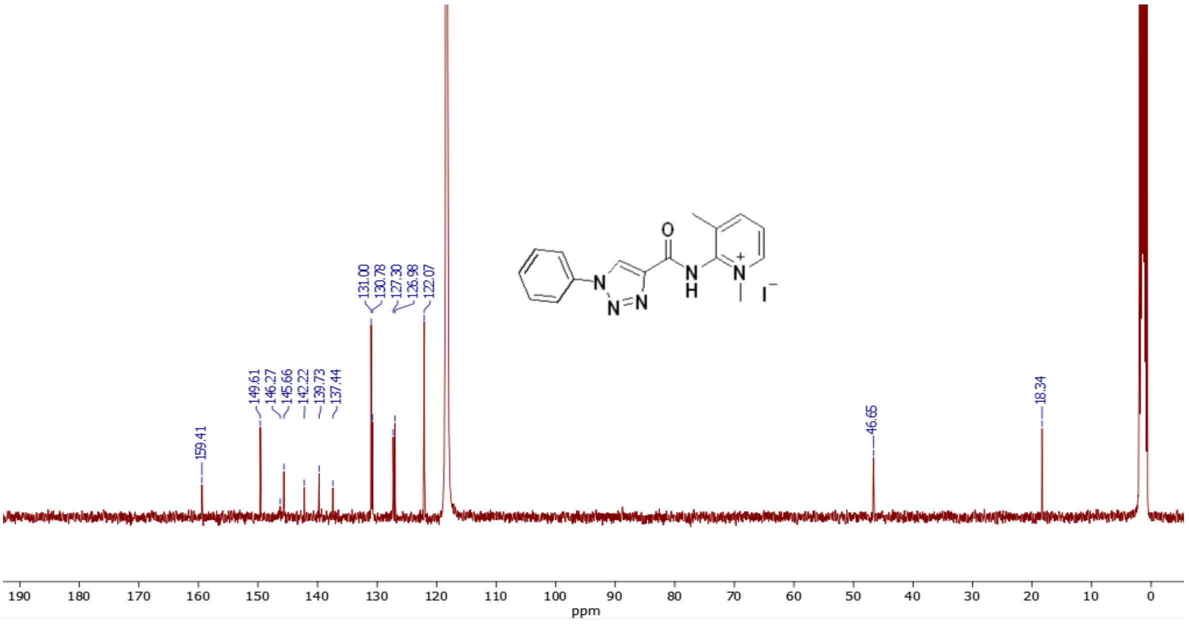

Figure S28: $^{13}C\{^{1}H\}$ NMR spectrum (CD_3CN, 298 K, 101 MHz) of **3c**

Synthesis of 3d

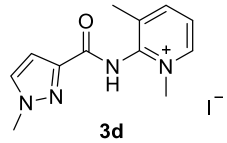

3d

According to the general procedure II from **2d** (1.08 g, 5.0 mmol) at 60 °C for 16 h, salt **3d** was obtained as a yellow solid (1.25 g, 70%). ^{1}H NMR (300 MHz, CD_3CN, 298K) δ = 9.74 (s, 1H, N*H*), 8.78 (dd, J_{HH} = 6.2, 1.6 Hz, 1H, CH_{pyr}), 8.47 (dd, J_{HH} = 8.0, 1.6 Hz, 1H, CH_{pyr}) 7.89 (dd, $^{3}J_{HH}$ = 8.0, 6.2 Hz, 1H, CH_{pyr}), 7.71 (d, $^{3}J_{HH}$ = 2.4 Hz, 1H, CH_{prz}), 6.91 (d, $^{3}J_{HH}$ = 2.4 Hz, 1H, CH_{prz}), 4.26 (s, 3H, pyr-NCH_3), 3.97 (s, 3H, prz-NCH_3), 2.44 (s, 3H, pyr-CH_3) ppm. ^{13}C{^{1}H} NMR (75 MHz, CD_3CN, 298K) δ = 160.98 (*C*O), 148.99 (*C*H_{pyr}), 146.91 (C_{pyr}), 145.13 (*C*H_{pyr}), 144.34 (C_{prz}), 139.43 (C_{pyr}-Me), 134.21 (*C*H_{prz}), 126.48 (*C*H_{pyr}),108.25 (*C*H_{prz}), 46.51 (pyr-N*C*H_3), 40.20 (prz-N*C*H_3), 18.25 (pyr-*C*H_3) ppm. HR-MS (m/z): calculated for $C_{12}H_{15}N_4O$ $[M-I]^{+}$ = 231.1241 ; found: 231.1240.

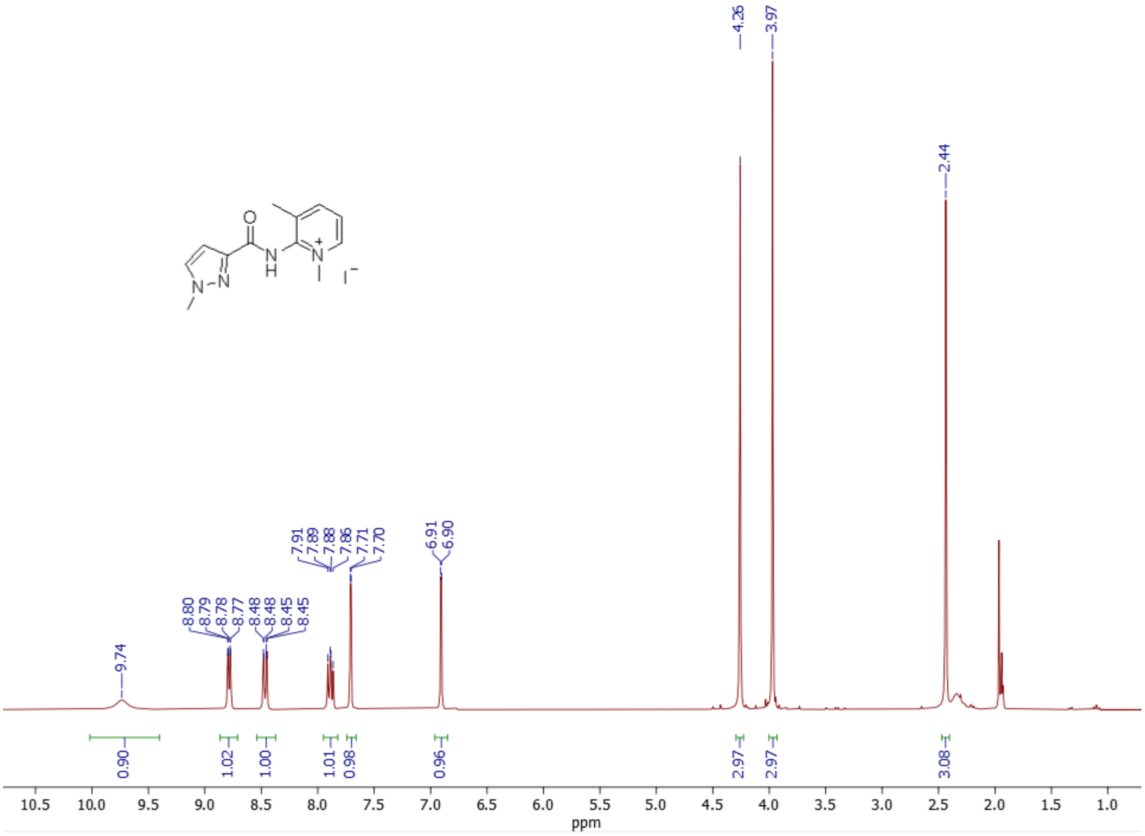

Figure S29: ^{1}H NMR spectrum (CD_3CN, 298 K, 300 MHz) of **3d**

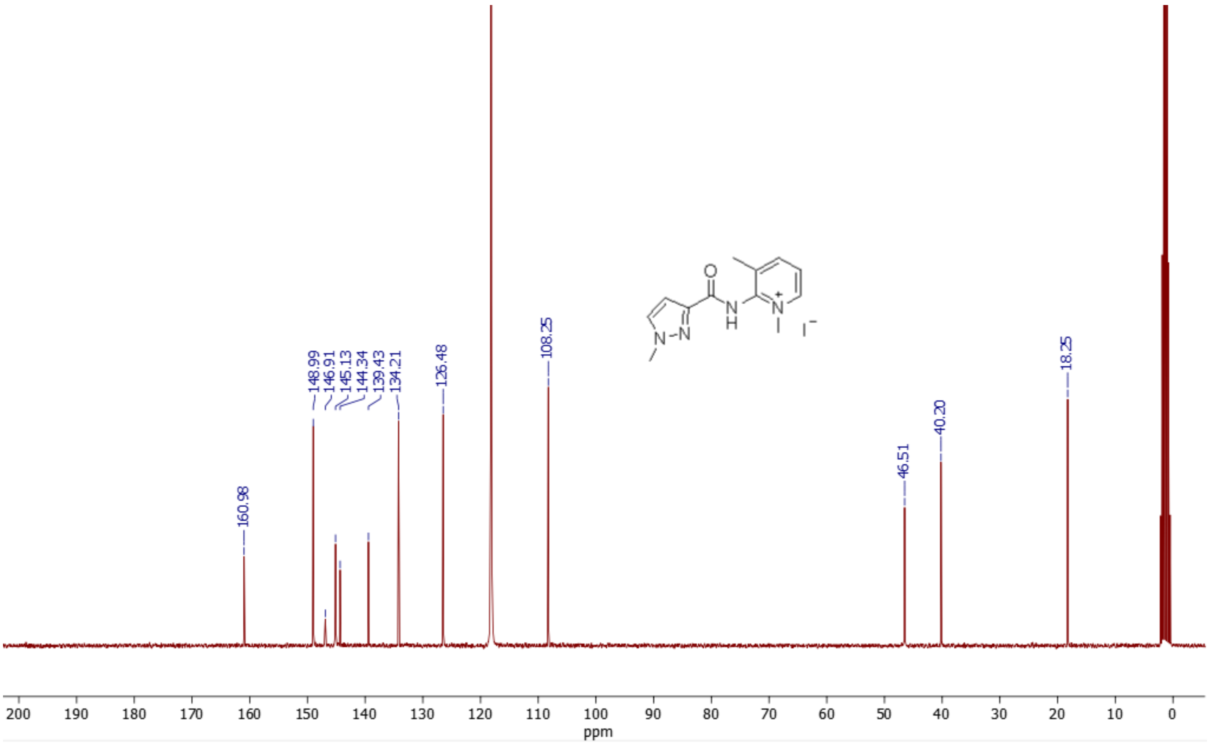

Figure S30: $^{13}C\{^{1}H\}$ NMR spectrum (CD_3CN, 298 K, 75 MHz) of **3d**

Synthesis of 3e

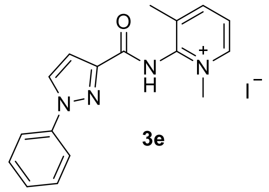

3e

According to the general procedure II from **2e** (1.11 g, 4.0 mmol) and MeI (275 μL, 4.4 mmol) at 60 °C for 16 h, salt **3e** was obtained as a pale yellow solid (1.29 g, 77%). ^{1}H NMR (400 MHz, CD_3CN, 298K) δ = 9.93 (s, 1H, N*H*), 8.66 (d, $^{3}J_{HH}$ = 6.2 Hz, 1H, CH_{pyr}), 8.46 (d, $^{3}J_{HH}$ = 8.0 Hz, 1H, CH_{pyr}), 8.34 (d, $^{3}J_{HH}$ = 2.6 Hz, 1H, CH_{prz}), 7.97 (d, $^{3}J_{HH}$ = 8.1 Hz, 2H, CH_{phe}), 7.88 (dd, $^{3}J_{HH}$ = 8.0, 6.2 Hz, 1H, CH_{pyr}), 7.58 (t, $^{3}J_{HH}$ = 8.1 Hz, 2H, CH_{phe}), 7.45 (t, $^{3}J_{HH}$ = 8.1 Hz, 1H, CH_{phe}), 7.11 (d, $^{3}J_{HH}$ = 2.6 Hz, 1H, CH_{prz}), 4.28 (s, 3H, NCH_3), 2.50 (s, 3H, pyr-CH_3) ppm. $^{13}C\{^{1}H\}$ NMR (101 MHz, CD_3CN, 298K) δ = 161.02 (*C*O), 149.40 (CH_{pyr}), 146.85 (C_{pyr}), 146.22 (C_{prz}), 145.43 (CH_{pyr}), 140.33 (C_{phe}), 139.59 (C_{pyr}-Me), 131.27 (CH_{prz}), 130.73 (CH_{phe}) 128.86 (CH_{phe}) 126.69 (CH_{pyr}), 120.50 (CH_{phe}) 109.85 (CH_{prz}), 46.62 (pyr-NCH_3), 18.35 (pyr-CH_3) ppm. HR-MS (m/z): calculated for $C_{17}H_{17}N_4O$ $[M-I]^+$ = 293.1397 ; found: 293.1395

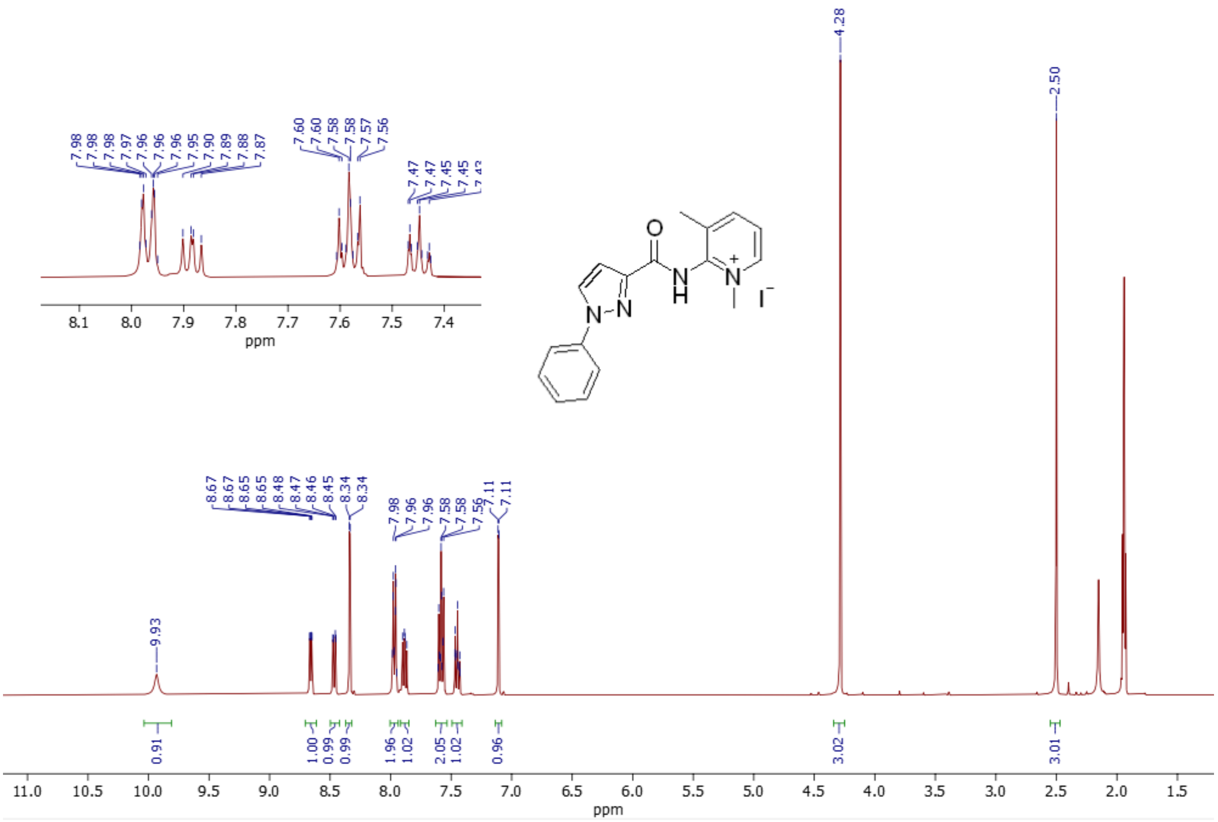

Figure S31: ^{1}H NMR spectrum (CD_3CN, 298 K, 400 MHz) of **3e**

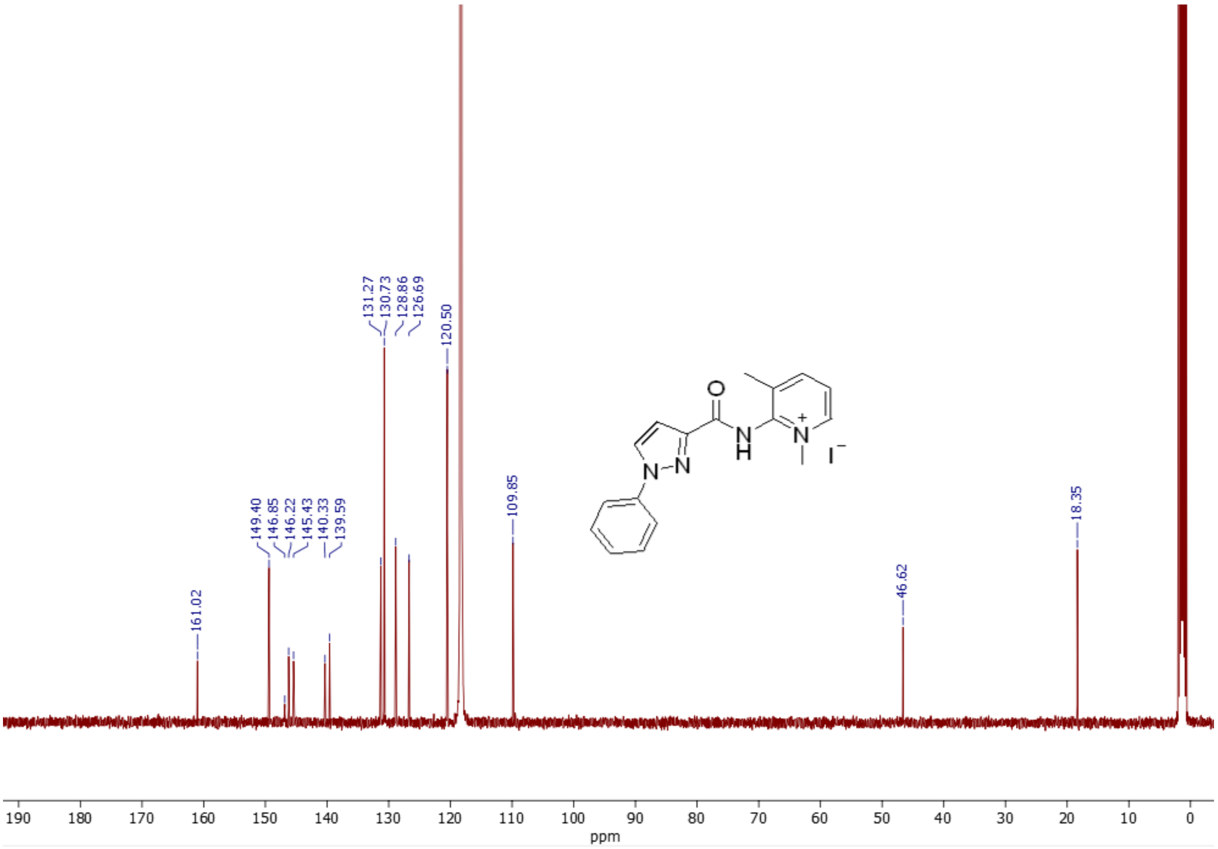

Figure S32: $^{13}C\{^1H\}$ NMR spectrum (CD_3CN, 298 K, 101 MHz) of **3e**

Synthesis of 3f

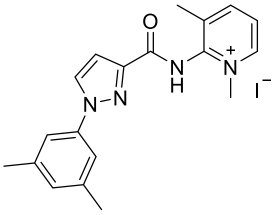

According to the general procedure II from **2f** (1.68 g, 5.5 mmol) and MeI (380 µL, 6.1 mmol) at 60 °C for 16 h, salt **3f** was obtained as an off-white solid (1.87 g, 76%). ^{1}H NMR (300 MHz, CD_3CN, 298K) δ = 10.07 (s, 1H, N*H*), 8.68 (d, $^3J_{HH}$ = 6.2 Hz, 1H, CH_{pyr}), 8.46 (d, $^3J_{HH}$ = 8.0 Hz, 1H, CH_{pyr}), 8.29 (d, $^3J_{HH}$ = 2.6 Hz, 1H, CH_{prz}), 7.88 (dd, $^3J_{HH}$ = 8.0, 6.2 Hz, 1H, CH_{pyr}), 7.64 (s, 2H, CH_{phe}), 7.08 (s, 1H, CH_{phe}) 7.08 (d, $^3J_{HH}$ = 2.6 Hz, 1H, CH_{prz}), 4.30 (s, 3H, NCH_3), 2.50 (s, 3H, pyr-CH_3), 2.40 (s, 6H, phe-CH_3) ppm. ^{13}C{^{1}H} NMR (75 MHz, CD_3CN, 298K) δ = 161.04 (*C*O), 149.28 (*C*H_{pyr}), 146.94 (C_{pyr}), 145.34 (*C*H_{pyr}), 145.84 (C_{prz}), 140.72 (C_{phe}-Me), 140.16 (C_{phe}), 139.66 (C_{pyr}-Me), 130.99 (*C*H_{prz}), 130.18 (*C*H_{phe}) 126.65 (*C*H_{pyr}), 118.08 (*C*H_{phe}) 109.61 (*C*H_{prz}), 46.72 (pyr-N*C*H_3), 21.37 (phe-*C*H_3), 18.41 (pyr-*C*H_3) ppm. HR-MS (m/z): calculated for $C_{19}H_{21}N_4O$ $[M-I]^+$ = 321.1710 ; found: 321.1702.

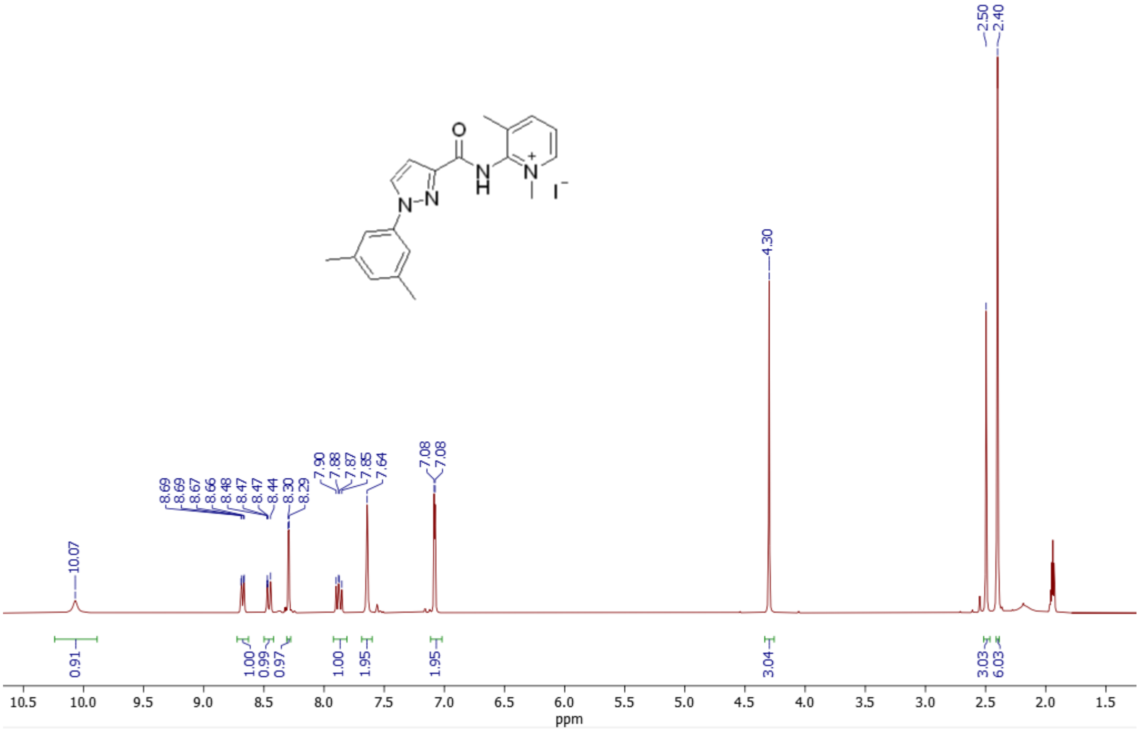

Figure S33: ^{1}H NMR spectrum (CD_3CN, 298 K, 300 MHz) of **3f**

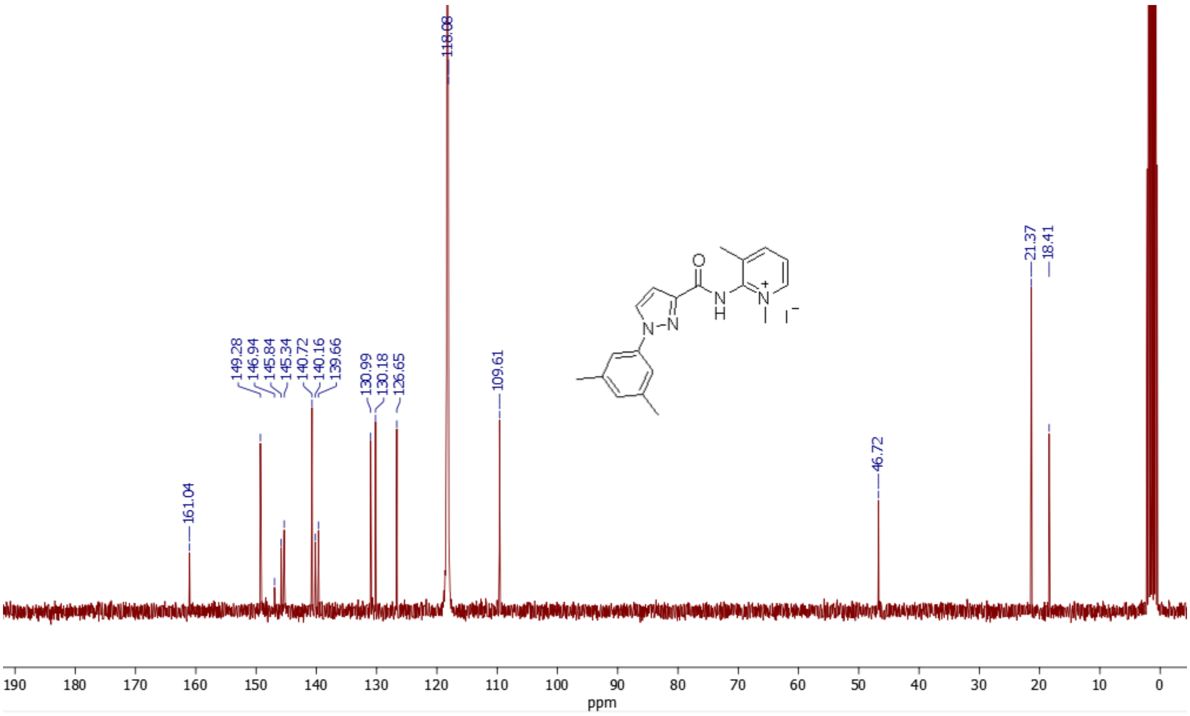

Figure S34: $^{13}C\{^{1}H\}$ NMR spectrum (CD_3CN, 298 K, 75 MHz) of **3f**

Synthesis of 3g

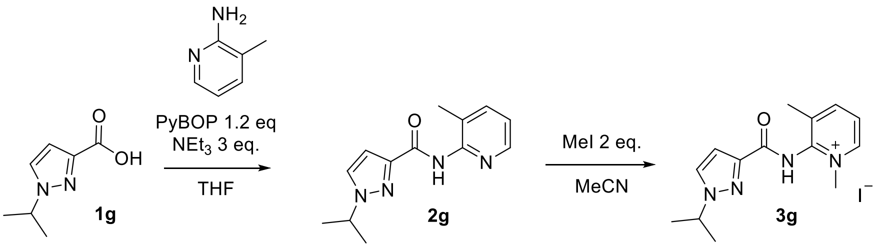

Scheme S3: Synthesis of iodide salt **3g**.

The carboxylic acid **1g** (838 mg, 5.4 mmol), 2-amino-3-methylpyridine (660 µL, 6.54 mmol) and PyBOP (3.40 g, 6.5 mmol) were dissolved in dry THF (20 mL) in a N_2 atmosphere. NEt_3 (2.3 mL, 16 mmol) was added, and the reaction mixture was heated to 60 °C for 30 h. H_2O (40 mL) was added and the mixture was extracted with EtOAc (3 x 20 mL). The combined organic layers were washed with brine (20 mL), dried over $MgSO_4$, filtered, and concentrated under vacuo. The brownish residue was purified by gradient FCC (SiO_2; 0 to 100% EtOAc in pentane; 2nd fraction) and was directly used for the next step without any further purification. The crude carboxamide **2g** was thus dissolved in dry MeCN (10 mL) and MeI (0.66 mL, 10.8 mmol) was added. The mixture was stirred at 60 °C for 8 h and then concentrated to 2 mL. Upon addition of Et_2O (8 mL), a yellowish precipitate formed, which was filtered off, washed with cold Et_2O and thoroughly dried under vacuum to yield **3g** as a yellow powder (1.17 g, 56%). ^{1}H NMR (400 MHz, CD_2Cl_2) δ = 8.73 (d, $^{3}J_{HH}$ = 5.5 Hz, 1H, CH_{PYA}), 8.33 (d, $^{3}J_{HH}$ = 7.9 Hz, 1H,

CH_{PYA}), 7.85 – 7.81 (m, 1H, CH_{PYA}), 7.58 (d, $^{3}J_{HH}$ = 2.4 Hz, 1H, CH_{prz}), 7.02 (d, $^{3}J_{HH}$ = 2.4 Hz, 1H, CH_{prz}), 4.62 (sept, 1H, N_{prz}-C*H*), 4.45 (s, 3H, N_{PYA}-CH_3), 2.57 (s, 3H, C-CH_3), 1.59 (s, 3H, N_{prz}-C-CH_3), 1.57 (s, 3H, N_{prz}-C-CH_3) ppm. ^{13}C{^{1}H} NMR (101 MHz, CD_2Cl_2, 298K) δ = 160.77 (*C*O), 147.99 (C_{prz}), 147.73 (C_{PYA}), 143.59 (*C*H_{PYA}), 143.34 (*C*H_{PYA}), 140.17 (C_{PYA}-Me), 129.54 (*C*H_{prz}), 125.72 (*C*H_{PYA}), 107.79 (*C*H_{prz}), 55.59 (N_{prz}*C*H), 47.30 (N_{PYA}*C*H_3), 23.05 (N_{prz}-C-*C*H_3), 18.99 (C-*C*H_3) ppm. HR-MS (m/z) calculated for $C_{14}H_{19}N_4O$ $[M-I]^+$ = 259.1553; found: 259.1556. Elemental Analysis calculated for $C_{14}H_{19}IN_4O$: C 43.54, H 4.96, N 14.51 found: C 43.86, H 5.0, N 14.17.

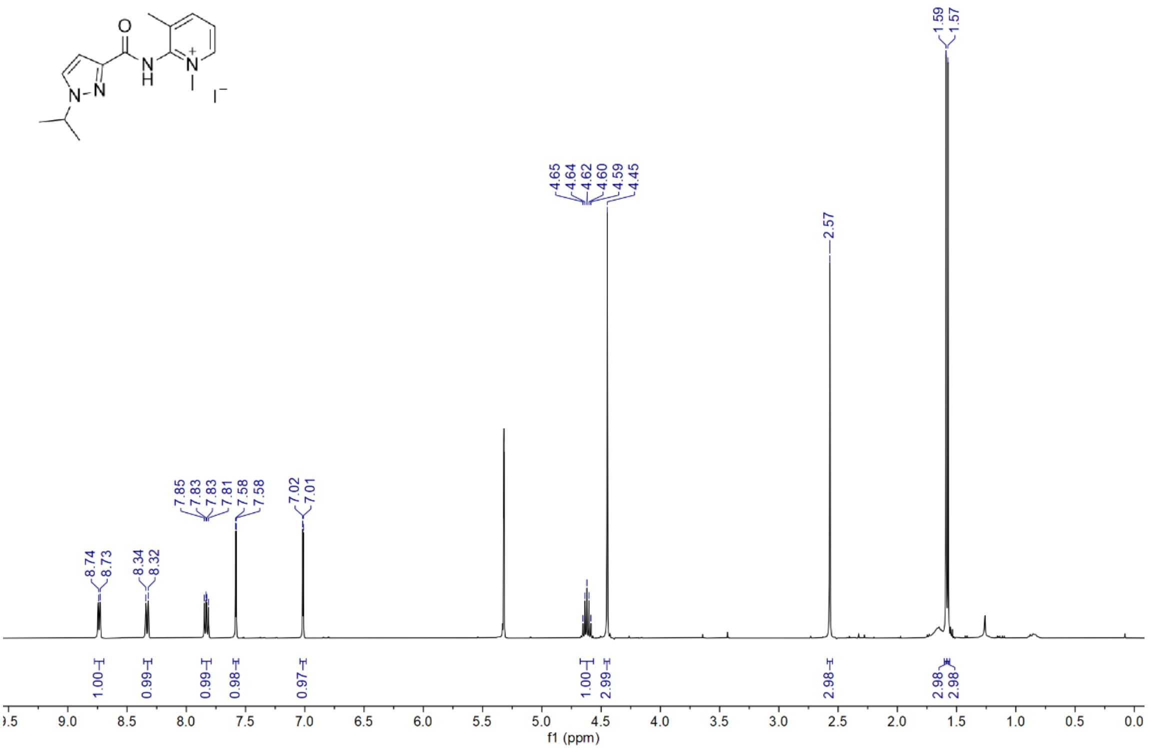

Figure S35: ^{1}H NMR spectrum (CD_2Cl_2, 298 K, 400 MHz) of **3g**

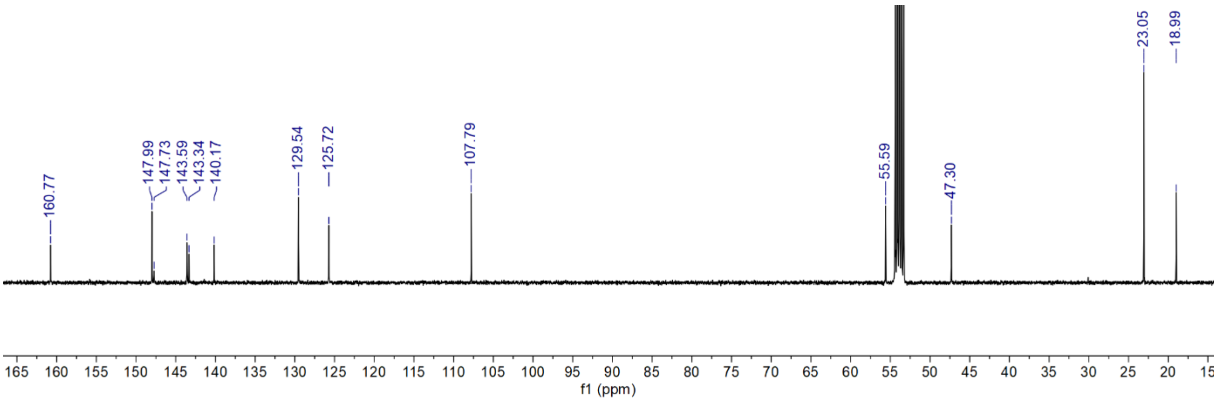

Figure S36: ^{13}C{^{1}H} NMR spectrum (CD_2Cl_2, 298 K, 101 MHz) of **3g**

S.1.6 Synthesis of pyridinium amidate (PYA) 4a–4g

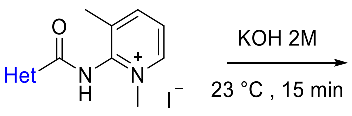

Scheme S4: General method for the preparation of neutral PYA **4a–g**.

General procedure III: The pyridinium salt **3** (2 mmol) was dissolved in KOH_{aq} (2 M, 10 mL, 20 mmol) and the resulting solution was briefly sonicated and stirred at 23 °C for 10 min. The resulting slurry was extracted with CH_2Cl_2 (5 x 15 mL), the combined organic fractions were dried over $MgSO_4$, filtered, and evaporated to dryness to yield the pure pyridinium amidate (PYA) **4**.

Synthesis of PYA 4a

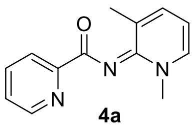

4a

Following the general procedure III, reaction of **3a** (312 mg, 880 µmol) with KOH yielded compound **4a** as a yellow solid (176 mg, 88%). Spectroscopic data are in agreement with literature values.[S1] ^{1}H NMR (300 MHz, CD_2Cl_2, 298 K): δ = 8.61 (d, $^3J_{HH}$ = 4.6 Hz, 1H, CH_{pyr}), 8.18 (d, $^3J_{HH}$ = 7.4 Hz, 1H, CH_{pyr}), 7.76 (t, $^3J_{HH}$ = 7.4 Hz, 1H, CH_{pyr}), 7.66 (d, $^3J_{HH}$ = 6.6 Hz, 1H, CH_{PYA}), 7.57 (d, $^3J_{HH}$ = 7.2 Hz, 1H, CH_{PYA}), 7.31 (dd, $^3J_{HH}$ = 7.4, 4.6 Hz, 1H, CH_{pyr}), 6.73 (dd, $^3J_{HH}$ = 7.2, 6.7 Hz, 1H, CH_{PYA}), 3.86 (s, 3H, PYA-NCH_3), 2.20 (s, 3H, PYA-CCH_3) ppm.

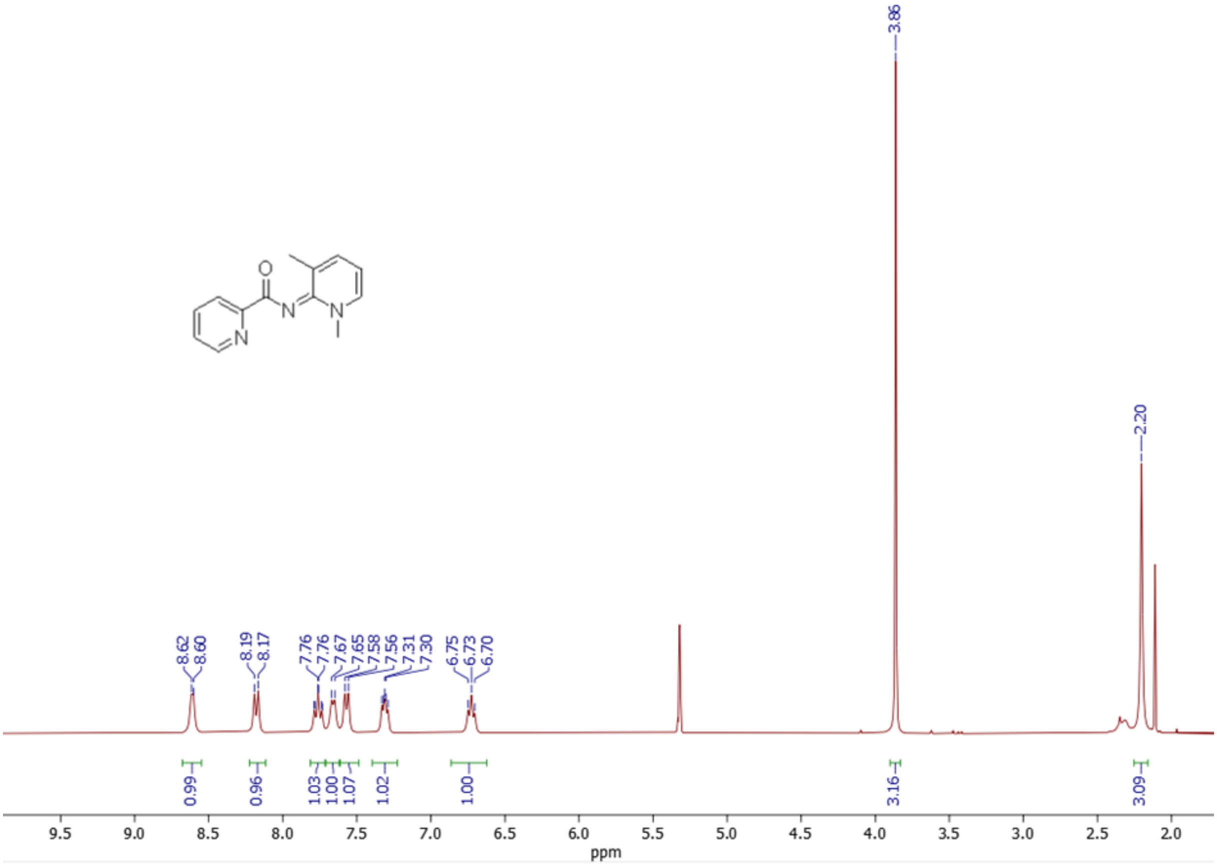

Figure S37: ^{1}H NMR spectrum (CD_2Cl_2, 298 K, 300 MHz) of **4a**

Synthesis of PYA 4b

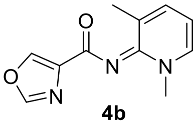

4b

Following the general procedure III and starting from **3b** (690 mg, 2.0 mmol) yielded compound **4b** as a pale-yellow solid (343 mg, 79%). ^{1}H NMR (300 MHz, CD_2Cl_2, 298K) δ = 8.15 (d, $^{4}J_{HH}$ = 1.1 Hz, 1H, CH_{oxa}), 7.85 (d, $^{4}J_{HH}$ = 1.1 Hz, 1H, CH_{oxa}), 7.64 (d, $^{3}J_{HH}$ = 6.6 Hz, 1H, CH_{PYA}), 7.58 (d, $^{3}J_{HH}$ = 7.2 Hz, 1H, CH_{PYA}), 6.74 (dd, $^{3}J_{HH}$ = 7.2 Hz, 6.6 Hz, 1H, CH_{PYA}), 3.85 (s, 3H, NCH_3), 2.22 (s, 3H, pyrCH_3) ppm. ^{13}C{^{1}H} NMR (75 MHz, CD_2Cl_2, 298K) δ = 162.04 (CO), 160.70 (C_{PYA}), 151.04 (CH$_{oxa}$), 141.79 (CH$_{oxa}$), 141.10 (C_{oxa}), 140.98 (CH$_{PYA}$), 137.98 (CH$_{PYA}$), 132.91 (C_{pyr}–Me), 113.72 (CH$_{PYA}$), 43.10 (NCH$_3$), 19.63 (pyr$C$H$_3$) ppm. HR-MS (m/z): calculated for $C_{11}H_{12}N_3O_2$ $[M+H]^+$ = 218.0925 ; found: 218.0914.

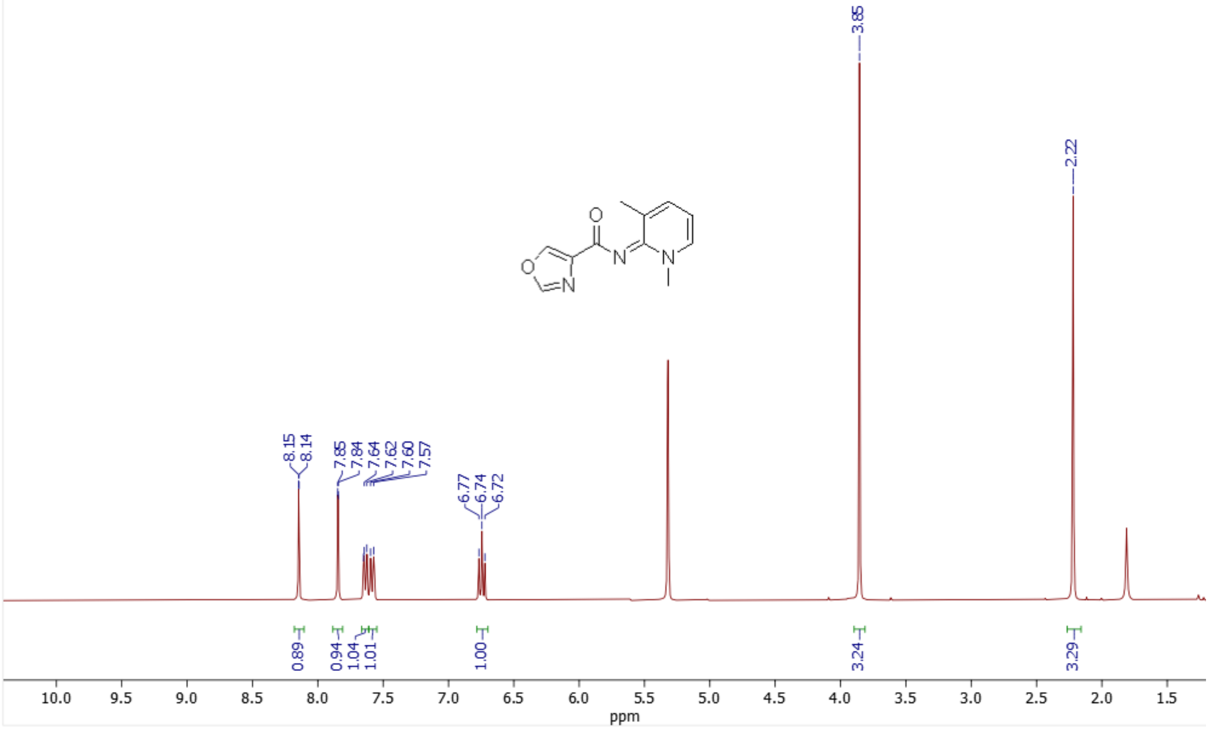

Figure S38: ^{1}H NMR spectrum (CD_2Cl_2, 298 K, 300 MHz) of **4b**

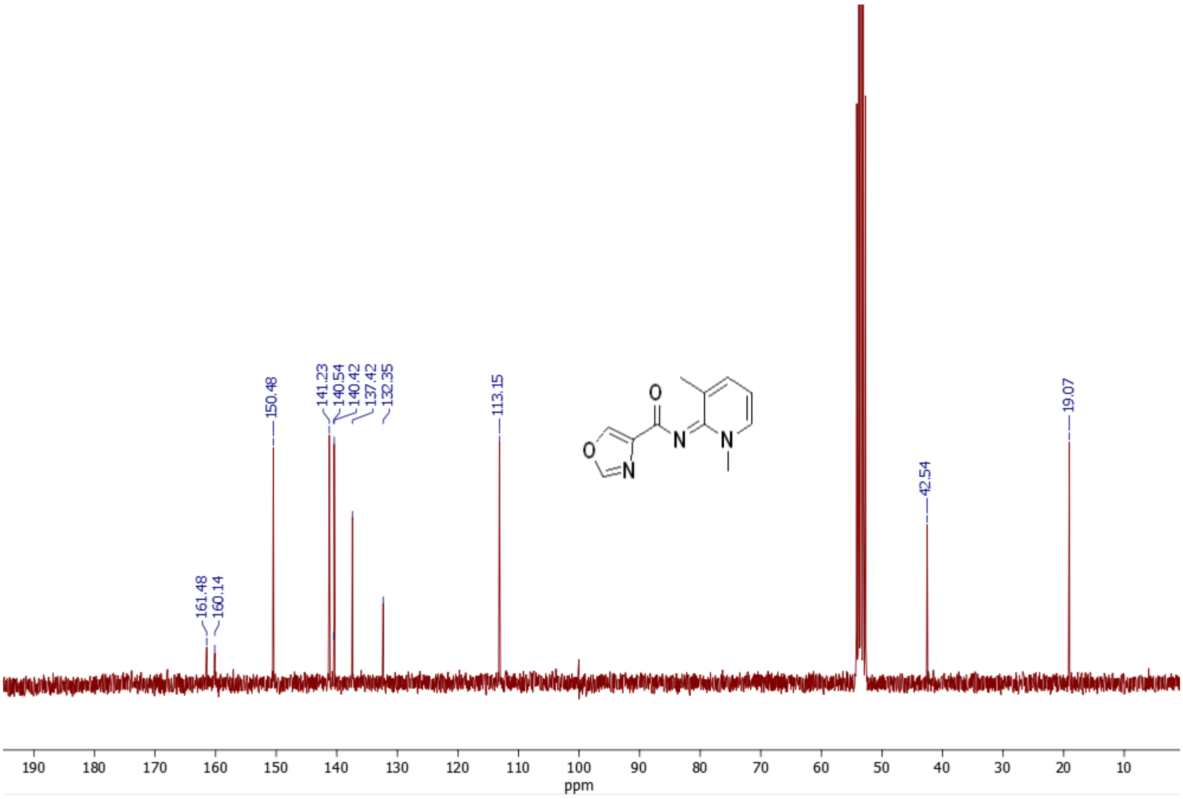

Figure S39: $^{13}C\{^{1}H\}$ NMR spectrum (CD_2Cl_2, 298 K, 75 MHz) of **4b**

Synthesis of PYA 4c

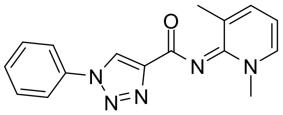

4c

Following the general procedure III and starting from **3c** (420 mg, 1.0 mmol) yielded compound **4c** as a yellow solid (276 mg, 94%). ^{1}H NMR (300 MHz, CD_2Cl_2, 298K) δ = 8.47 (s, 1H, CH_{trz}), 7.86 - 7.75 (m, 2H, CH_{phe}), 7.67 (d, $^{3}J_{HH}$ = 6.7 Hz, 1H, CH_{PYA}), 7.62 (d, $^{3}J_{HH}$ = 7.2 Hz, 1H, CH_{PYA}), 7.59 - 7.52 (m, 2H, CH_{phe}), 7.50 - 7.43 (m, 1H, CH_{phe}), 6.79 (dd, $^{3}J_{HH}$ = 7.2 Hz, 6.7 Hz, 1H, CH_{PYA}), 3.92 (s, 3H, NCH_3), 2.27 (s, 3H, pyrCH_3) ppm. ^{13}C{^{1}H} NMR (75 MHz, CD_2Cl_2, 298K) δ = 161.25 (*C*O), 160.71 (C_{PYA}), 149.10 (C_{trz}), 140.98 (CH_{PYA}), 137.91 (CH_{PYA}), 137.56 (C_{phe}) 132.85 (C_{pyr}–Me), 130.14 (CH_{phe}), 129.05 (CH_{phe}),124.05 (CH_{trz}), 120.85 (CH_{phe}), 113.82 (CH_{PYA}), 43.05 (N*C*H_3), 19.47 (pyr*C*H_3) ppm. HR-MS (m/z): calculated for $C_{16}H_{16}N_5O$ $[M+H]^+$ = 294.1355 ; found: 294.1336.

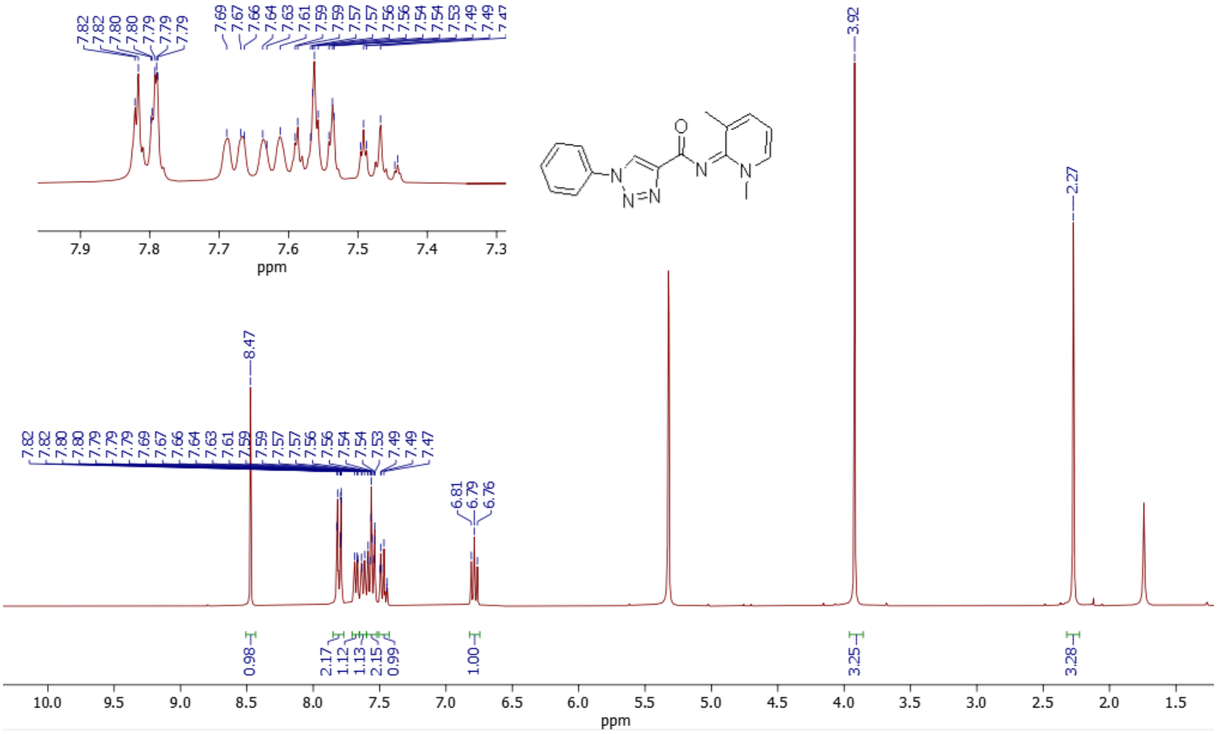

Figure S40: ^{1}H NMR spectrum (CD_2Cl_2, 298 K, 300 MHz) of **4c**

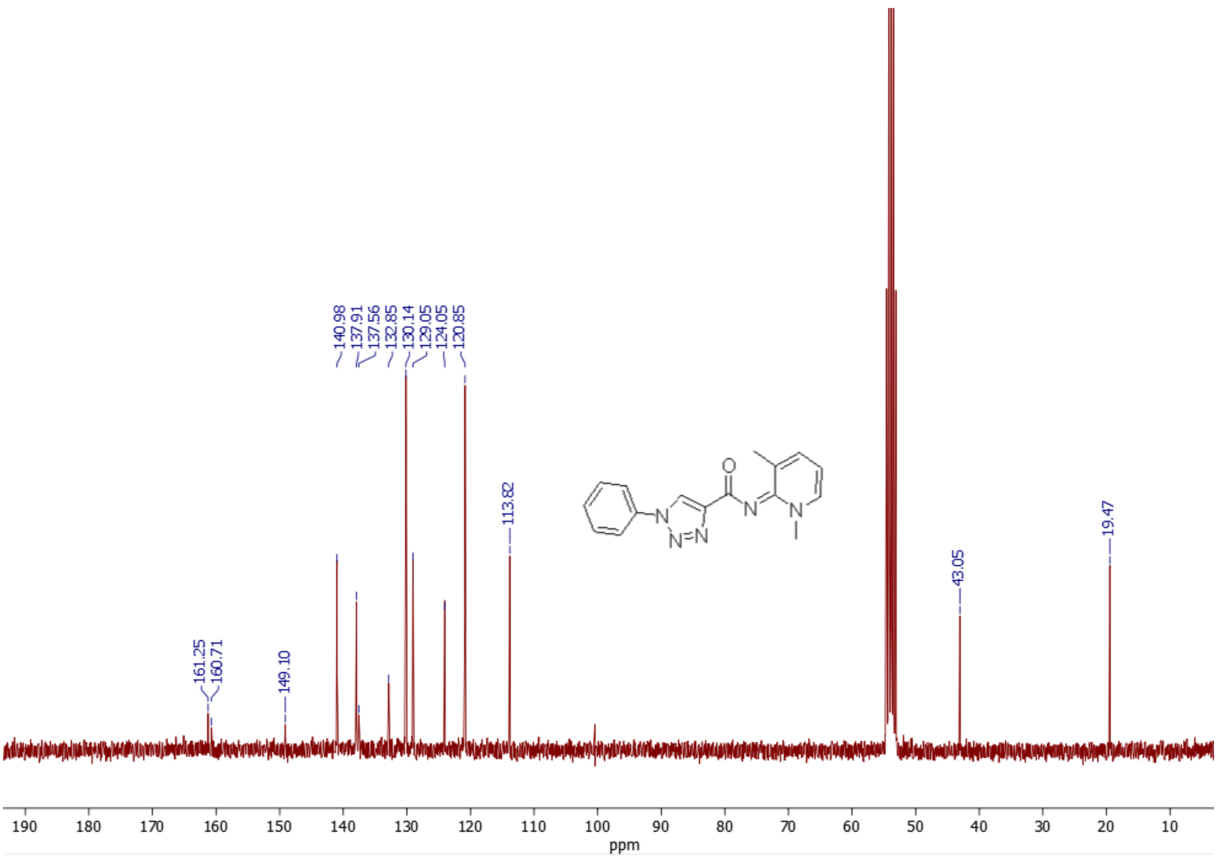

Figure S41: $^{13}C\{^{1}H\}$ NMR spectrum (CD_2Cl_2, 298 K, 75 MHz) of **4c**

Synthesis of PYA 4d

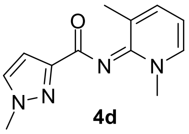

4d

Following the general procedure III and starting from **3d** (500 mg, 1.4 mmol) yielded compound **4d** as a pale-yellow solid (285 mg, 88%). ^{1}H NMR (400 MHz, CD_2Cl_2, 298K) δ = 7.59 (d, $^{3}J_{HH}$ = 6.7 Hz, 1H, CH_{PYA}), 7.52 (d, $^{3}J_{HH}$ = 7.2 Hz, 1H, CH_{PYA}, 7.35 (d, $^{3}J_{HH}$ = 2.2 Hz, 1H, CH_{prz}), 6.69 (d, $^{3}J_{HH}$ = 2.2 Hz, 1H, CH_{prz}), 6.66 (dd, $^{3}J_{HH}$ = 7.2, 6.7 Hz, 2H, CH_{PYA}), 3.90 (s, 3H, prz-NCH_3), 3.82 (s, 3H, pyr-NCH_3), 2.19 (s, 3H, pyr-CH_3) ppm. $^{13}C\{^{1}H\}$ NMR (101 MHz, CD_2Cl_2, 298K) δ = 163.90 (*C*O), 160.20 (C_{PYA}), 151.79 (C_{prz}), 140.25 (*C*H_{PYA}), 137.69 (*C*H_{PYA}), 132.46 (C_{pyr}–Me), 130.67 (*C*H_{prz}), 112.65 (*C*H_{PYA}), 107.75 (*C*H_{prz}), 42.77 (pyr-N*C*H_3), 39.48 (prz-N*C*H_3), 19.60 (pyr-*C*H_3) ppm. HR-MS (m/z): calculated for $C_{12}H_{15}N_4O$ $[M+H]^+$ = 231.1246 ; found: 231.1238.

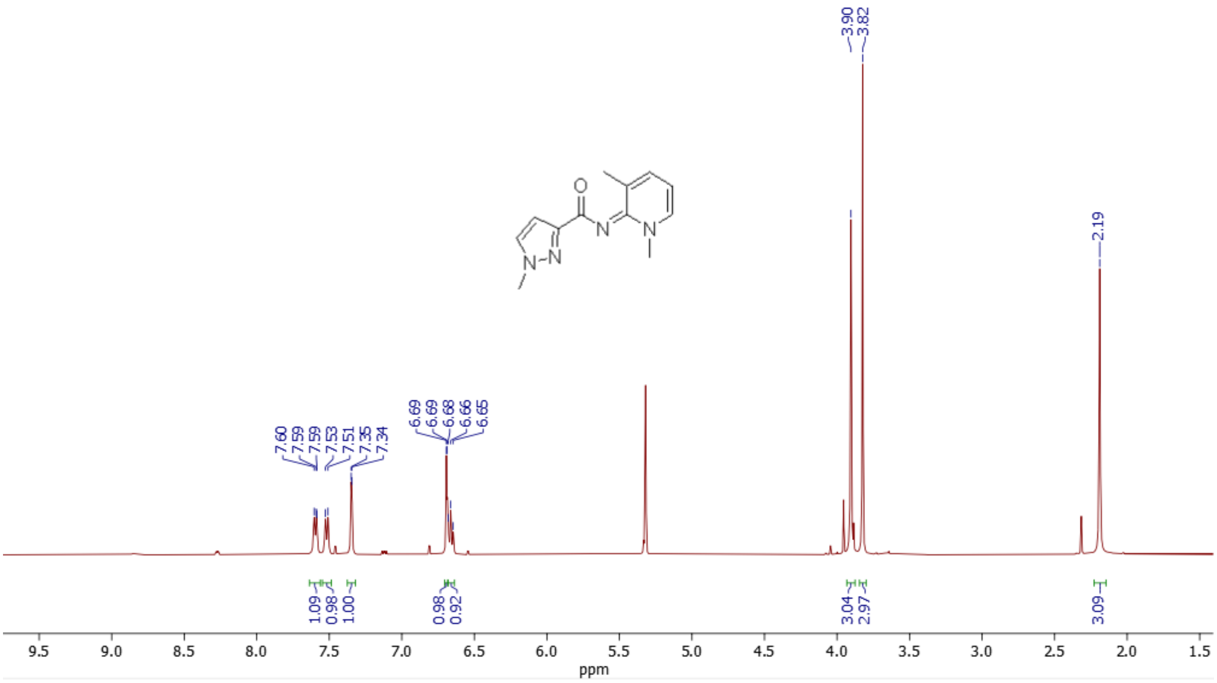

Figure S42: ^{1}H NMR spectrum (CD_2Cl_2, 298 K, 400 MHz) of **4d**

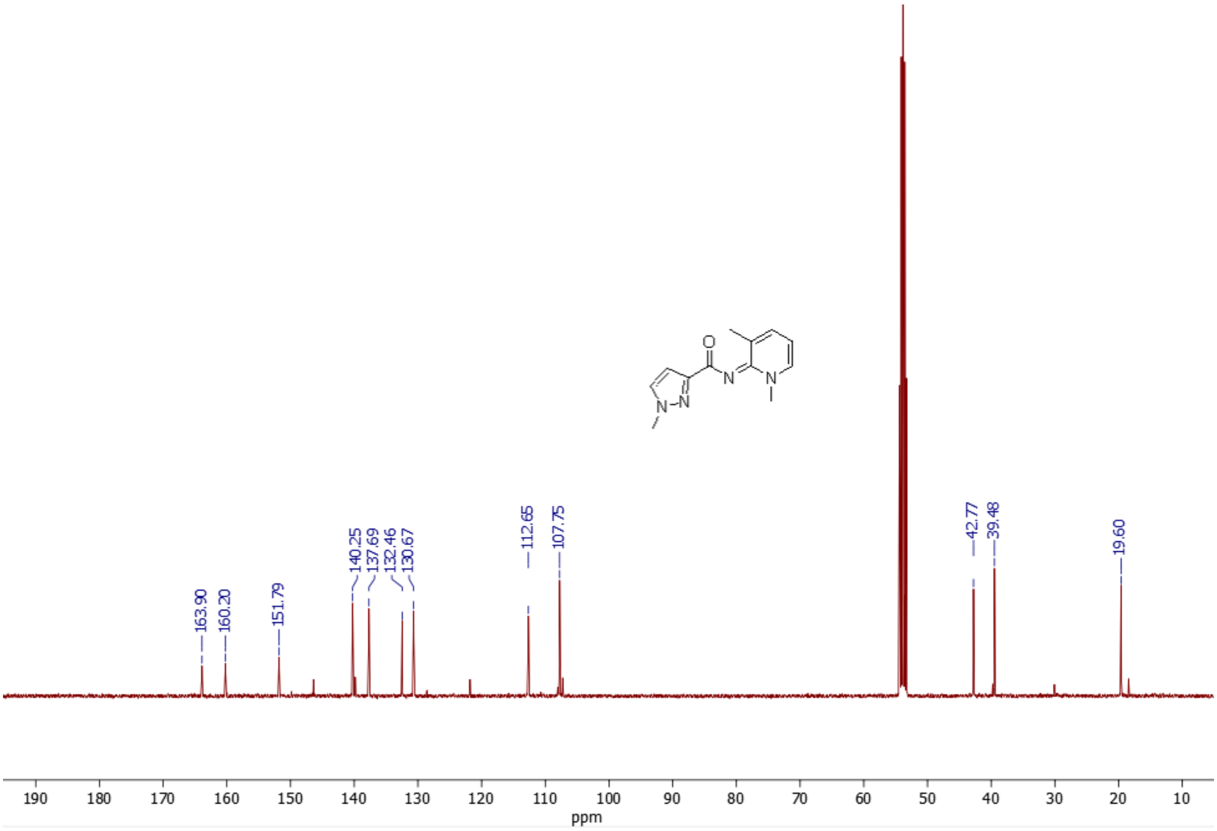

Figure S43: $^{13}C\{^{1}H\}$ NMR spectrum (CD_2Cl_2, 298 K, 101 MHz) of **4d**

Synthesis of PYA 4e

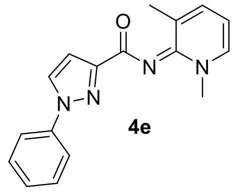

4e

Following the general procedure III and starting from **3e** (800 mg, 1.9 mmol) yielded compound **4e** as a white solid (556 mg, 98%). ^{1}H NMR (300 MHz, CD_2Cl_2, 298K) δ = 7.95 (d, $^3J_{HH}$ = 2.5 Hz, 1H, CH_{prz}), 7.75 (d, $^3J_{HH}$ = 7.7 Hz, 2H, CH_{phe}), 7.63 (d, $^3J_{HH}$ = 6.6 Hz, 1H, CH_{PYA}), 7.57 (d, $^3J_{HH}$ = 7.2 Hz, 1H, CH_{PYA}), 7.46 (t, $^3J_{HH}$ = 7.7 Hz, 2H, CH_{phe}), 7.31 (d, $^3J_{HH}$ = 7.7 Hz, 1H, CH_{phe}), 6.93 (d, $^3J_{HH}$ = 2.5 Hz, 1H, CH_{prz}), 6.72 (dd, $^3J_{HH}$ = 7.2 Hz, 6.6 Hz, 1H, CH_{PYA}), 3.88 (s, 3H, NCH_3), 2.25 (s, 3H, pyr-CH_3) ppm. ^{13}C{^{1}H} NMR (101 MHz, CD_2Cl_2, 298K) δ = 163.23 (*C*O), 160.48 (C_{PYA}), 153.60 (C_{prz}), 140.71 (C_{phe}), 140.53 (*C*H_{PYA}), 137.79 (*C*H_{PYA}), 132.70 (C_{PYA}–Me), 129.73 (*C*H_{phe}), 127.56 (*C*H_{phe}), 126.82 (*C*H_{prz}), 119.61 (*C*H_{phe}), 113.15 (*C*H_{PYA}), 109.72 (*C*H_{prz}), 42.91 (pyr-N*C*H_3), 19.59 (pyr-*C*H_3) ppm. HR-MS (m/z): calculated for $C_{17}H_{17}N_4O$ $[M+H]^+$ = 293.1402 ; found: 293.1394.

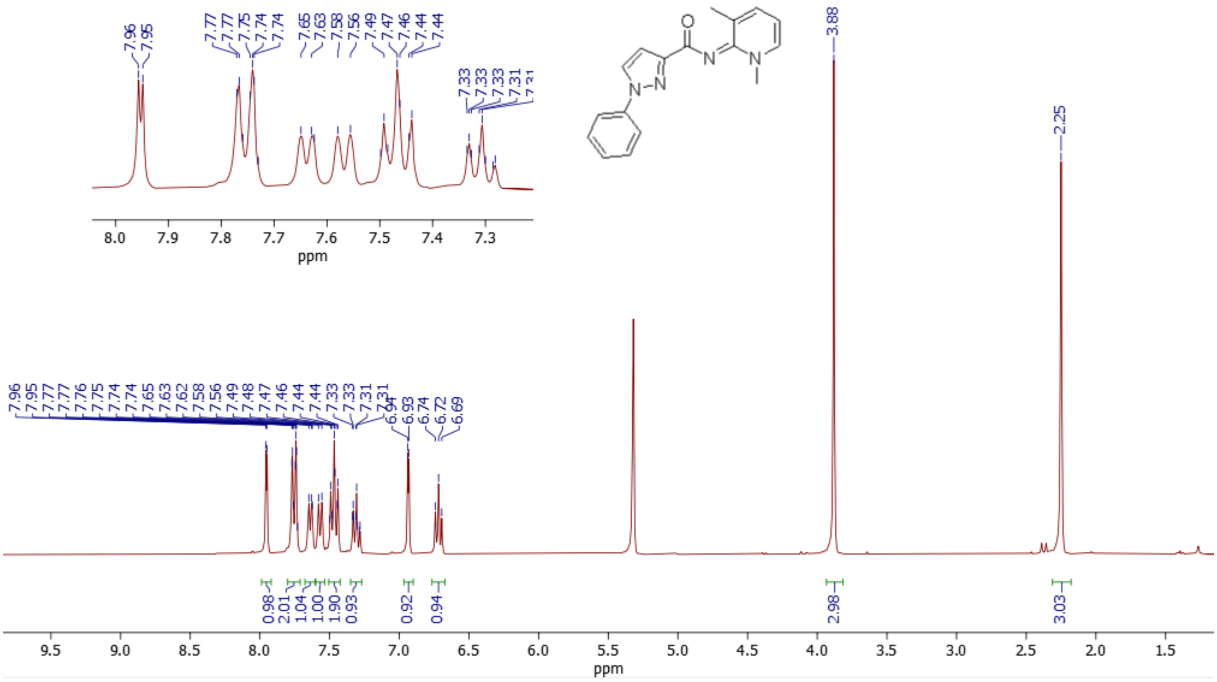

Figure S44: ^{1}H NMR spectrum (CD_2Cl_2, 298 K, 300 MHz) of **4e**

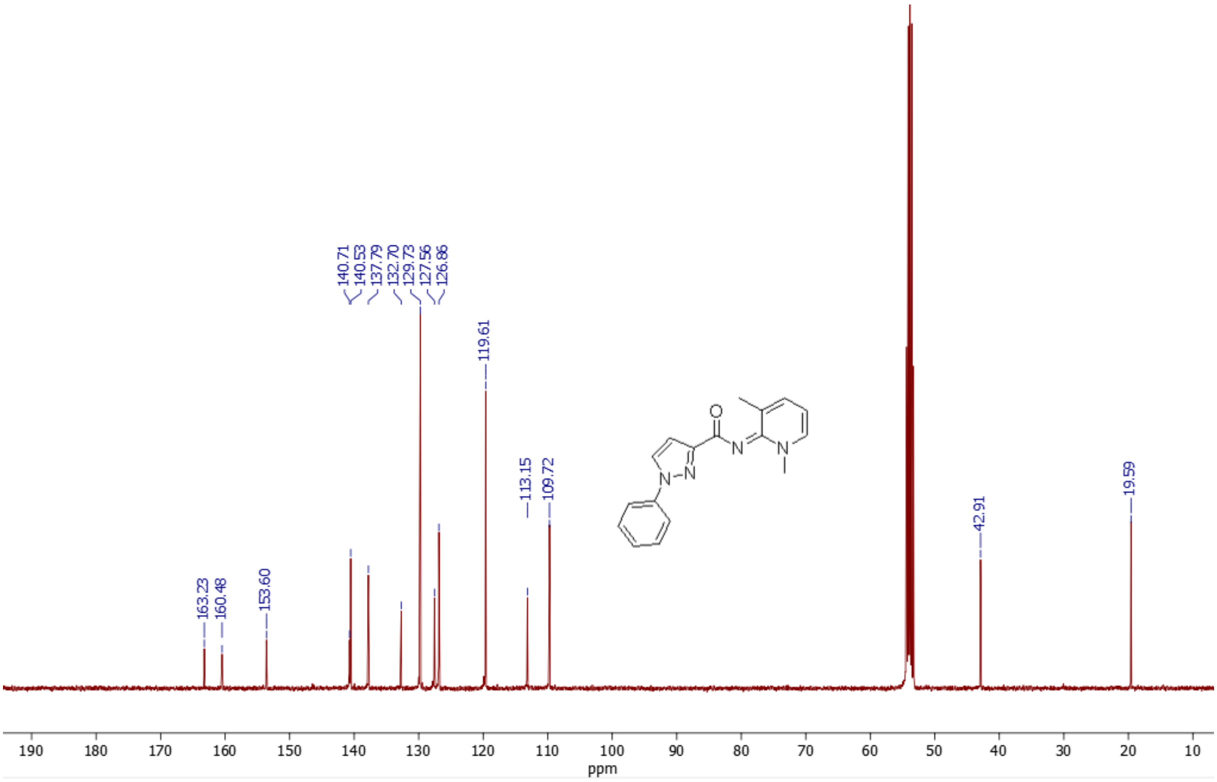

Figure S45: $^{13}C\{^{1}H\}$ NMR spectrum (CD_2Cl_2, 298 K, 75 MHz) of **4e**

Synthesis of PYA 4f

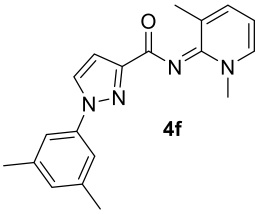

4f

Following the general procedure III and starting from **3f** (1.0 g, 2.23 mmol) yielded compound **4f** as a white solid (618 mg, 86%). ^{1}H NMR (300 MHz, CD_2Cl_2, 298K) δ = 7.92 (d, $^{3}J_{HH}$ = 2.5 Hz, 1H, CH_{prz}), 7.63 (dd, J_{HH} = 6.6, 1.7 Hz, 1H, CH_{PYA}), 7.57 (dd, J_{HH} = 7.3, 1.7 Hz, 1H, CH_{PYA}), 6.96 (s, 1H, CH_{phe}), 6.91 (d, $^{3}J_{HH}$ = 2.5 Hz, 1H, CH_{prz}), 6.71 (dd, $^{3}J_{HH}$ = 7.3 Hz, 6.6 Hz, 1H, CH_{PYA}), 3.88 (s, 3H, NCH_3), 2.38 (s, 6H, phe-CH_3), 2.25 (s, 3H, pyr-CH_3) ppm. $^{13}C\{^{1}H\}$ NMR (101 MHz, CD_2Cl_2, 298K) δ = 163.41 (*C*O), 160.55 (C_{PYA}), 153.37 (C_{prz}), 140.61 (C_{phe}), 140.43 (CH_{PYA}), 139.73 (C_{phe}-Me), 137.74 (CH_{PYA}), 132.72 (C_{PYA}–Me), 128.52 (CH_{phe}), 127.50 (CH_{prz}), 117.36 (CH_{phe}), 112.98 (CH_{PYA}), 109.50 (CH_{prz}), 42.89 (pyr-NCH_3), 21.47 (phe-CH_3), 19.63 (pyr-CH_3) ppm. HR-MS (m/z): calculated for $C_{19}H_{21}N_4O$ $[M+H]^+$ = 321.1715 ; found: 321.1703.

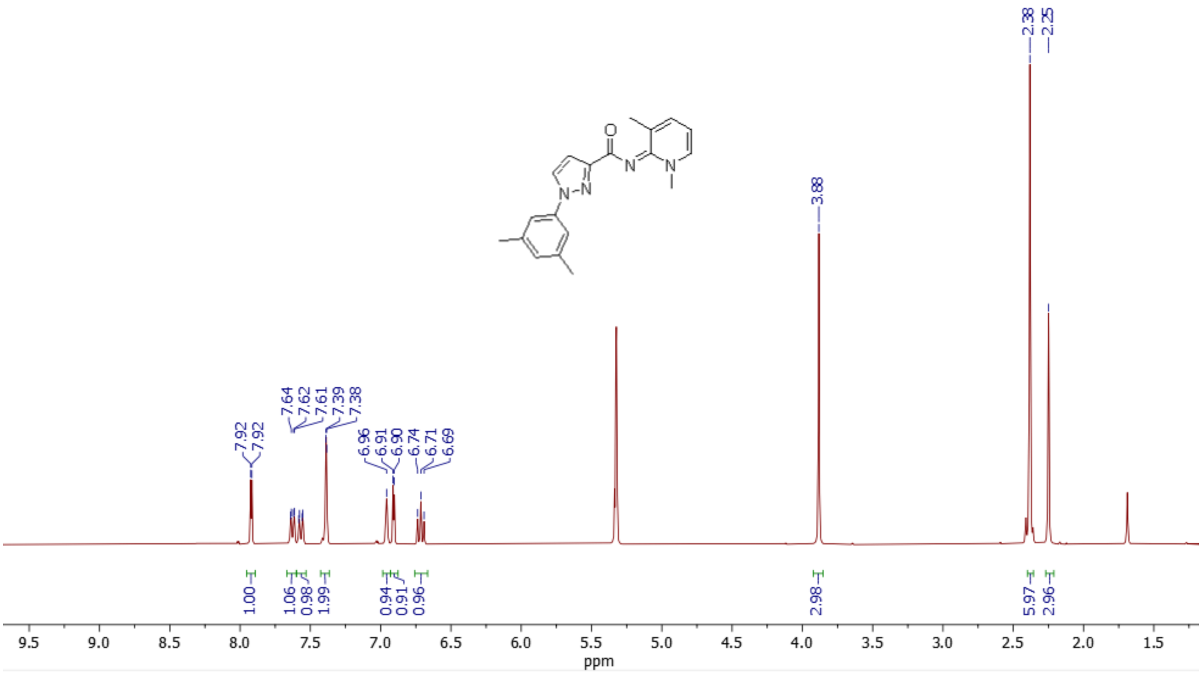

Figure S46: ^{1}H NMR spectrum (CD_2Cl_2, 298 K, 300 MHz) of **4f**

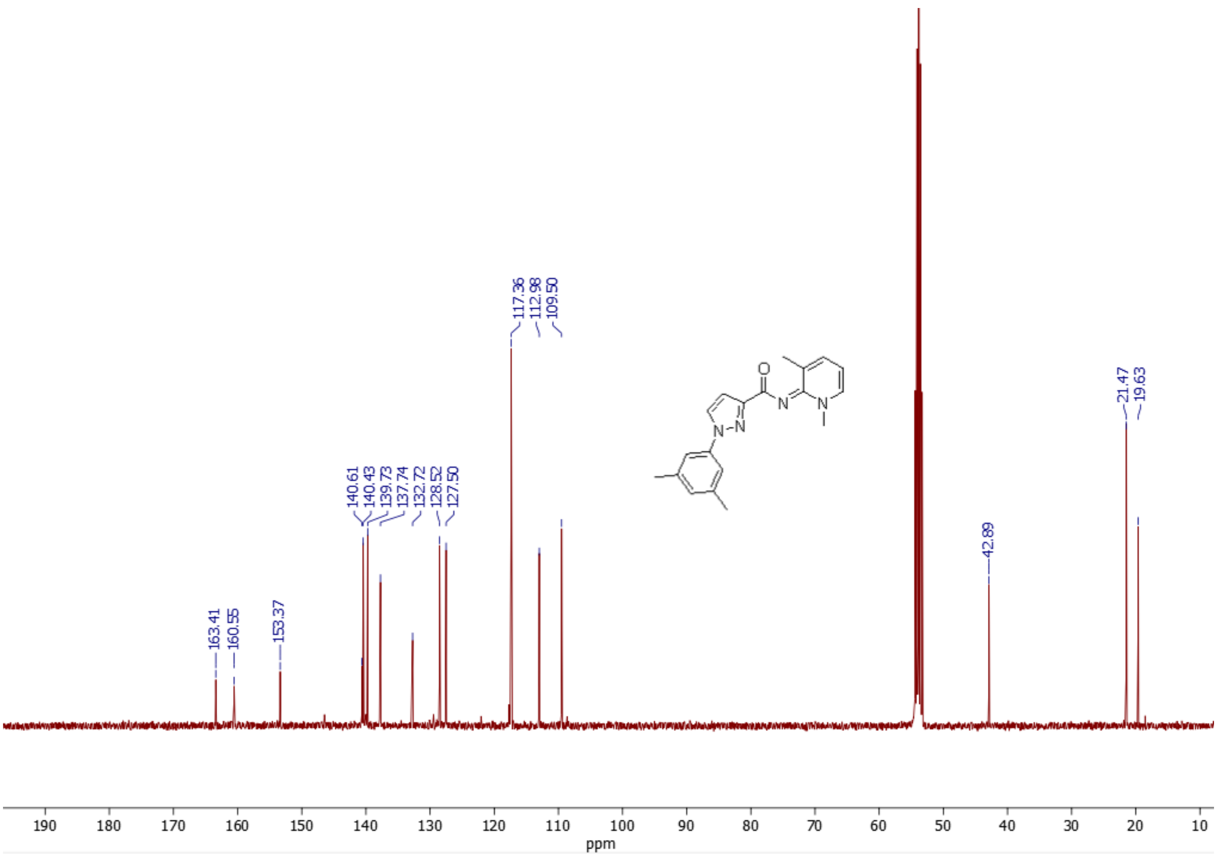

Figure S47: $^{13}C\{^{1}H\}$ NMR spectrum (CD_2Cl_2, 298 K, 101 MHz) of **4f**

Synthesis of PYA 4g

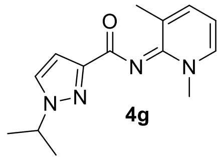

4g

Following the general procedure III and starting from **3g** (800 mg, 2.07 mmol) yielded compound **4g** as a white solid (490 mg, 82%). ^{1}H NMR (400 MHz, CD_2Cl_2) δ = 7.59 (d, $^3J_{HH}$ = 6.3 Hz, 1H, CH_{PYA}), 7.52 (d, $^3J_{HH}$ = 6.8 Hz, 1H, CH_{PYA}), 7.41 (d, $^3J_{HH}$ = 2.3 Hz, 1H, CH_{prz}), 6.69 (d, $^3J_{HH}$ = 2.3 Hz, 1H, CH_{prz}), 6.68 – 6.64 (m, 1H, CH_{PYA}), 4.53 (sept, 1H, N_{prz}-CH), 3.83 (s, 3H, N_{PYA}-CH_3), 2.2 (s, 3H, C-CH_3), 1.51 (s, 3H, N_{prz}-C-CH_3), 1.49 (s, 3H, N_{prz}-C-CH_3) ppm. ^{13}C{^{1}H} NMR (101 MHz, CD_2Cl_2, 298K) δ = 164.07 (*C*O), 160.34 (C_{prz}), 151.13 (C_{PYA}), 140.21 (CH_{PYA}), 137.66 (CH_{PYA}), 132.56 (C_{PYA}-Me), 126.95 (CH_{prz}), 112.66 (CH_{PYA}), 107.30 (CH_{prz}), 54.51 ($N_{prz}CH$), 42.79 ($N_{PYA}CH_3$), 23.14 (N_{prz}-C-CH_3), 19.6 (C-CH_3) ppm. HR-MS (m/z) calculated for $C_{14}H_{19}N_4O$ $[M+H]^+$ = 259.1553; found: 259.1557.

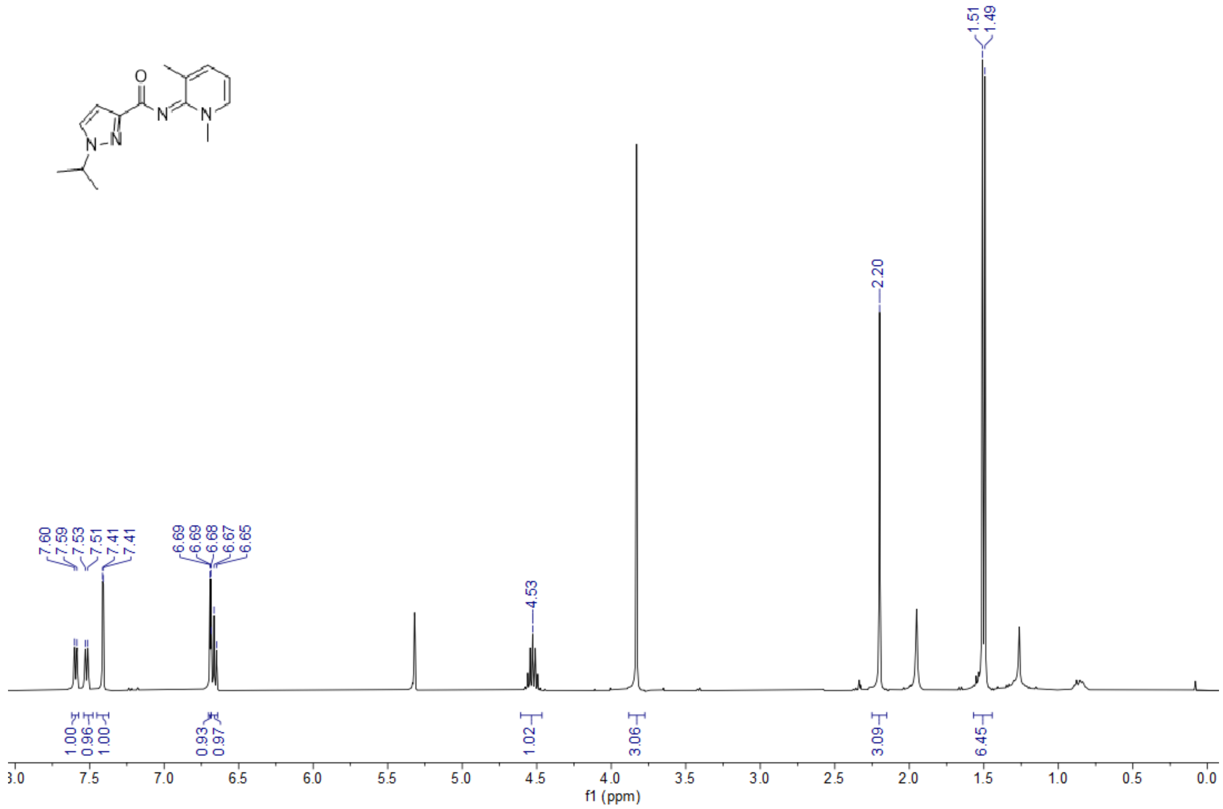

Figure S48: ^{1}H NMR spectrum (CD_2Cl_2, 298 K, 400 MHz) of **4g**

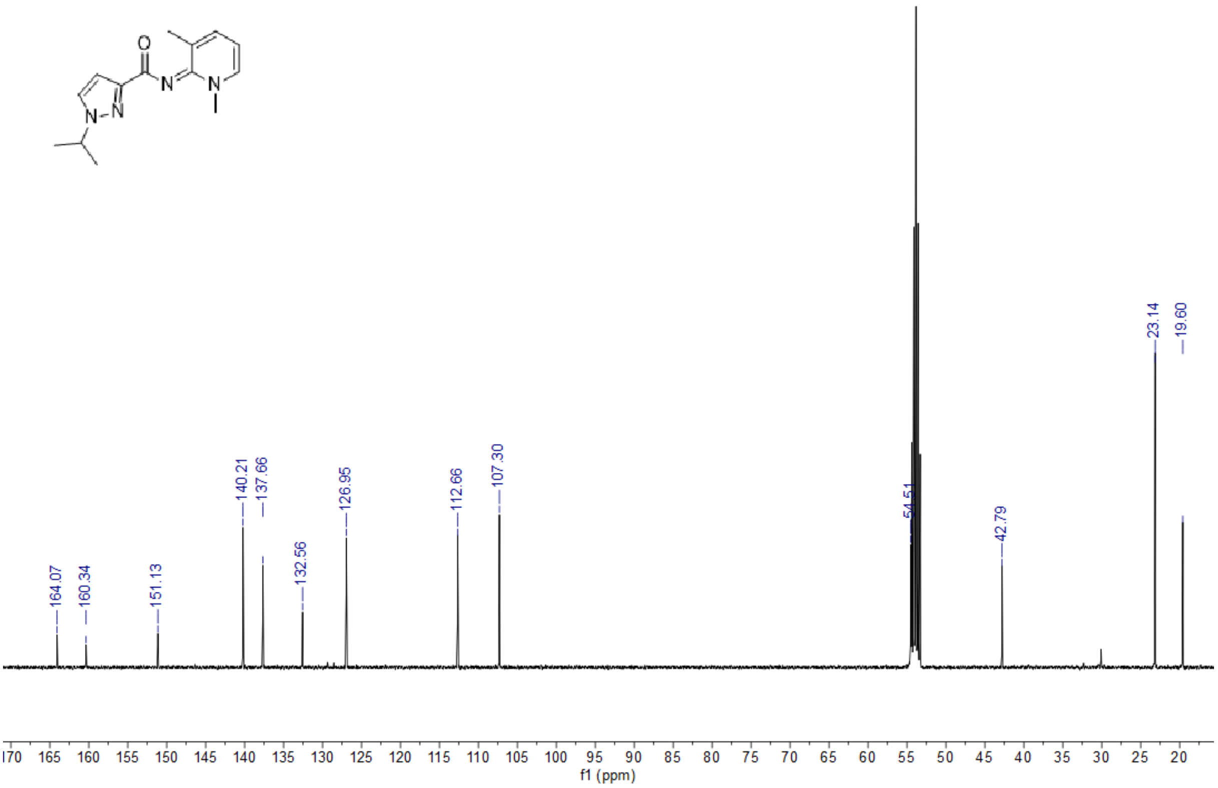

Figure S49: $^{13}C\{^1H\}$ NMR spectrum (CD_2Cl_2, 298 K, 101 MHz) of **4g**

S.1.7 Synthesis of PYA palladium complexes 5a–5g

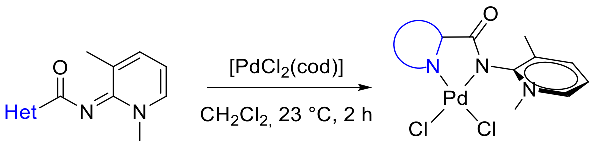

Scheme S5: General method for the preparation of heteroaryl-PYA Pd complexes **5a–g**.

General procedure IV: Under a N_2 atmosphere, the neutral PYA **4** (0.5 mmol, 1.05 eq.) and [$PdCl_2$(cod)] (0.5 mmol, 1 eq.) were dissolved in dry CH_2Cl_2 (5 mL). The mixture was stirred at 23 °C for 2 h and then concentrated to 2 mL. Addition of Et_2O (10 mL) gave a precipitate, which was filtered off, washed several times with Et_2O, and then dried in vacuo to give the corresponding Pd complex **5**.

Synthesis of complex 5a

Following the general procedure IV, reaction of **4a** (270 mg, 1.19 mmol) and [$PdCl_2$(cod)] (308 mg, 1.08 mmol) yielded complex **5a** as a bright orange solid (424 mg, 97%). Spectroscopical data are in agreement with literature values.[S1] ^{1}H NMR (400 MHz, CD_3CN, 298 K): δ = 9.15 (d, J_{HH} = 5.6, 1.5 Hz, 1H, CH_{pyr}), 8.38 (d, $^3J_{HH}$ = 6.2, 1H, CH_{PYA}), 8.23 (d, $^3J_{HH}$ = 7.7, 1H, CH_{PYA}), 8.16 (td, J_{HH} = 7.7, 1.5 Hz, 1H, CH_{pyr}), 7.88 (dd, J_{HH} = 7.7, 1.6 Hz, 1H, CH_{pyr}), 7.69 (ddd, J_{HH} = 7.7, 5.6, 1.6 Hz, 1H, CH_{pyr}), 7.60 (d, $^3J_{HH}$ = 7.7, 6.2, 1H, CH_{PYA}), 4.26 (s, 3H, NCH_3), 2.49 (s, 3H, PYA-CCH_3) ppm.

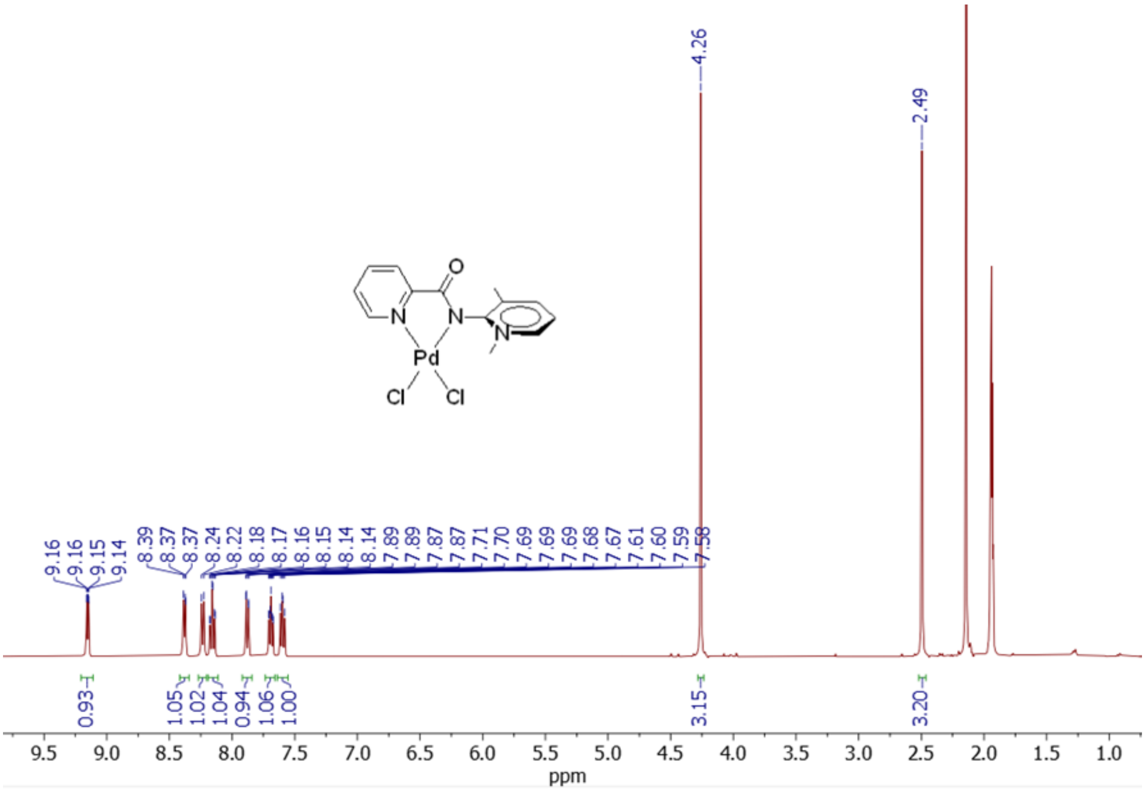

Figure S50: ^{1}H NMR spectrum (CD_2Cl_2, 298 K, 400 MHz) of **5a**

Synthesis of complex 5b

Complex **5b** was prepared according to the general procedure IV from ligand **4b** (120 mg, 0.5 mmol) and [$PdCl_2$(cod)] (159.8 mg, 0.5 mmol) and isolated as a yellow powder (190 mg, 95%). Crystals suitable for Xray diffraction were grown by slow diffusion of Et_2O into a CH_2Cl_2 solution of **5b**. ^{1}H NMR (400 MHz, CD_2Cl_2) δ = 8.50 (d, $^3J_{HH}$ = 1.1 Hz, 1H, CH_{oxa}), 8.19 (s, 2H, CH_{oxa} + CH_{PYA}), 8.15 (d, $^3J_{HH}$ = 7.8 Hz, 1H, CH_{PYA}), 7.51 (dd, $^3J_{HH}$ = 7.8, 6.2 Hz, 1H, CH_{PYA}), 4.33 (s, 3H, NCH_3), 2.57 (s, 3H, C-CH_3) ppm. ^{13}C{^{1}H} NMR (101 MHz, CD_2Cl_2, 298K) δ = 163.45 (*C*O), 157.94 (*C*$_{PYA}$), 152.28 (*C*H_{oxa}), 146.05 (*C*H_{PYA}), 141.55 (*C*H_{oxa}), 141.04 (*C*$_{oxa}$), 138.94 (*C*H_{PYA}), 136.63 (*C*$_{PYA}$Me), 122.98 (*C*H_{PYA}), 45.53 (N*C*H_3), 18.65 (pyr-*C*H_3) ppm. HR-MS (m/z) calculated for $C_{11}H_{11}ClN_3O_2Pd$ $[M-Cl]^+$ = 357.9575; found: 357.9565. Elemental Analysis calculated for $C_{11}H_{11}Cl_2N_3O_2Pd$ x 0.2 CH_2Cl_2 (%): C 32.69, H 2.79, N 10.21%; found: C 32.94, H 2.65, N 9.77%.

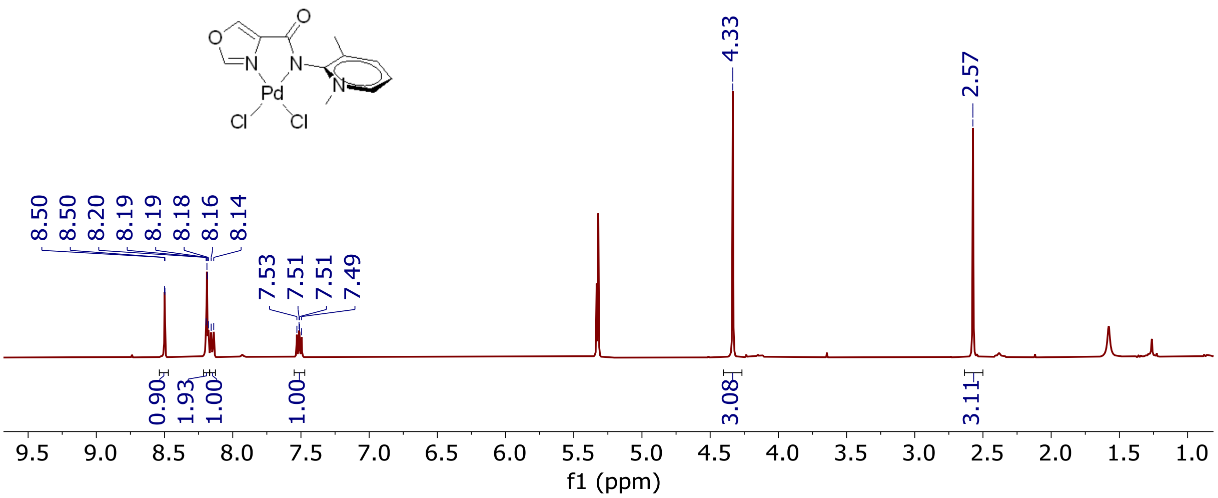

Figure S51: ^{1}H NMR spectrum (CD_2Cl_2, 298 K, 400 MHz) of **5b**

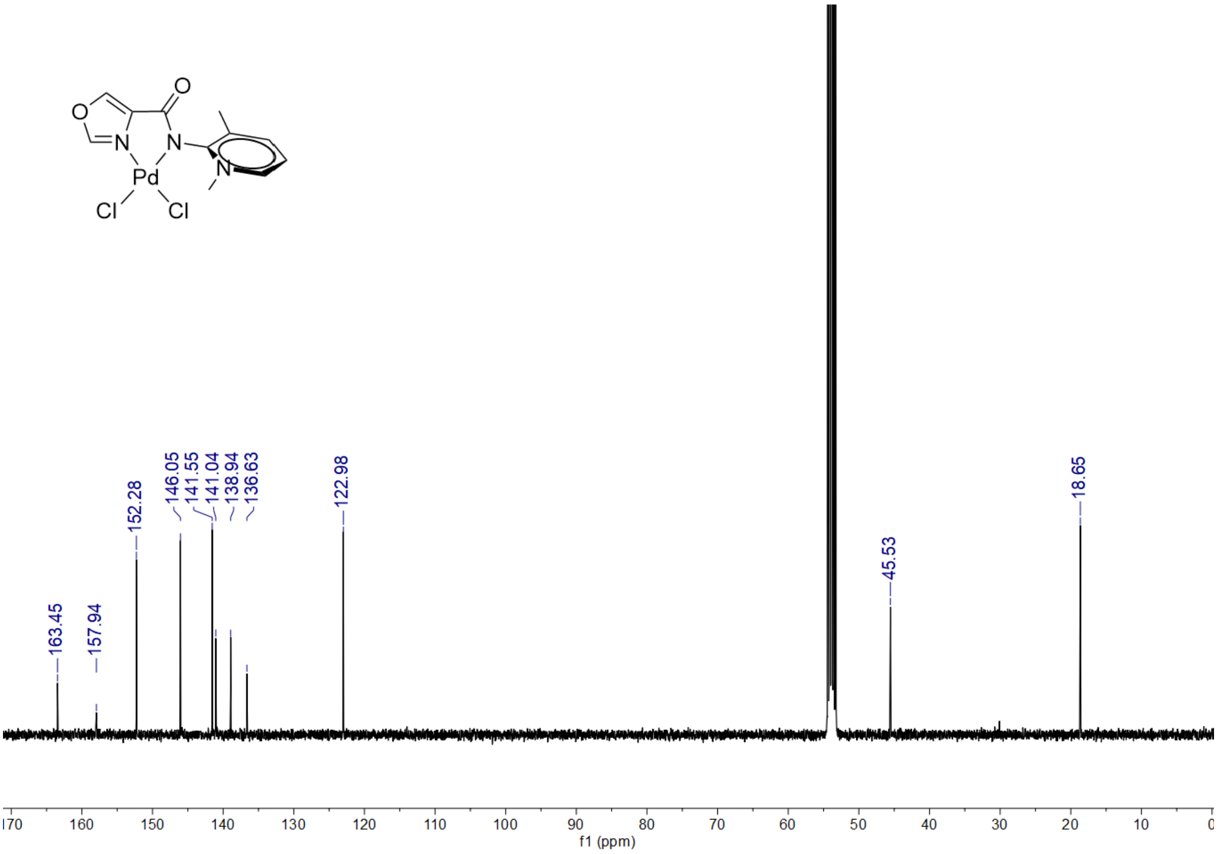

Figure S52: $^{13}C\{^{1}H\}$ NMR spectrum (CD_2Cl_2, 298 K, 101 MHz) of **5b**

Synthesis of complex 5c

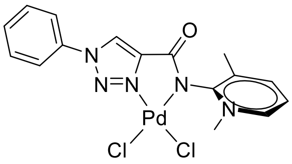

5c

Complex **5c** was prepared according to the general procedure IV from ligand **4c** (50.1 mg, 0.17 mmol) and $[PdCl_2(cod)]$ (74.21 mg, 0.26 mmol) and was obtained as a yellow powder (68.2 mg, 89%). ^{1}H NMR (400 MHz, DMSO-*d*$_6$) δ = 9.56 (s, 1H, CH_{trz}), 8.80 (d, $^3J_{HH}$ = 6.2 Hz, 1H, CH_{PYA}), 8.40 (d, $^3J_{HH}$ = 7.6 Hz, 1H, CH_{PYA}), 7.93 (m, 2H, CH_{Phe}), 7.77 (apparent triplet, 1H, CH_{PYA}), 7.67 (m, 3H, CH_{Phe}), 4.26 (s, 3H, N$_{PYA}$CH_3), 2.46 (s, 3H, C$_{PYA}$CH_3) ppm. ^{13}C{^{1}H} NMR (101 MHz, CD_2Cl_2, 298K) δ = 162.30 (*C*O), 156.00 (C_{PYA}), 145.82 (*C*H$_{PYA}$), 143.82 (C_{trz}), 142.41 (*C*H$_{PYA}$), 136.67 (C_{Phe}), 135.98 (C_{PYA}Me), 130.35 (*C*H$_{Phe}$), 130.05 (*C*H$_{Phe}$), 125.97 (*C*H$_{PYA}$), 123.01 (*C*H$_{trz}$), 121.35 (*C*H$_{Phe}$), 43.96 (N$_{PYA}$*C*H$_3$), 17.54 (C$_{PYA}$*C*H$_3$) ppm. HR-MS (m/z) calculated for $C_{18}H_{18}ClN_6OPd$ [M-Cl+CH_3CN]$^+$ = 475.03; found: 475.0253. Elemental Analysis calculated for $C_{16}H_{15}Cl_2N_5OPd$ x 0.5 CH_2Cl_2: C 38.62, H 3.14, N 13.65%; found: C 38.77, H 3.12, N 13.46%.

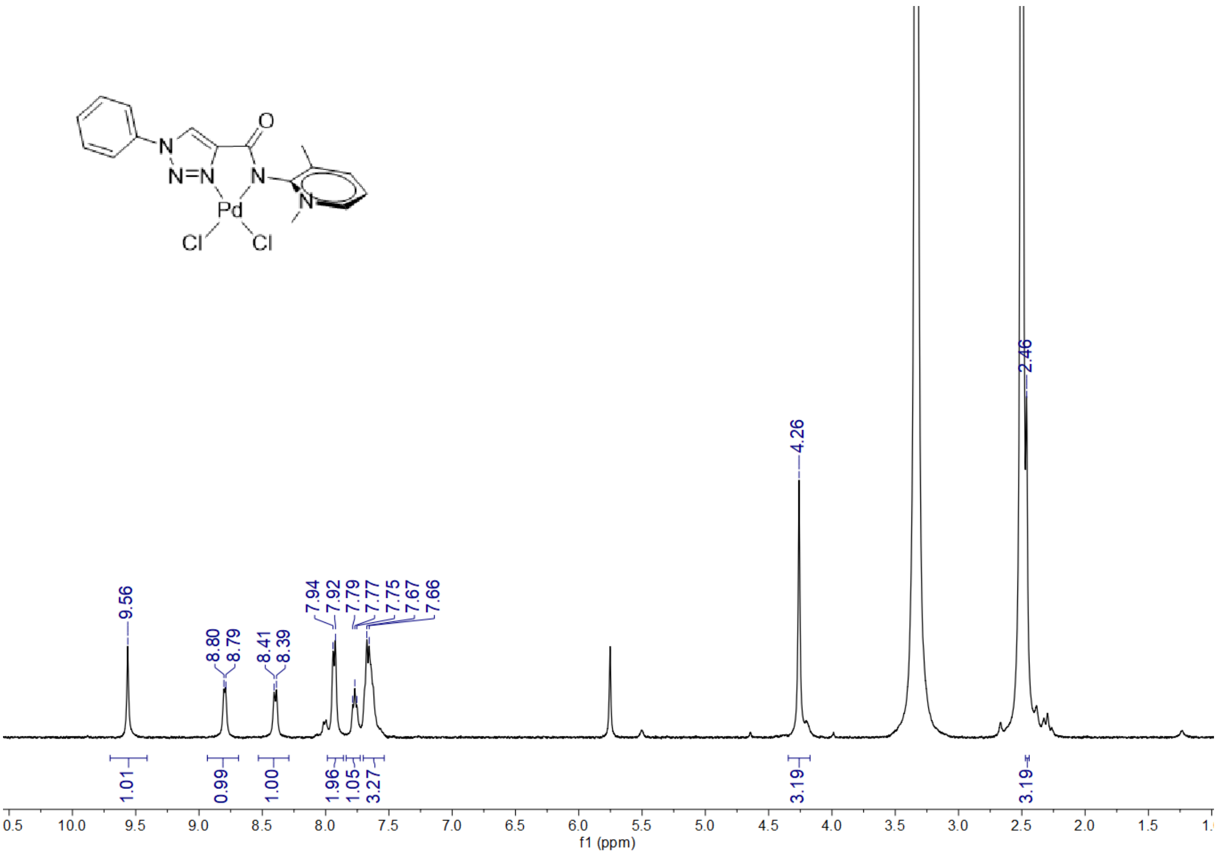

Figure S53: ^{1}H NMR spectrum (DMSO-*d*$_6$, 298 K, 400 MHz) of **5c**

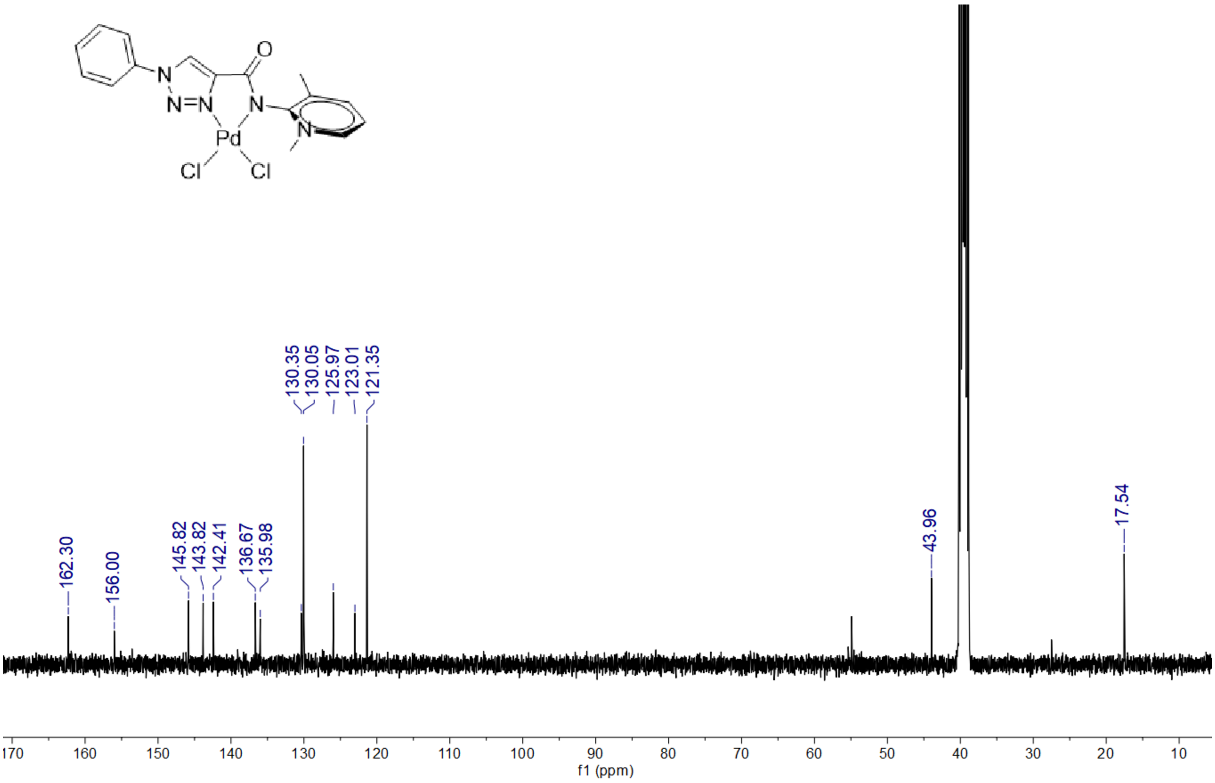

Figure S54: $^{13}C\{^{1}H\}$ NMR spectrum (DMSO-d_6, 298 K, 101 MHz) of **5c**

Synthesis of complex 5d

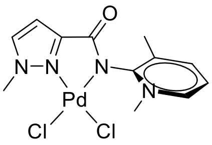

5d

Complex **5d** was prepared according to the general procedure IV from **4d** (76 mg, 0.33 mmol) and [PdCl$_2$(cod)] (89.85 mg, 0.314 mmol) and was isolated as an orange powder (120 mg, 93%). Crystals suitable for Xray diffraction were grown by slow diffusion of Et$_2$O into a MeOH solution of **5d**. ^{1}H NMR (400 MHz, CD$_2$Cl$_2$) δ = 8.16 - 8.09 (m, 2H, CH_{PYA}), 7.55 - 7.38 (m, 2H, CH_{PYA} + CH_{prz}), 6.65 (d, $^{3}J_{HH}$ = 2.7 Hz, 1H, CH_{prz}), 4.31 (s, 3H, N$_{PYA}$-CH_3), 4.23 (s, 3H, N$_{prz}$-CH_3), 2.57 (s, 3H, C-CH_3) ppm. $^{13}C\{^{1}H\}$ NMR (101 MHz, CD$_2$Cl$_2$, 298K) δ = 164.97 (*C*O), 149.19 (*C*$_{PYA}$), 145.63 (*C*H$_{PYA}$), 140.69 (*C*H$_{PYA}$), 139.52 (*C*$_{PYA}$-Me), 135.22 (*C*H$_{prz}$), 122.68 (*C*H$_{PYA}$), 106.83 (*C*H$_{prz}$), 45.41 (N$_{PYA}$*C*H$_3$), 41.62 (N$_{prz}$*C*H$_3$), 18.72 (C-*C*H$_3$) ppm. HR-MS (m/z) calculated for $C_{14}H_{17}ClN_5OPd$ [M-Cl+CH$_3$CN]$^+$ = 412.0156; found: 412.0155. Elemental Analysis calculated for $C_{12}H_{14}Cl_2N_4OPd$ x 0.25 CH_2Cl_2: C 34.31, H 3.41, N 13.07%; found: C 34.57, H 3.48, N 12.78.

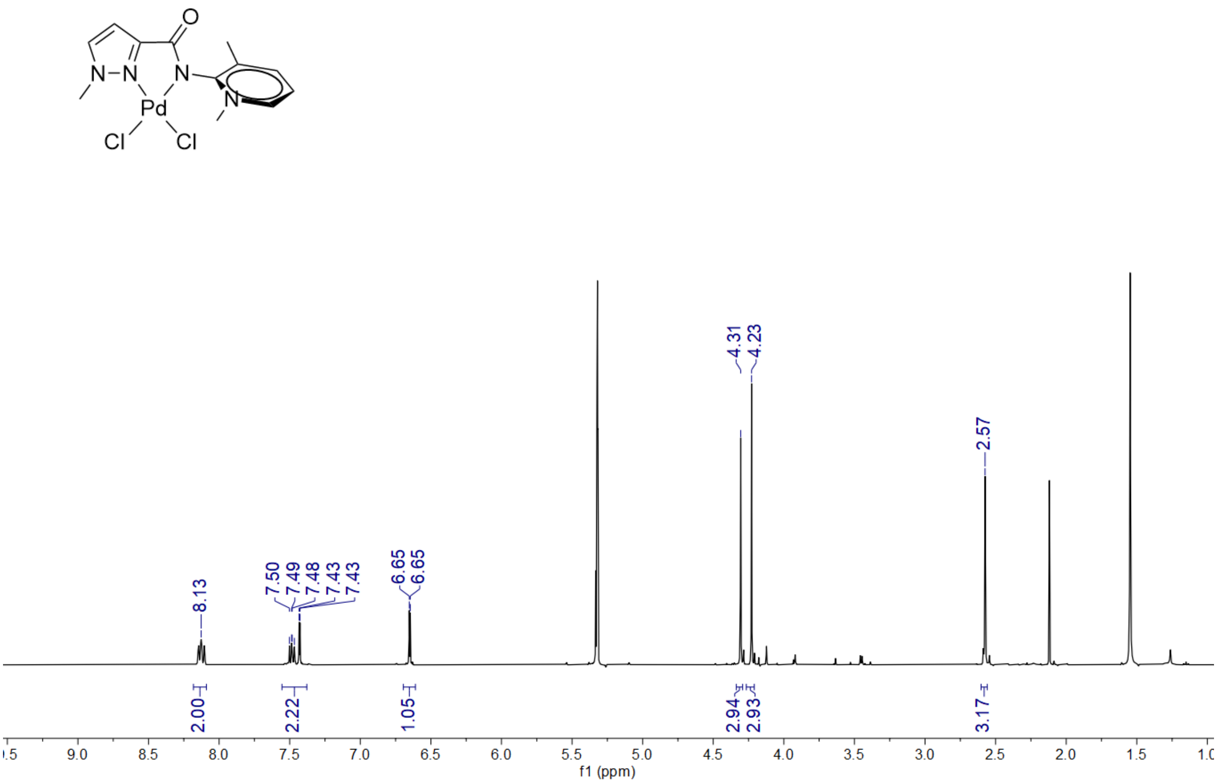

Figure S55: ^{1}H NMR spectrum (CD_2Cl_2, 298 K, 400 MHz) of **5d**

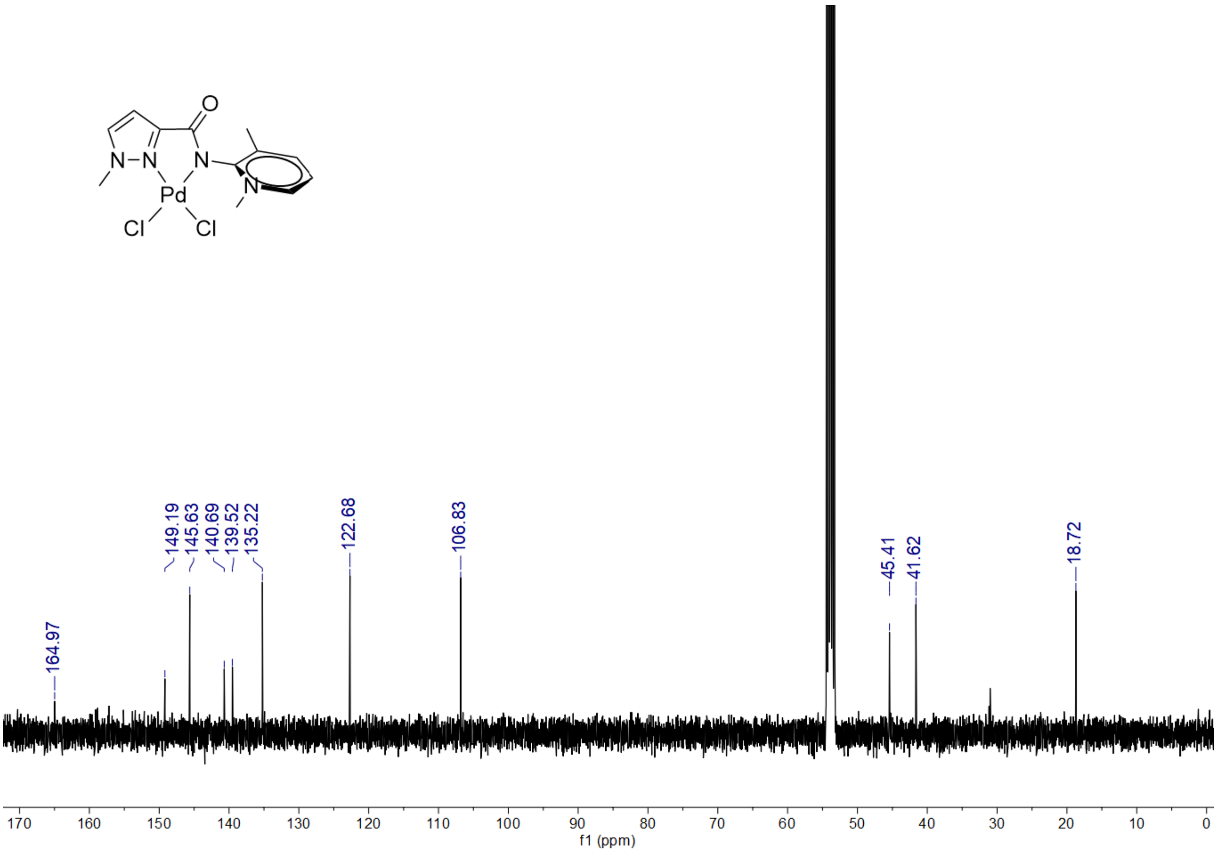

Figure S56: $^{13}C\{^{1}H\}$ NMR spectrum (CD_2Cl_2, 298 K, 101 MHz) of **5d**

Synthesis of complex 5e

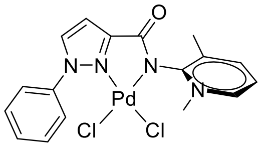

5e

Complex **5e** was prepared according to the general procedure IV from **4e** (80.2 mg, 0.27 mmol) and [PdCl$_2$(cod)] (74.3 mg, 0.26 mmol) and was obtained as a yellow powder (113 mg, 93%). ^{1}H NMR (400 MHz, CD$_2$Cl$_2$) δ = 8.16 (d, $^3J_{HH}$ = 6.3 Hz, 1H, CH_{PYA}), 8.12 (d, $^3J_{HH}$ = 7.7 Hz, 1H, CH_{PYA}), 7.69 (d, $^3J_{HH}$ = 2.7 Hz, 1H, CH_{prz}), 7.59-7.46 (m, 6H, CH_{Phe} + CH_{PYA}), 6.87 (d, $^3J_{HH}$ = 2.7 Hz, 1H, CH_{prz}), 4.35 (s, 3H, N$_{PYA}$CH_3), 2.62 (s, 3H, C$_{PYA}$CH_3) ppm. ^{13}C{^{1}H} NMR (101 MHz, CD$_2$Cl$_2$, 298K) δ = 164.65 (*C*O), 158.61 (C_{PYA}), 151.11 (C_{prz}), 145.63 (CH$_{PYA}$), 140.77 (CH$_{PYA}$), 139.36 (C_{Phe}), 138.66 (C_{PYA}-Me), 136.29 (CH$_{prz}$), 130.33 (CH$_{Phe}$), 129.23 (CH$_{Phe}$), 126.72 (CH$_{Phe}$), 122.63 (CH$_{PYA}$), 107.67 (CH$_{prz}$), 45.48 (N$_{PYA}$$CH_3$), 18.81 (C$_{PYA}$$CH_3$) ppm. HR-MS (m/z) calculated for $C_{21}H_{19}ClN_5OPd$ [M-Cl+CH$_3$CN]$^+$ = 474.0313; found: 474.0305. Elemental Analysis calculated for $C_{17}H_{16}Cl_2N_4OPd$: C 44.44, H 3.89, N 11.39%; found: C 44.68, H 3.64, N 11.30%.

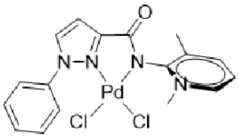

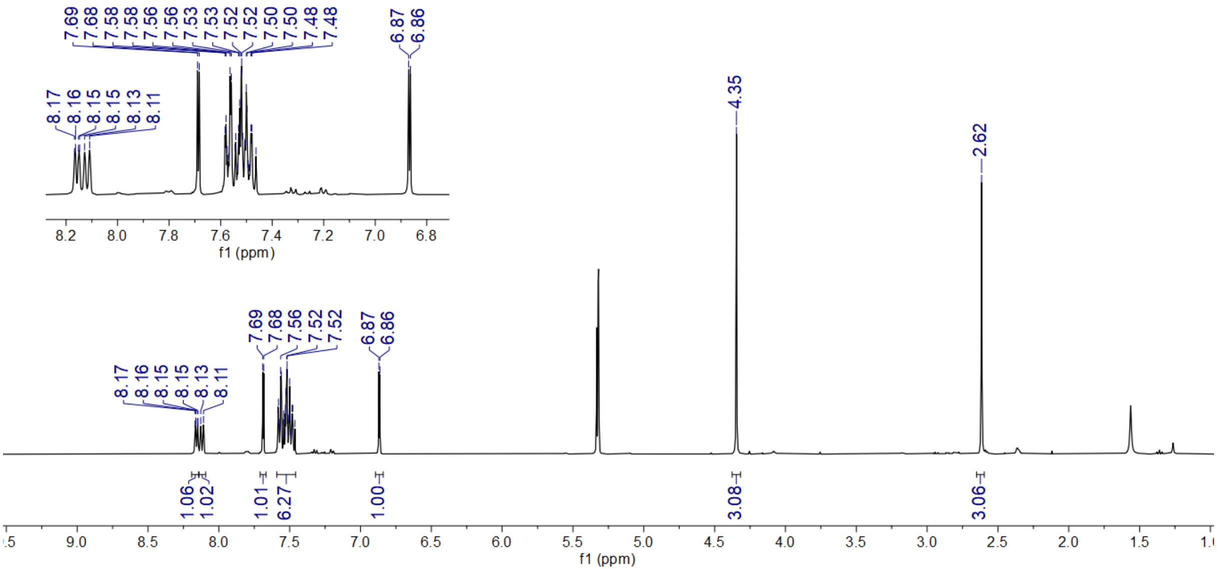

Figure S57: ^{1}H NMR spectrum (CD$_2$Cl$_2$, 298 K, 400 MHz) of **5e**

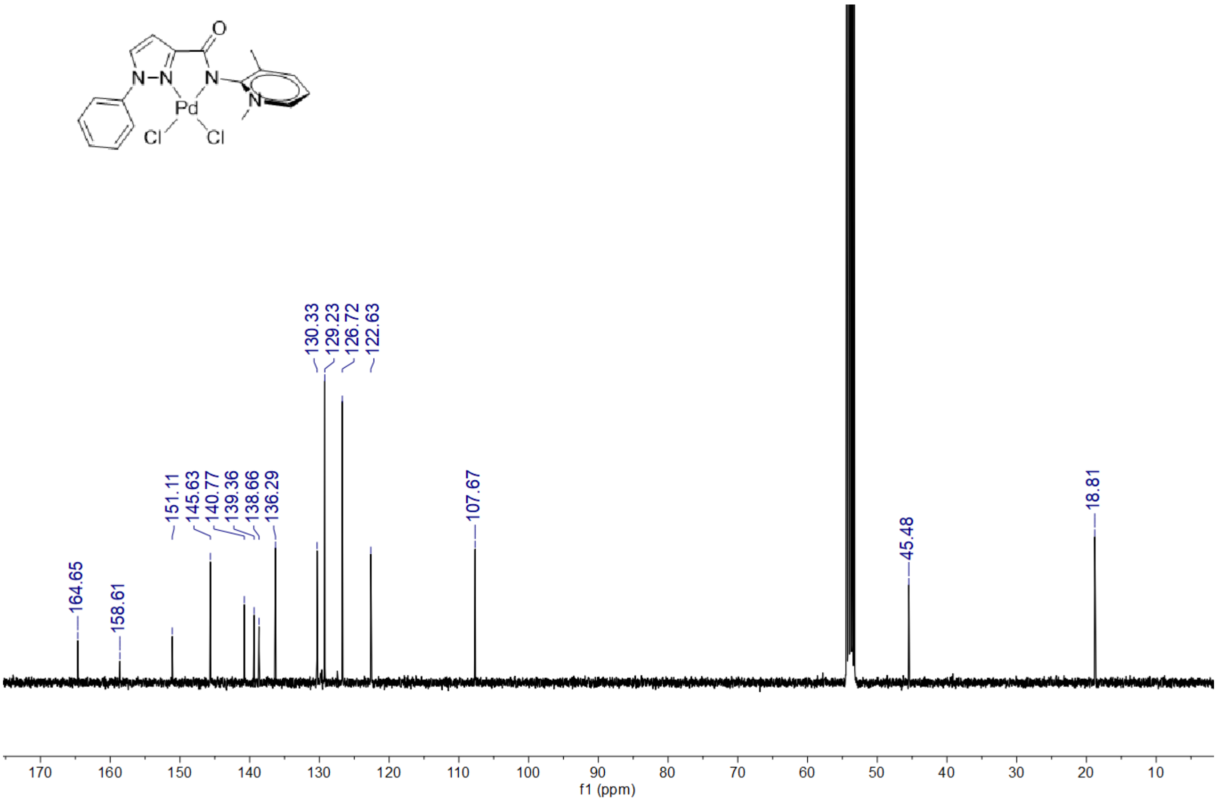

Figure S58: $^{13}C\{^{1}H\}$ NMR spectrum (CD_2Cl_2, 298 K, 101 MHz) of **5e**

Synthesis of complex 5f

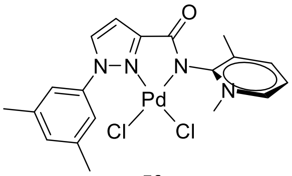

5f

Complex **5f** was prepared according to the general procedure IV from ligand **4f** (90.3 mg, 0.28 mmol) and [$PdCl_2$(cod)] (76.3 mg, 0.267 mmol) and was isolated as a yellow powder (130 mg, 97%). Crystals suitable for Xray diffraction were grown by slow diffusion of Et_2O into a CH_2Cl_2 solution of **5f**. ^{1}H NMR (400 MHz, CD_2Cl_2) δ = 8.24 – 8.05 (m, 2H, CH_{PYA}), 7.66 (s, 1H, CH_{prz}), 7.49 (m, 1H, CH_{PYA}), 7.17 (m, 3H, CH_{Phe}), 6.84 (s, 1H, CH_{prz}), 4.34 (s, 3H, $N_{PYA}CH_3$), 2.61 (s, 3H, $C_{PYA}CH_3$), 2.40 (s, 6H, Phe-CH_3) ppm. $^{13}C\{^{1}H\}$ NMR (101 MHz, CD_2Cl_2, 298K) δ = 164.32 (*C*O), 158.26 (C_{PYA}), 150.48 (C_{prz}), 145.18 (CH_{PYA}), 140.39 (CH_{PYA}), 138.95 (C_{Phe}), 138.71 (Phe-*C*CH_3), 137.85 (C_{PYA}-Me), 135.64 (CH_{prz}), 131.28 (CH_{Phe}), 123.78 (CH_{Phe}), 122.20 (CH_{PYA}), 107.12 (CH_{prz}), 45.03 ($N_{PYA}CH_3$), 20.98 (Phe-CH_3), 18.38 (C_{PYA}-CH_3) ppm. HR-MS (m/z) calculated for $C_{23}H_{23}ClN_5OPd$ [M-Cl+CH_3CN]$^{+}$ = 502.0626; found: 502.0612. Elemental Analysis calculated for $C_{19}H_{20}Cl_2N_4OPd$ x 0.25 CH_2Cl_2: C 44.55, H 3.98, N 10.80%; found: C 44.85, H 3.87, N 10.33%.

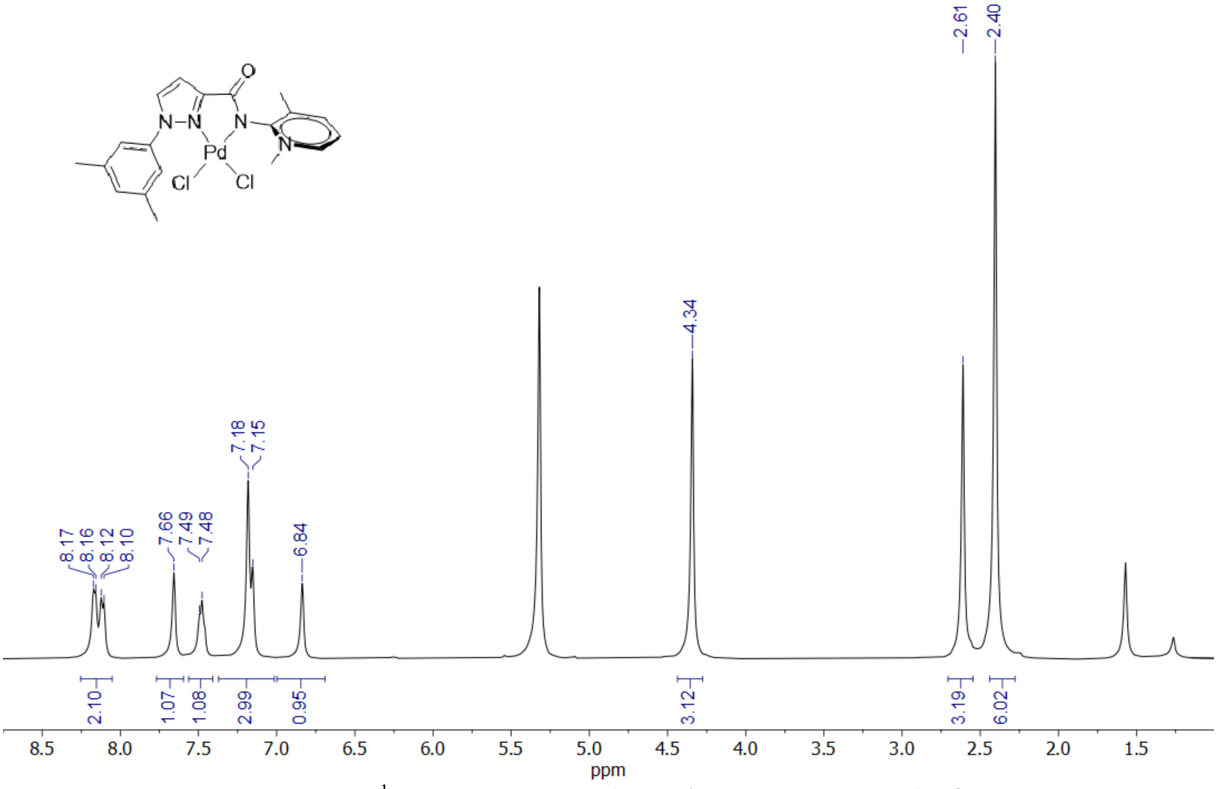

Figure S59: ^{1}H NMR spectrum (CD_2Cl_2, 298 K, 400 MHz) of **5f**

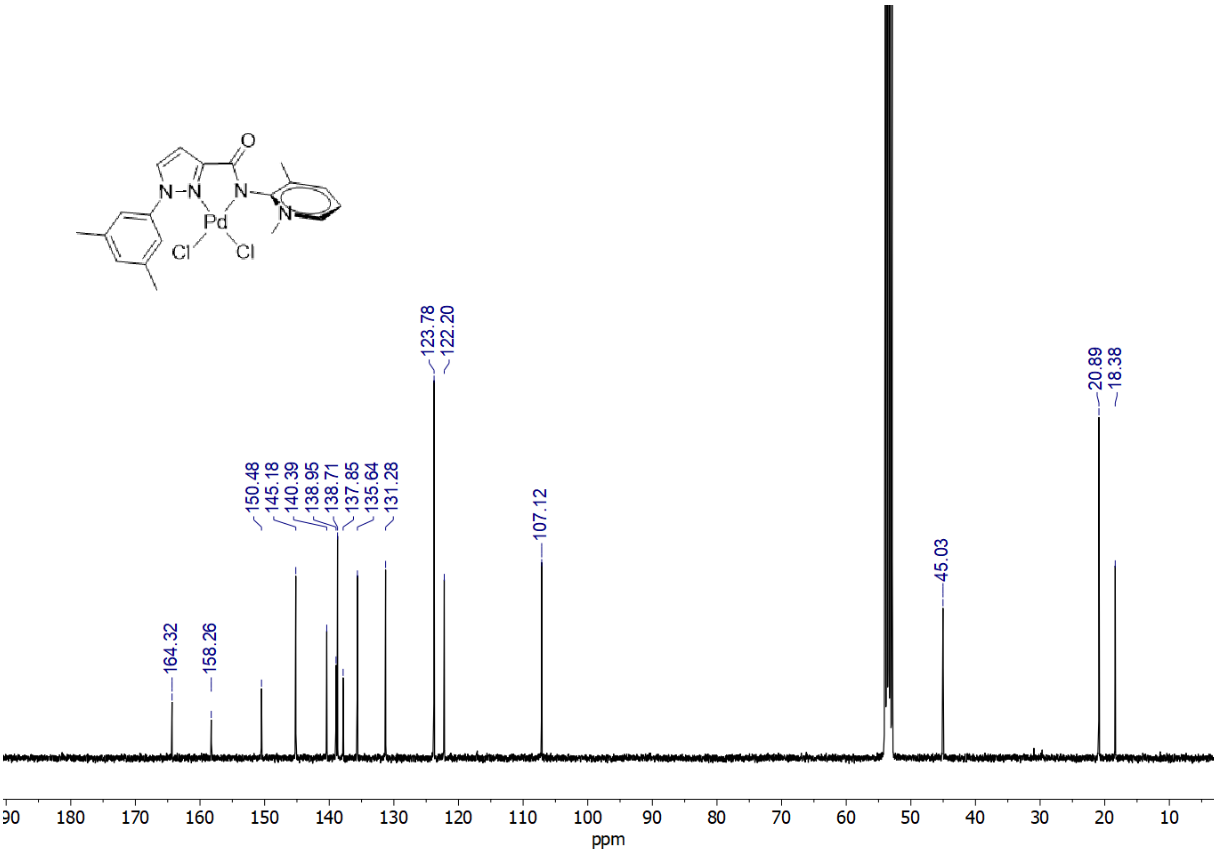

Figure S60: ^{13}C{^{1}H} NMR spectrum (CD_2Cl_2, 298 K, 101 MHz) of **5f**

Synthesis of heteroaryl-PYA Pd complex 5g

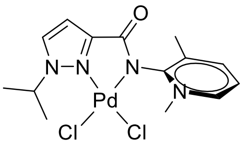

5g

Complex **5g** was prepared according to the general procedure IV from ligand **4g** (101.1 mg, 0.387 mmol) and [PdCl$_2$(cod)] (106.1 mg, 0.37 mmol) and was obtained as a yellow powder (134.2 mg, 84%). Crystals suitable for Xray diffraction were grown by slow diffusion of Et$_2$O into a CH$_2$Cl$_2$ solution of **5g**. ^{1}H NMR (400 MHz, CD$_2$Cl$_2$) δ = 8.21 - 8.03 (m, 2H, CH_{PYA}), 7.58 (d, $^3J_{HH}$ = 2.7 Hz, 1H, CH_{prz}), 7.48 (apparent triplet, 1H, CH_{PYA}), 6.69 (d, $^3J_{HH}$ = 2.7 Hz, 1H, CH_{prz}), 5.96 (hept, $^3J_{HH}$ = 6.7 Hz, 1H, N$_{\text{prz}}$-CH), 4.31 (s, 3H, N$_{\text{PYA}}$-CH_3), 2.58 (s, 3H, C-CH_3), 1.50 (d, $^3J_{HH}$ = 1.0 Hz, 6H, N$_{\text{prz}}$-C-CH_3) ppm. ^{13}C{^{1}H} NMR (101 MHz, CD$_2$Cl$_2$, 298K) δ = 165.04 (CO), 158.88 (C_{PYA}), 148.42 (CH$_{\text{PYA}}$), 145.56 (CH$_{\text{PYA}}$), 140.69 (C_{PYA}-Me), 139.57 (CH$_{\text{PYA}}$), 129.93 (CH$_{\text{prz}}$), 122.66 (CH$_{\text{PYA}}$), 107.09 (CH$_{\text{prz}}$), 54.68 (N$_{\text{prz}}C$H), 45.41 (N$_{\text{PYA}}C$H$_3$), 23.47 (N$_{\text{prz}}$-C-CH$_3$), 23.33 (N$_{\text{prz}}$-C-CH$_3$), 18.75 (C-CH$_3$) ppm. HR-MS (m/z) calculated for $C_{14}H_{18}ClN_4OPd$ [M-Cl]$^+$ = 399.0204; found: 399.0205. Elemental Analysis calculated for $C_{14}H_{18}Cl_2N_4OPd$: C 38.60, H 4.16, N 12.86%; found: C 38.90, H 4.19, N 12.54%.

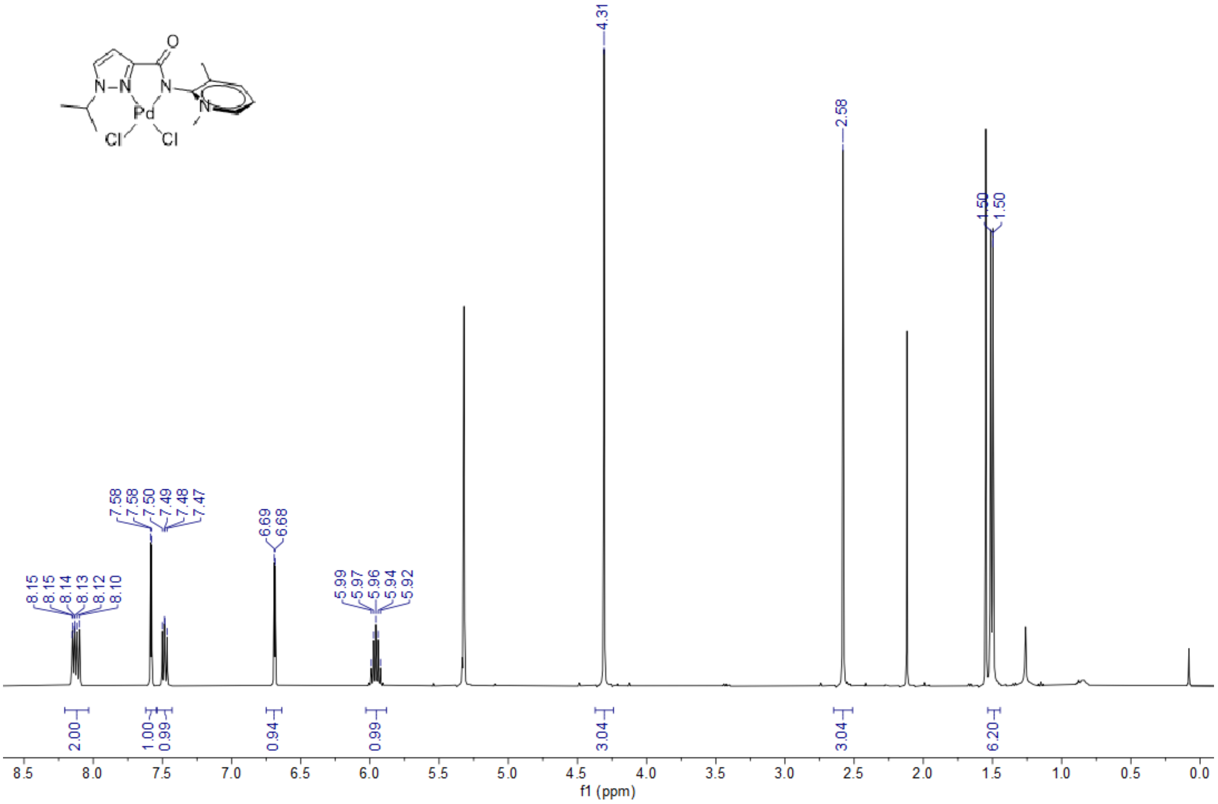

Figure S61: ^{1}H NMR spectrum (DMSO-d_6, 298 K, 400 MHz) of **5g**

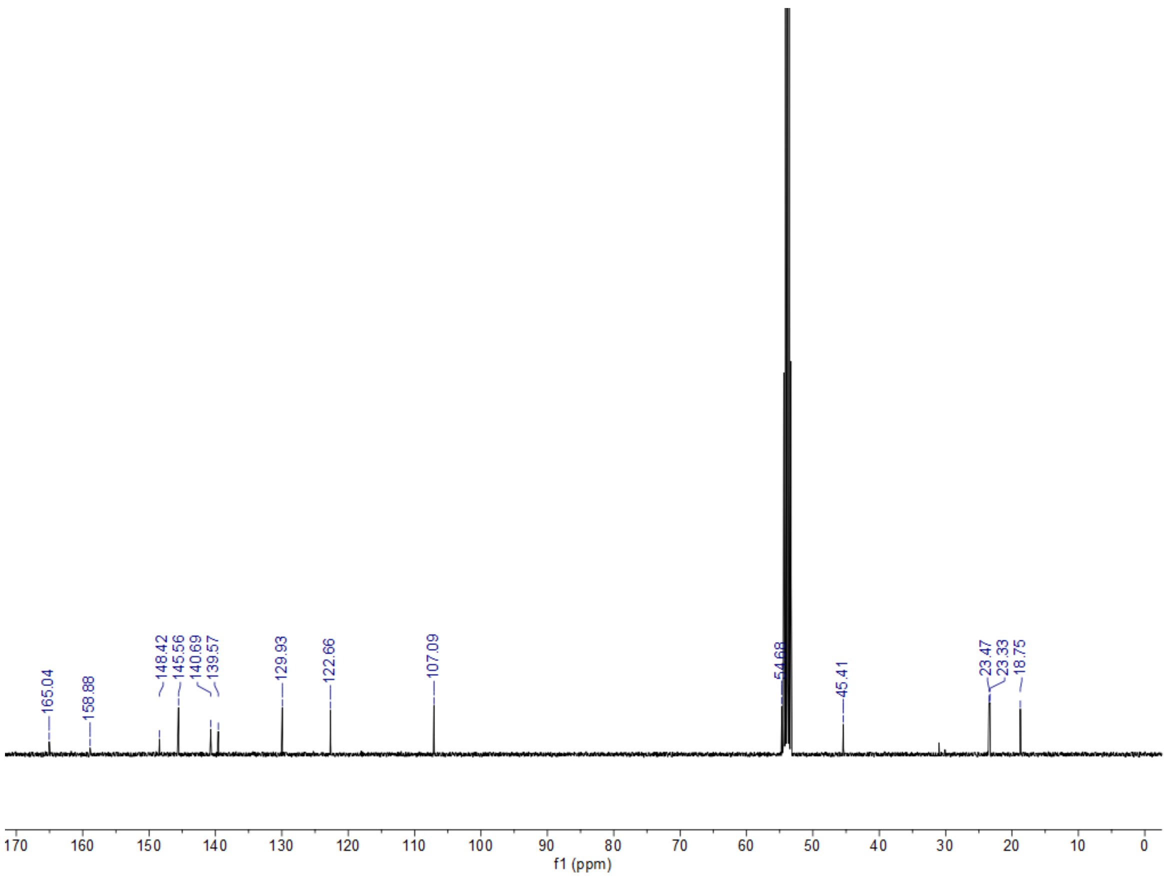

Figure S62: $^{13}C\{^{1}H\}$ NMR spectrum (DMSO-d_6, 298 K, 101 MHz) of **5g**

S.2 Crystal structure determinations

Crystals of **5b**, **5d, 5f** and **5g** immersed in parabar oil were mounted at ambient conditions and transferred into the stream of nitrogen (173 K). All measurements were made on a *RIGAKU XtaLAB Synergy R*, HyPix-Arc 100 area-detector diffractometer[S8] using mirror optics monochromated Mo *K*α radiation (λ = 0.71073 Å). Data reduction was performed using the *CrysAlisPro*[S8] program. The intensities were corrected for Lorentz and polarization effects, and a numerical absorption correction based on gaussian integration over a multifaceted crystal model with additional empirical absorption correction using spherical harmonics using SCALE3 ABSPACK in *CrysAlisPro*[S8] was applied. All the structures were solved by intrinsic phasing using *SHELXT,*[S9] which revealed the positions of all non-hydrogen atoms of the title compound. All non-hydrogen atoms were refined anisotropically. H-atoms were assigned in geometrically calculated positions and refined using a riding model where each H-atom was assigned a fixed isotropic displacement parameter with a value equal to 1.2Ueq of its parent atom (1.5Ueq for methyl groups). Refinement of the structure was carried out on F^2 using full-matrix least-squares procedures, which minimized the function $\Sigma w(F_o^2 - F_c^2)^2$. The weighting scheme was based on counting statistics and included a factor to downweight the intense reflections. All calculations were performed using the *SHELXL-2014/7*[S10] program in OLEX2.[S11] For complex **5d**, areas containing disorder solvents were found where a satisfactory solvent model might not be achieved; therefore, a solvent mask was used to include the contribution of electron density found in void areas into the calculated structure factor. The total number of electrons found in the void areas is shown in Table S3. For complex **5g**, a disorder model was used for parts of the structure where the occupancies of each disorder component were refined through the use of a free variable. The sum of equivalent components was constrained to 100%. The structure was refined as a two components inversion twin. Data collection and refinement parameters for **5b, 5d, 5g** and **5f** are given in Tables S1–S5. Crystallographic data for all structures reported in this paper have been deposited with the Cambridge Crystallographic Data Centre (CCDC) as supplementary publication numbers 2394912–2394915.

Table S1. Crystal data and structure refinement for **5b**

CCDC No	2394912
Empirical formula	$C_{11}H_{11}Cl_2N_3O_2Pd$
Formula weight	394.53
Temperature/K	173.00(10)
Crystal system	monoclinic
Space group	I2/a
a/Å	19.6202(3)
b/Å	9.6407(2)
c/Å	14.9332(3)
α/°	90
β/°	91.131(2)

γ/°	90
Volume/Å^3	2824.10(9)
Z	8
ρ_{calc} g/cm^3	1.856
μ/mm^{-1}	1.692
F(000)	1552.0
Crystal size/mm^3	0.149 × 0.113 × 0.087
Radiation	Mo Kα (λ = 0.71073)
2Θ range for data collection/°	4.152 to 61.016
Index ranges	$-28 \leq h \leq 28$, $-13 \leq k \leq 13$, $-21 \leq l \leq 21$
Reflections collected	41482
Independent reflections	4316 [R_{int} = 0.0271, Rsigma = 0.0145]
Data/restraints/parameters	4316/0/216
Goodness-of-fit on F^2	1.107
Final R indexes [I>2σ (I)]	R1 = 0.0222, wR2 = 0.0584
Final R indexes [all data]	R1 = 0.0252, wR2 = 0.0595
Largest diff. peak/hole / e Å^{-3}	0.82/-0.51

Table S2. Crystal data and structure refinement for **5d**

CCDC No	2394913
Empirical formula	$C_{12}H_{14}Cl_2N_4OPd$
Formula weight	407.57
Temperature/K	173.00(10)
Crystal system	orthorhombic
Space group	Pccn
a/Å	10.97050(13)
b/Å	23.0363(2)
c/Å	14.07250(15)
α/°	90
β/°	90
γ/°	90
Volume/Å^3	3556.40(7)
Z	8
ρ_{calc} g/cm^3	1.522
μ/mm^{-1}	1.344
F(000)	1616.0
Crystal size/mm^3	0.314 × 0.224 × 0.184
Radiation	Mo Kα (λ = 0.71073)
2Θ range for data collection/°	4.112 to 61.012
Index ranges	$-15 \leq h \leq 15$, $-32 \leq k \leq 32$, $-20 \leq l \leq 20$
Reflections collected	102018
Independent reflections	5437 [Rint = 0.0262, Rsigma = 0.0091]
Data/restraints/parameters	5437/0/184
Goodness-of-fit on F^2	1.110
Final R indexes [I>2σ (I)]	R1 = 0.0227, wR2 = 0.0603
Final R indexes [all data]	R1 = 0.0247, wR2 = 0.0611
Largest diff. peak/hole / e Å^{-3}	0.52/-0.40

Table S3. Crystal data and structure refinement for **5f**

CCDC No	2394914
Empirical formula	$C_{19}H_{20}N_4OCl_2Pd$
Formula weight	497.69
Temperature/K	173.00(10)
Crystal system	monoclinic
Space group	P21/n
a/Å	11.62997(6)
b/Å	14.78476(7)
c/Å	11.92053(6)
α/°	90
β/°	105.0469(5)
γ/°	90
Volume/Å^3	1979.415(18)
Z	4
ρ_{calc} g/cm^3	1.670
μ/mm^{-1}	10.184
F(000)	1000.0
Crystal size/mm^3	0.146 × 0.124 × 0.07
Radiation	Cu Kα (λ = 1.54184)
2Θ range for data collection/°	9.466 to 147.46
Index ranges	-14 ≤ h ≤ 14, -18 ≤ k ≤ 18, -14 ≤ l ≤ 14
Reflections collected	39723
Independent reflections	4006 [Rint = 0.0381, Rsigma = 0.0136]
Data/restraints/parameters	4006/42/267
Goodness-of-fit on F^2	1.170
Final R indexes [I>2σ (I)]	R1 = 0.0237, wR2 = 0.0615
Final R indexes [all data]	R1 = 0.0238, wR2 = 0.0615
Largest diff. peak/hole / e Å^{-3}	0.43/-0.51

Table S4. Crystal data and structure refinement for **5g**

CCDC No	2394915
Empirical formula	$C_{14}H_{18}Cl_2N_4OPd$
Formula weight	435.62
Temperature/K	173.00(10)
Crystal system	orthorhombic
Space group	P212121
a/Å	8.91557(12)
b/Å	10.26115(13)
c/Å	18.9836(2)
α/°	90
β/°	90
γ/°	90
Volume/Å^3	1736.70(4)
Z	4
ρ_{calc} g/cm^3	1.666
μ/mm^{-1}	1.382

F(000)	872.0
Crystal size/mm^3	0.338 × 0.117 × 0.069
Radiation	Mo Kα (λ = 0.71073)
2Θ range for data collection/°	4.292 to 61.008
Index ranges	$-12 \leq h \leq 12$, $-14 \leq k \leq 14$, $-27 \leq l \leq 27$
Reflections collected	105258
Independent reflections	5302 [Rint = 0.0409, Rsigma = 0.0141]
Data/restraints/parameters	5302/226/328
Goodness-of-fit on F^2	1.109
Final R indexes [I>2σ (I)]	R1 = 0.0330, wR2 = 0.0763
Final R indexes [all data]	R1 = 0.0374, wR2 = 0.0781
Largest diff. peak/hole / e Å^{-3}	1.15/-0.43

Table S5. Solvent mask information for **5d**

Number	X	Y	Z	Volume	Electron count
1	-0.118	0.038	0.220	58.6	17.3
2	0.118	-0.038	0.780	58.6	16.4
3	-0.118	0.462	0.720	58.6	16.4
4	0.118	0.538	0.280	58.6	17.4
5	0.250	0.250	0.490	66.6	17.0
6	0.250	0.250	-0.010	66.6	17.9
7	0.382	0.538	0.780	58.6	17.1
8	0.382	0.962	0.280	58.6	16.6
9	0.618	0.038	0.720	58.6	17.2
10	0.618	0.462	0.220	58.6	16.7
11	0.750	0.750	0.510	66.6	17.8
12	0.750	0.750	0.010	66.6	17.0

S.3 Buried volume calculations

Buried volume calculations were carried out using the SambVca 2.1 Software.[S12] The calculations were performed using the following parameters: Bond radii scaled by 1.17x; Sphere radius of 3.5; Distance of the coordination point from the center of the sphere 0.0; Mesh spacing for numerical integration 0.10; H atoms were not included in the calculations.

Complex **5b**:

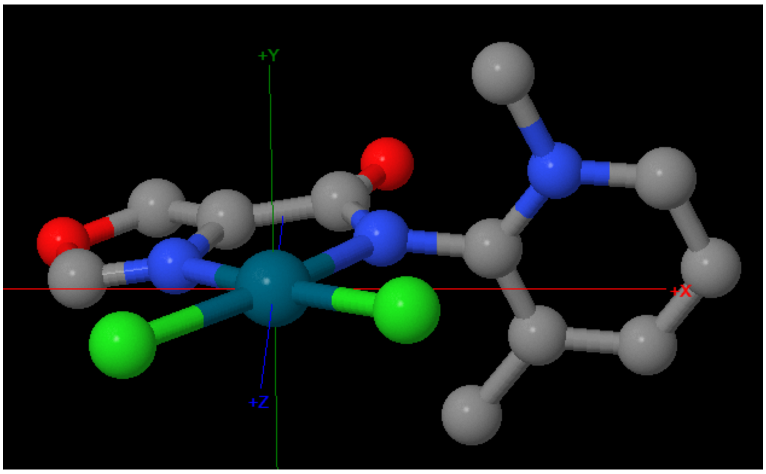

%V Free	%V Buried	% V Tot/V Ex
61.4	38.6	99.9

Quadrant	V f	V b	V t	%V f	%V b
SW	27.6	17.2	44.9	61.6	38.4
NW	32.3	12.6	44.9	71.9	28.1
NE	24.4	20.4	44.9	54.5	45.5
SE	25.8	19.1	44.9	57.5	42.5

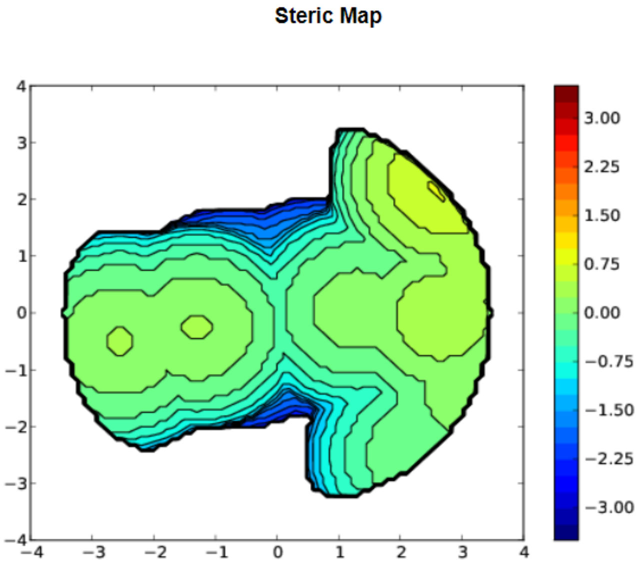

Complex **5d**:

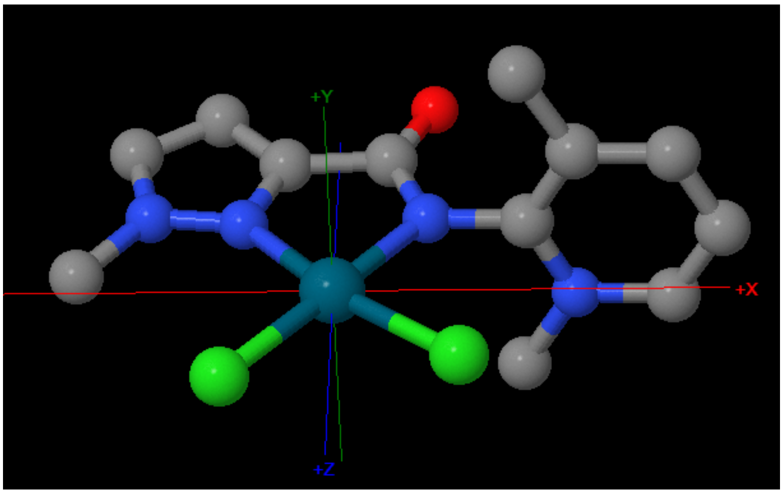

%V Free	%V Buried	% V Tot/V Ex
59.4	40.6	99.9

Quadrant	V f	V b	V t	%V f	%V b
SW	27.8	17.0	44.9	62.1	37.9
NW	30.1	14.8	44.9	67.1	32.9
NE	24.8	20.1	44.9	55.2	44.8
SE	23.9	21.0	44.9	53.3	46.7

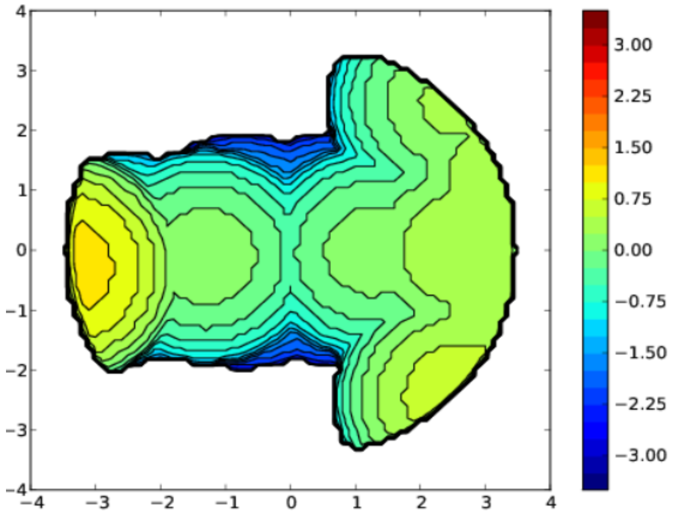

Complex **5f**:

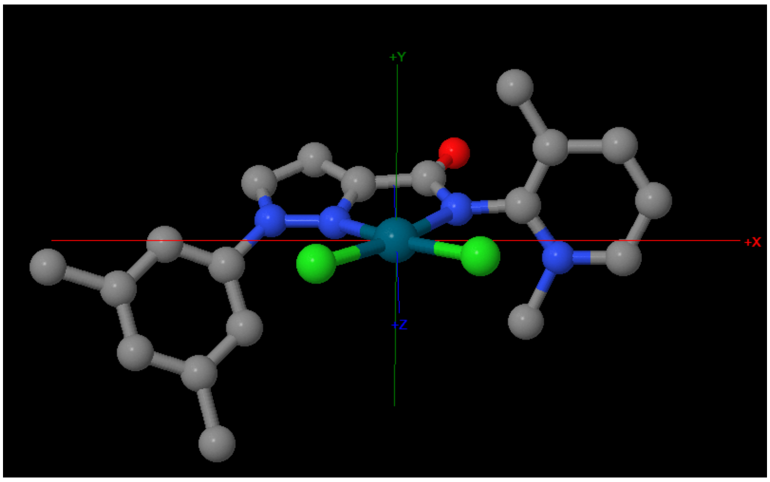

%V Free	%V Buried	% V Tot/V Ex
57.7	42.3	99.9

Quadrant	V f	V b	V t	%V f	%V b
SW	21.8	23.0	44.9	48.7	51.3
NW	32.6	12.3	44.9	72.6	27.4
NE	26.6	18.3	44.9	59.3	40.7
SE	22.6	22.2	44.9	50.4	49.6

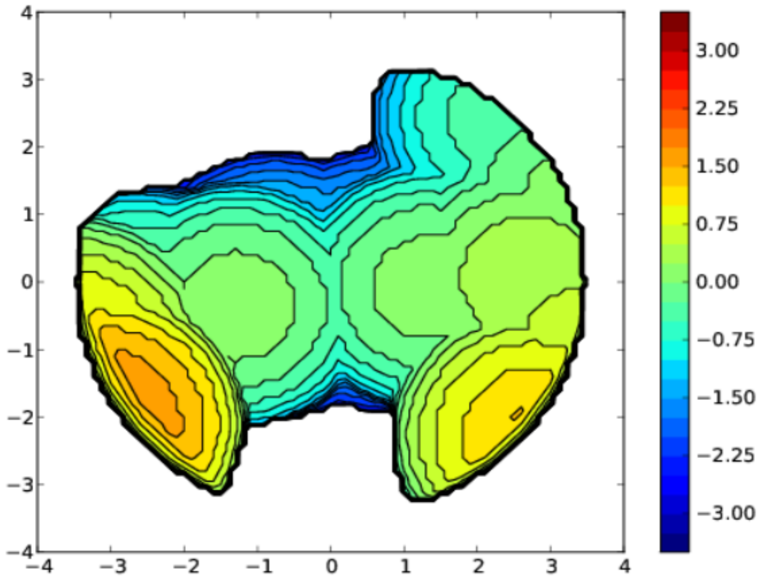

Complex **5g**:

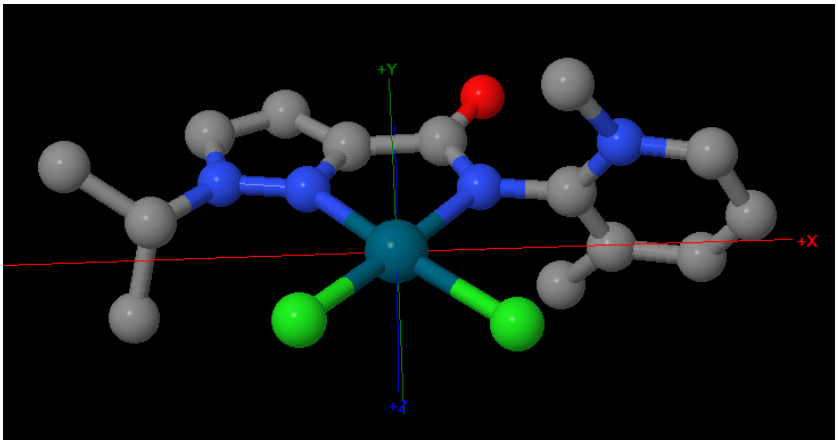

%V Free	%V Buried	% V Tot/V Ex
57.7	42.3	99.9

Quadrant	V f	V b	V t	%V f	%V b
SW	27.8	17.0	44.9	62.0	38.0
NW	28.1	16.8	44.9	62.5	37.5
NE	21.9	23.0	44.9	48.7	51.3
SE	25.8	19.0	44.9	57.6	42.4

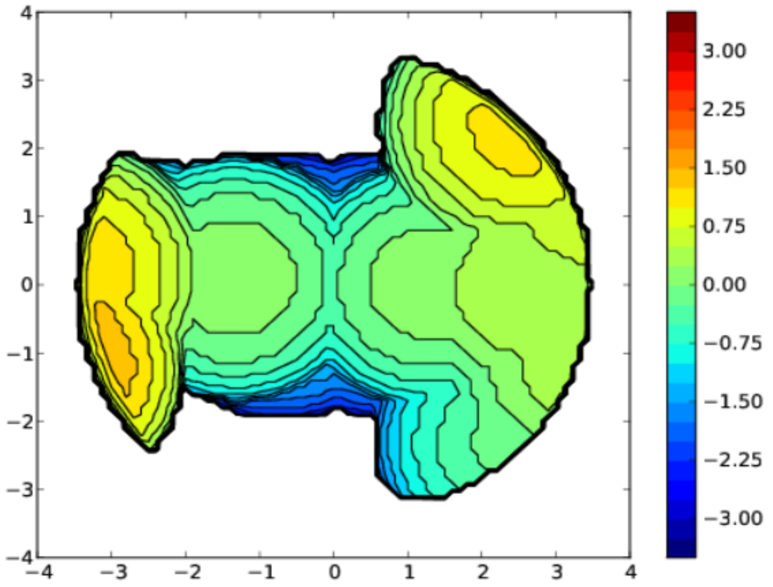

S.4 Catalytic procedures

S.4.1 General procedure for propiophenone α-arylation

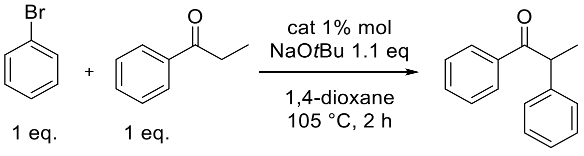

Scheme S6: General reaction conditions.

Complex **5a-5g** (0.01 mmol), hexamethylbenzene (0.2 mmol) and NaO*t*Bu (1.1 mmol) were placed in a 10 mL vial. The vial was closed, evacuated and backfilled with N_2 three times. 1,4-dioxane (1mL), propiophenone (1 mmol) and bromobenzene (1 mmol) were added. A sample was taken (time = 0) and the reaction mixture was heated to 105 °C. After 30 seconds, the reaction time was set to zero to account for the heating of the sample. Samples were taken after one and two hours, diluted in 1,4-dioxane, filtered over celite and analyzed by GC-FID.

S.4.2 Procedure for the acquisition of time-conversion profiles and TOF_{max} determination

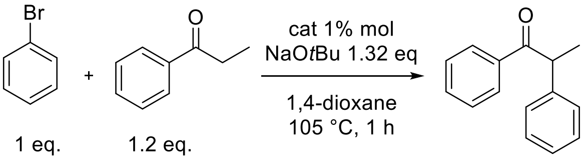

Scheme S7: General method for the kinetic profiling.

Complex **5a-5g** (0.01 mmol) were placed in a conical 5 mL vial that was closed, evacuated and backfilled with N_2 three times. Dry 1,4-dioxane (2 mL) and propiophenone (1.2 mmol) were added. In a second, normal shaped 10 mL vial, NaO*t*Bu (1.32 mmol) and the internal standard hexamethylbenzene (0.2 mmol) were weighted in and the vial was closed, evacuated and backfilled with N_2 three times. Dry 1,4-dioxane (2 mL) and bromobenzene (1 mmol) were added. Both solutions were pre-heated to 105 °C. From the aryl halide solution, the time zero sample was taken and diluted in a GC-vial in 1,4-dioxane. The complex solution was then transferred to the aryl halide solution via cannula to start the reaction. Aliquots were taken after given times, diluted in 1,4-dioxane, filtered over celite and analyzed by GC-FID.

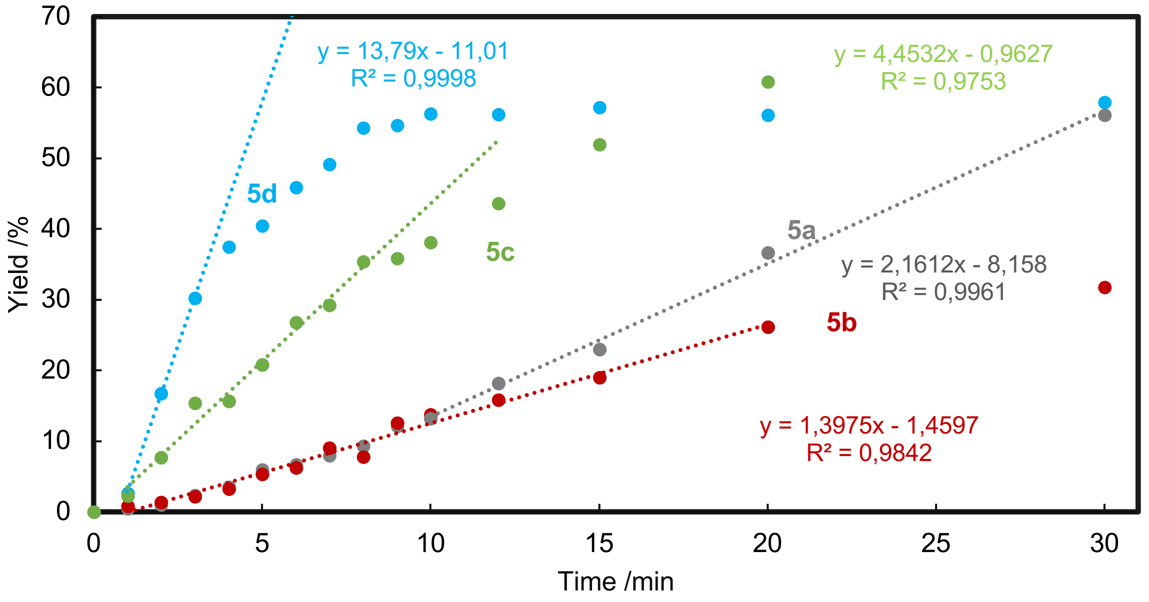

Figure S63: Time-conversion profile for the propiophenone α-arylation catalyzed by Pd complexes **5a-5d**. The linear regime considered for TOF_{max} evaluation is highlighted by the dotted lines for each catalyst. Slopes derived from these trendlines have been used for TOF_{max} calculation. Reaction conditions: PhBr (1.0 mmol), Propiophenone (1.2 mmol), [Pd] (0.01 mmol), NaO*t*Bu (1.32 mmol), 1,4-dioxane (2.0 mL) in a 10 mL vial, 105 °C under N_2, constant sampling was performed, yield determined by GC analysis using hexamethylbenzene as internal standard. All the experiments were run at least in duplicate.

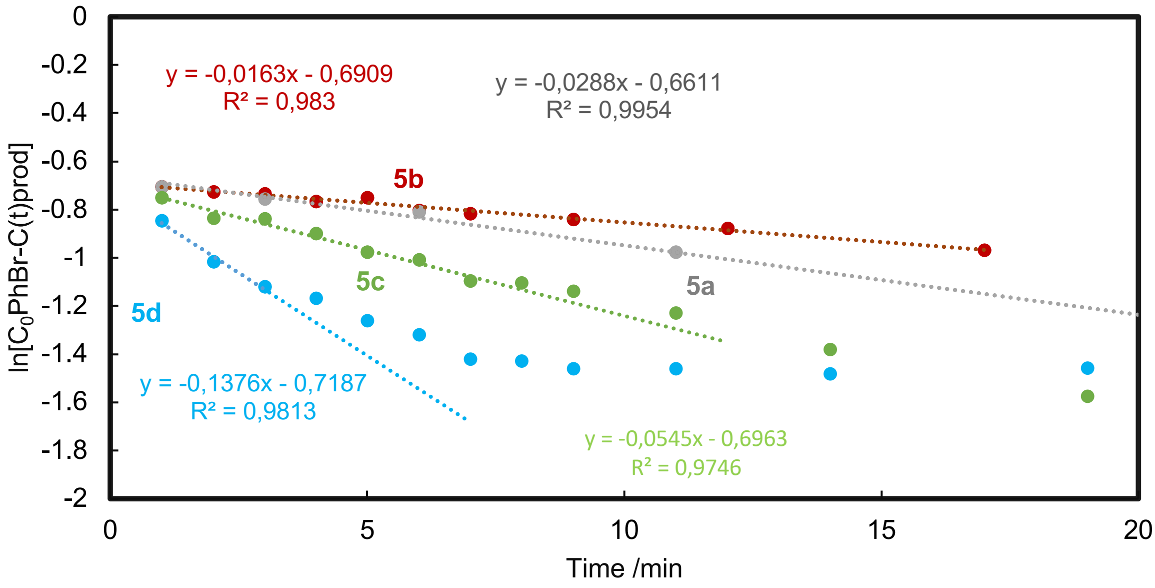

Figure S64: Linear regression analysis for the determination of the initial rate constant k_{ini} for the propiophenone α-arylation catalyzed by Pd complexes **5a-5d**. To mitigate mixing effects in the first minute of the reaction the conversion happening during the first minute of the reaction was not considered for catalyst activity analysis. Only points included in the linear regime at the maximal TOF were considered to calculate the slopes. Reaction conditions: PhBr (1.0 mmol), Propiophenone (1.2 mmol), [Pd] (0.01 mmol), NaO*t*Bu (1.32 mmol), 1,4-dioxane (2.0 mL) in a 10 mL vial, 105 °C under N_2, sampling as indicated in the graph, yield determined by GC analysis using hexamethylbenzene as internal standard.

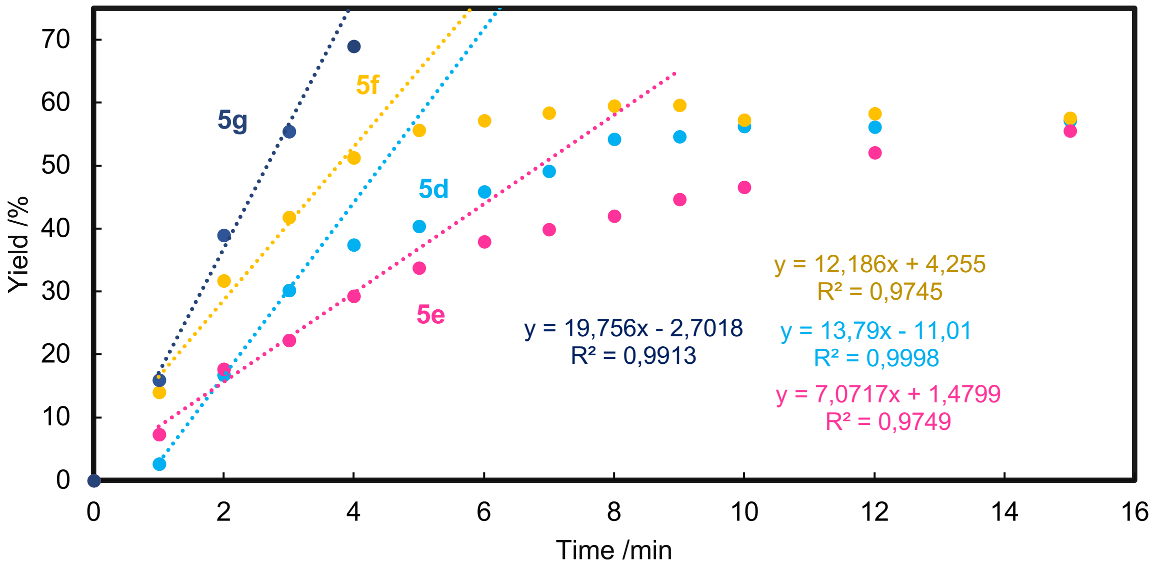

Figure S65: Time-conversion profile for propiophenone α-arylation catalyzed by Pd complexes **5d**–**5g**. The linear regime considered for TOF_{max} evaluation is highlighted by the dotted lines for each catalyst. Slopes derived from these trendlines have been used for TOF_{max} calculation. Reaction conditions: PhBr (1.0 mmol), Propiophenone (1.2 mmol), [Pd] (0.01 mmol), NaO*t*Bu (1.32 mmol), 1,4-dioxane (2.0 mL) in a 10 mL vial, 105 °C under N_2, constant sampling was performed, yields determined by GC analysis using hexamethylbenzene as internal standard. All experiments run at least in duplicate.

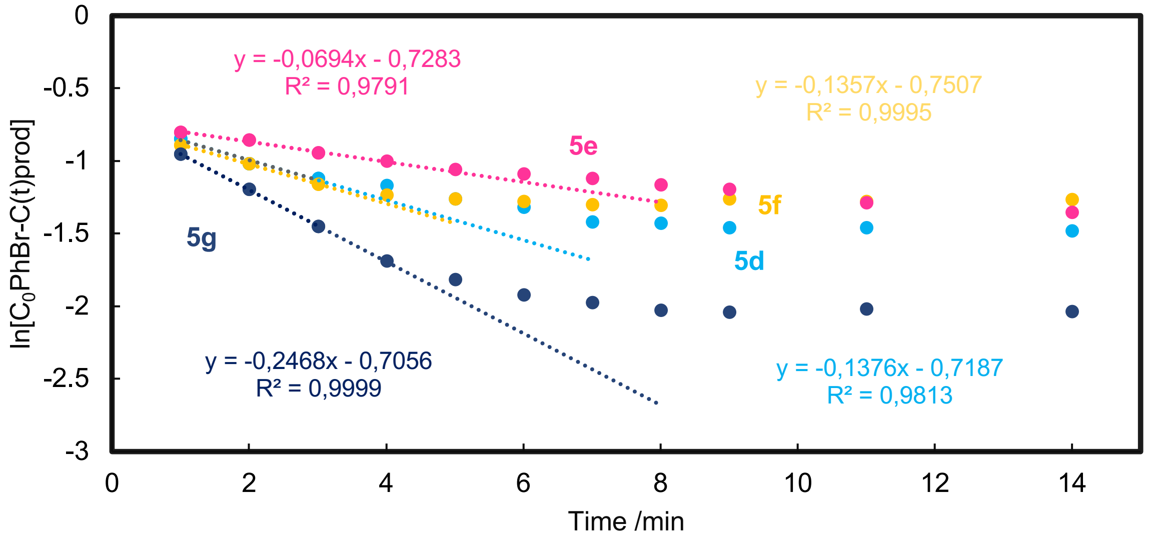

Figure S66: Linear regression analysis for the determination of the initial rate constant k_{ini} for the propiophenone α-arylation catalyzed by Pd complexes **5d-5g**. To mitigate mixing effects in the first minute of the reaction the conversion taking place during the first minute of the reaction was not considered for catalyst activity analysis. Only points included in the linear regime at the maximal TOF were considered to calculate the slopes. Reaction conditions: PhBr (1.0 mmol), Propiophenone (1.2 mmol), [Pd] (0.01 mmol), NaO*t*Bu (1.32 mmol), 1,4-dioxane (2.0 mL) in a 10 mL vial, 105 °C under N_2, constant sampling was performed, yield determined by GC analysis using hexamethylbenzene as internal standard.

S.4.3 General procedure for determining the order in palladium complex

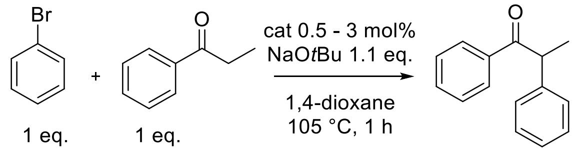

Scheme S8: General method for catalyst order determination.

Complex **5a** and **5d** (0.01 mmol), hexamethylbenzene (0.2 mmol) and NaO*t*Bu (1.1 mmol) were placed in a 10 mL vial and evacuated and backfilled with N_2. Dry 1,4-dioxane (2 mL), propiophenone (1 mmol) and bromobenzene (1 mmol) were added. A sample was taken (time = 0) and the reaction mixture was heated to 105 °C. After 30 seconds, the reaction time was set to zero to account for the heating of the sample. Aliquots were taken after given times, diluted in 1,4-dioxane, filtered over celite and analyzed with GC-FID. The reaction was repeated under exactly the same conditions at different catalyst loadings from 0.5 mol% to 3 mol% for **5d** and 0.5 mol% to 2 mol% for complex **5a**.

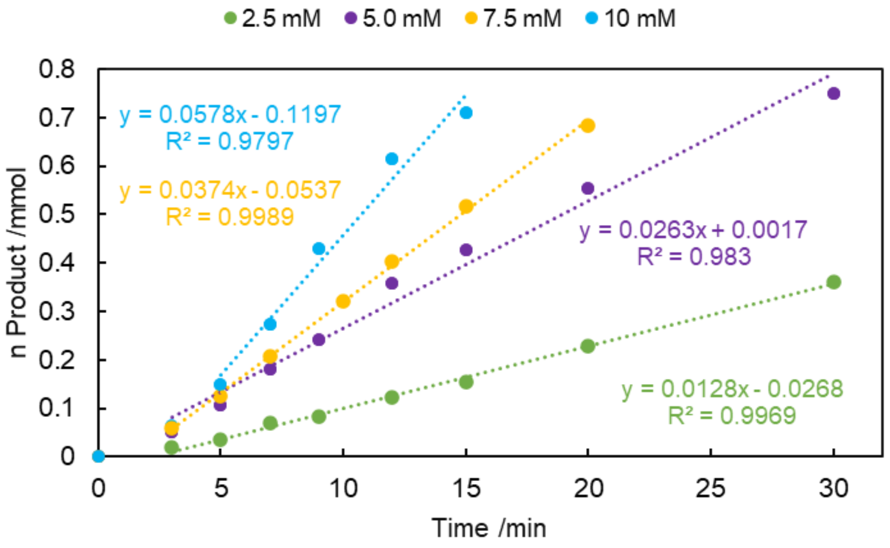

Figure S67: Time-conversion profile at different **5a** catalyst loadings in the linear regime, conditions see above.

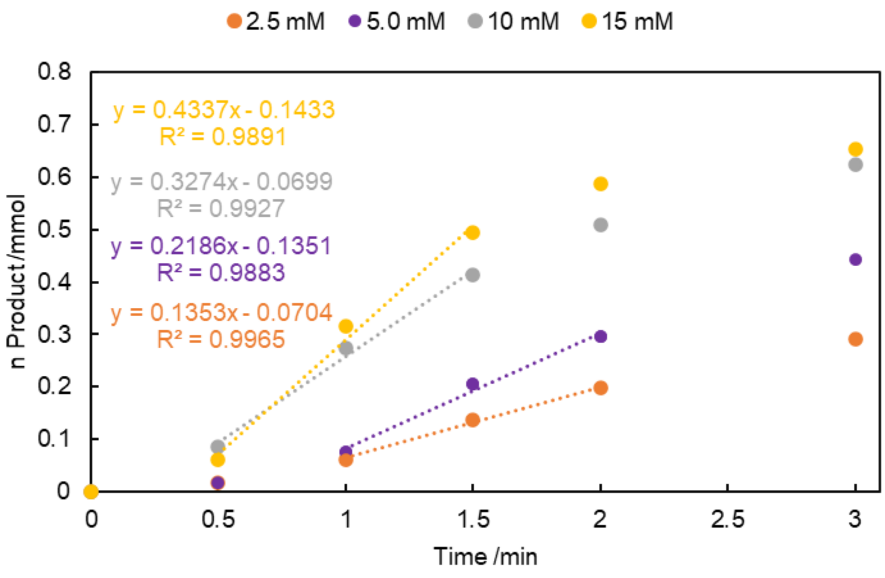

Figure S68: Time-conversion profile at different **5d** catalyst loadings in the linear regime, conditions see above.

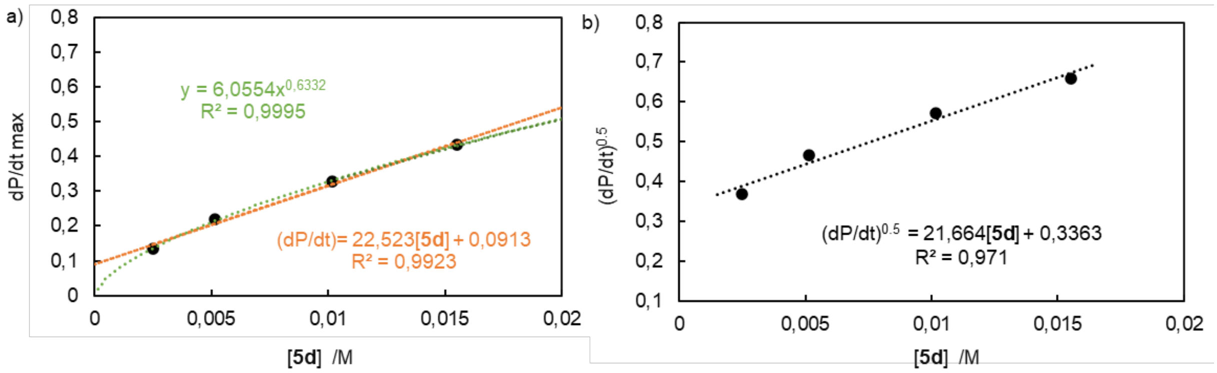

Figure S69: Determination of the catalytic rate order in **5d.** a) Evaluation of a 1st order rate dependency in **5d**. In orange the linear regression plot, in green the best power fit giving a catalyst rate order of 0,63. b) Linear plot for a half-order dependence in **5d** and the associated linear plot. Given the broken order calculated from the polynomial fit (in green) and the value obtain for the y-intercept, the possibility of a half-order dependence in **5d** was also explored through variable time normalization analysis (VTNA)[S13] kinetic studies (Fig. S70–S73) and a log-log plots (Fig. S74). While both the 1st order and ½ order models equally fit the data, no additional evidence supporting a half-order behavior in **5d** was found. Therefore, only the 1st order hypothesis was considered in subsequent discussions.

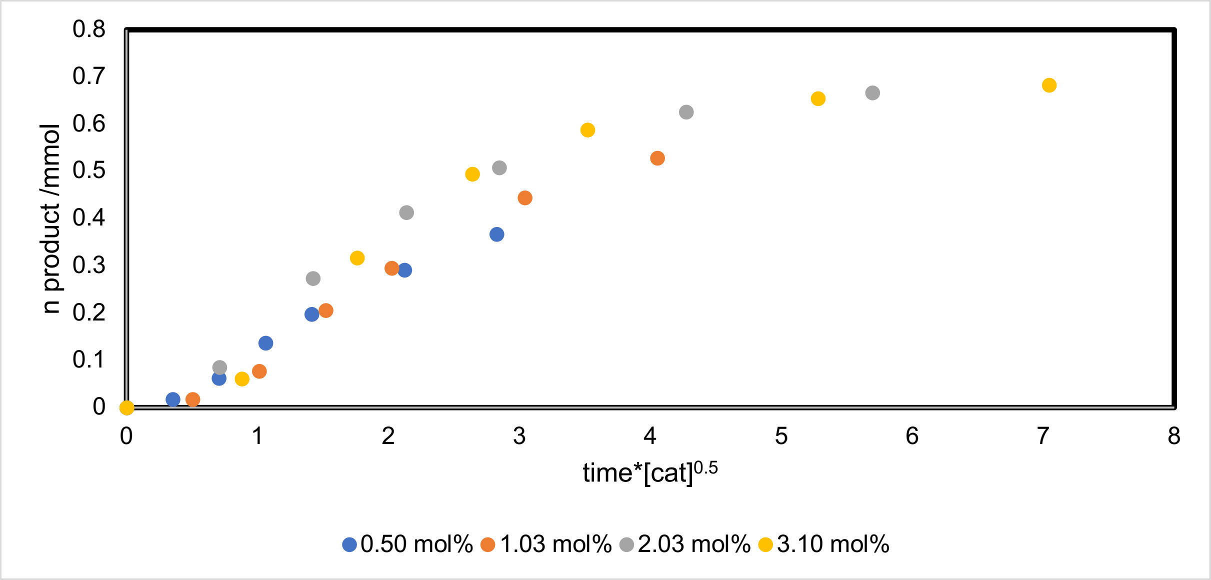

Figure S70: Catalyst rate order determination for **5d** using VTNA (ref S13) and assuming a 0.5 order in catalyst.

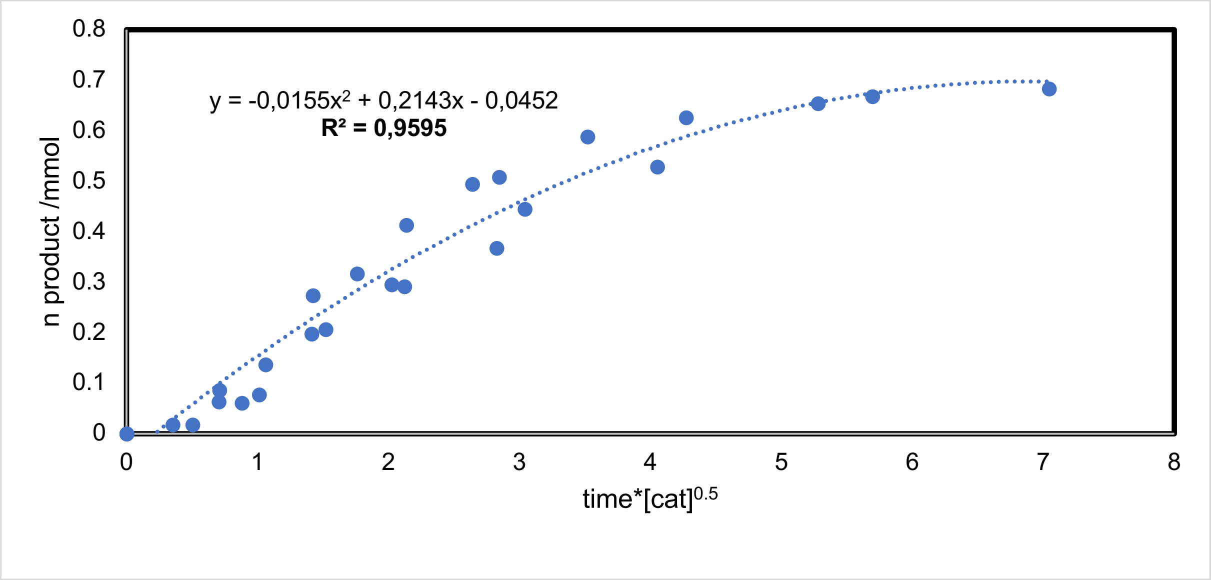

Figure S71: Catalyst rate order determination for **5d** using VTNA (ref S13) and assuming a 0.5 order in catalyst, in blue the overall best polynomial fit giving a $R^2 = 0.9595$.

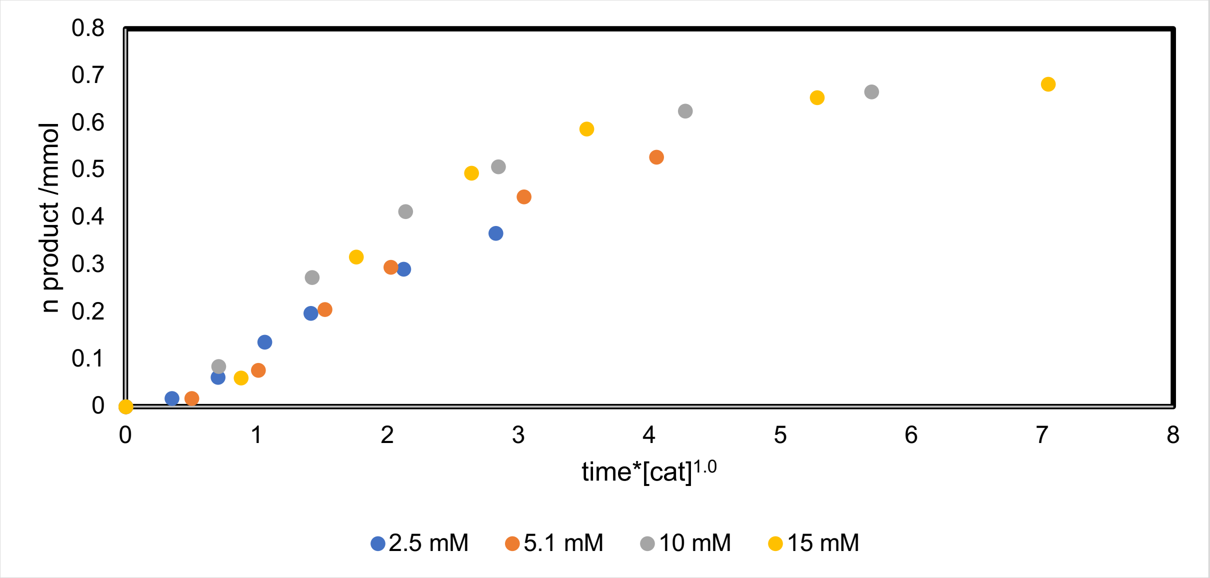

Figure S72: Catalyst rate order determination for **5d** using VTNA (ref S13) and assuming 1st order in catalyst.

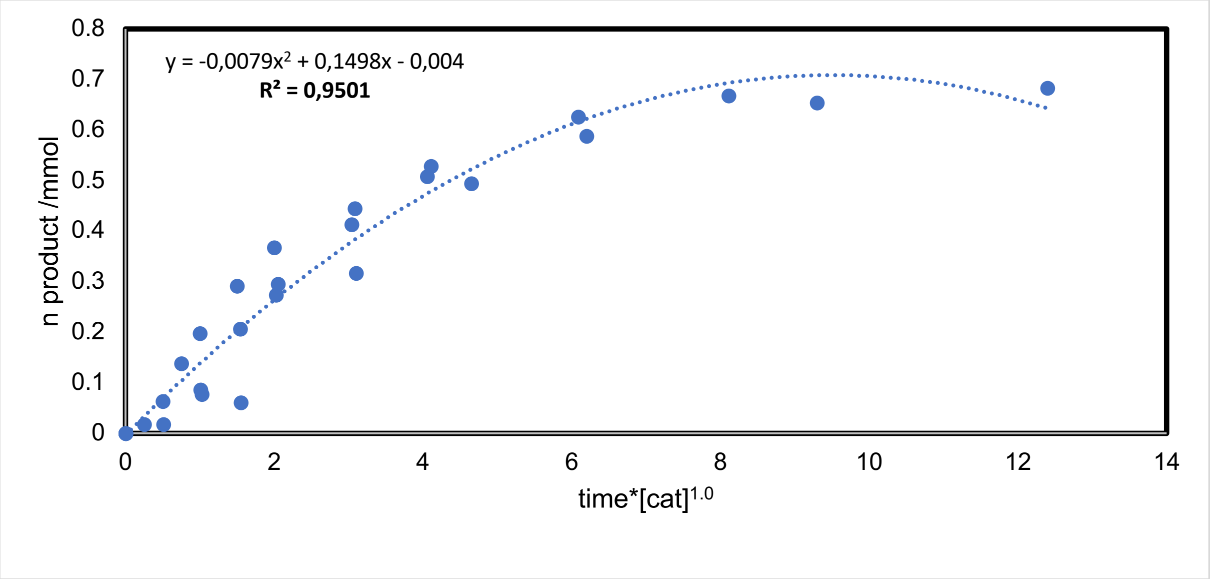

Figure S73: Catalyst rate order determination for **5d** using VTNA (ref S13) and 1st order in catalyst, in blue the overall best polynomial fit giving a $R^2 = 0.9501$.

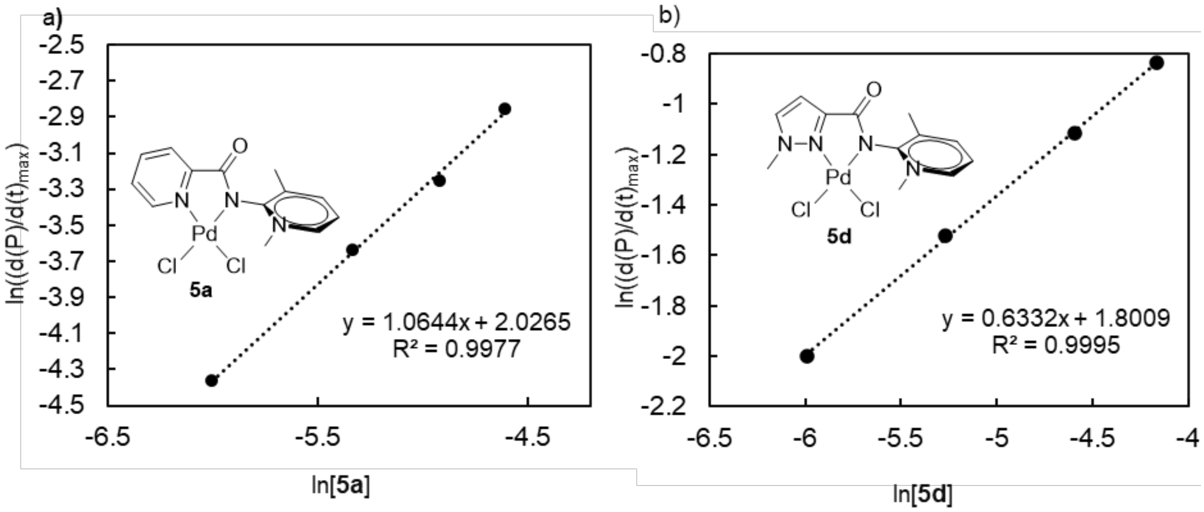

Figure S74: Log-log plots for determining the catalyst rate order revealing a) a slope of 1 and first order dependence in [**5a**]; b) a slope of 0.63 and a broken order dependence in [**5d**].

S.4.4 Procedure for determining the rate order in substrates

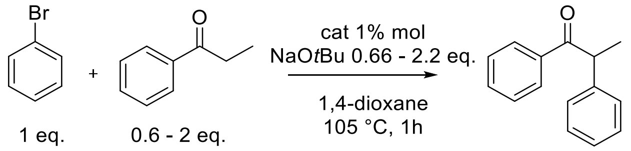

Scheme S9: General method for determining the order in propiophenone.

Complex **5d** (0.01 mmol), hexamethylbenzene (0.2 mmol) and NaO*t*Bu (1.1 mmol) were placed in a 10 mL vial and evacuated and backfilled with N_2. Dry 1,4-dioxane (2 mL), propiophenone (1 mmol) and bromobenzene (1 mmol) were added. A sample was taken (time = 0) and the reaction mixture was heated to 105 °C. After 30 seconds, the reaction time was set to zero to account for the heating of the sample. The reaction was repeated with the same conditions, keeping the amount of bromobenzene constant, but charged with different amounts of propiophenone from 0.6 mmol to 2 mmol and NaO*t*Bu respectively.

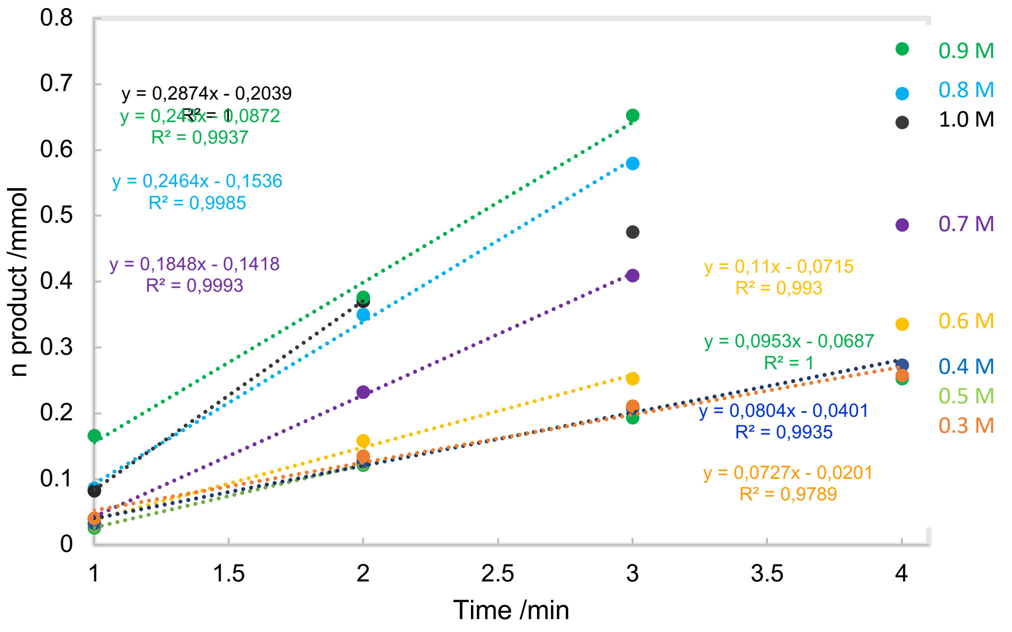

Figure S75: Time-conversion profile at different propiophenone concentrations in the linear regime, conditions see above.

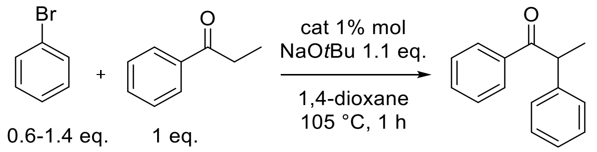

Scheme S10: General method for determining the order in bromobenzene.

Complex **5d** (0.01 mmol), hexamethylbenzene (0.2 mmol) and NaO*t*Bu (1.1 mmol) were placed in a 10 mL vial and evacuated and backfilled with N_2. Dry 1,4-dioxane (2 mL), propiophenone (1 mmol) and bromobenzene (1 mmol) were added. A sample was taken (time = 0) and the reaction mixture was heated to 105 °C. After 30 seconds, the reaction time was set to zero to account for the heating of the sample. Aliquots were taken after given times, diluted in 1,4-dioxane, filtered over celite and analyzed with GC-FID. The reaction was repeated with the same conditions but charged with different amounts of bromobenzene from 0.6 mmol to 1.4 mmol.

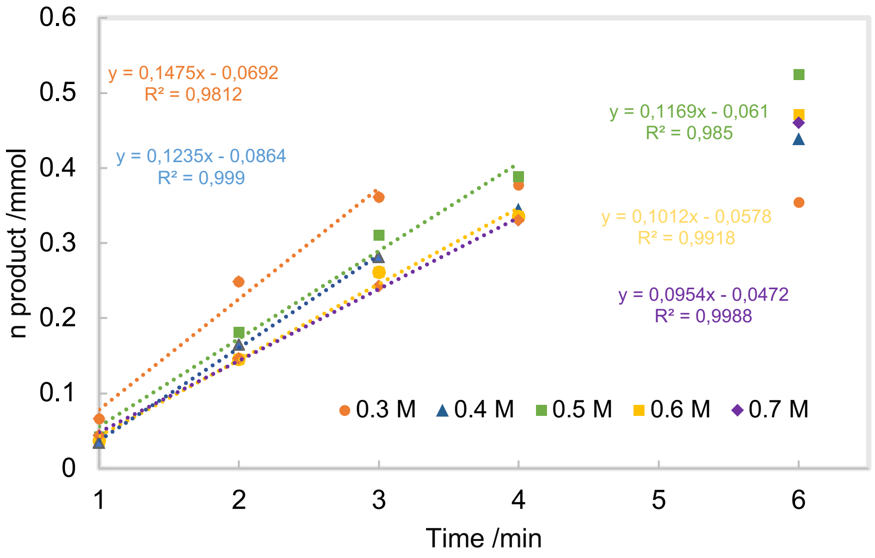

Figure S76: Time-conversion profile at different bromobenzene concentrations in the linear regime, conditions see above.

S.4.5 Procedures for catalyst poisoning experiments

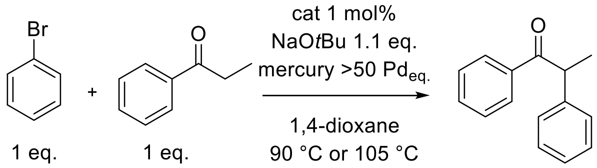

Scheme S11: Mercury poisoning test.

Complex **5a** or **5d** (0.01 mmol), hexamethylbenzene (0.2 mmol) and NaO*t*Bu (1.1 mmol) were placed in a 10 mL vial and evacuated and backfilled with N_2. Dry 1,4-dioxane (2 mL), propiophenone (1 mmol) and bromobenzene (1 mmol) were added. Time 0 sample was taken and the reaction mixture heated (105 °C for **5a**, 90 °C for **5d**). At about 30% conversion (after 12 minutes for **5a**, after 6 min for **5d**), 3 drops of elemental mercury (estimated weight 150 mg, 0,75 mmol) were added to the reaction. Aliquots were taken after given times, diluted in 1,4-dioxane, filtered over celite and analyzed with GC-FID.

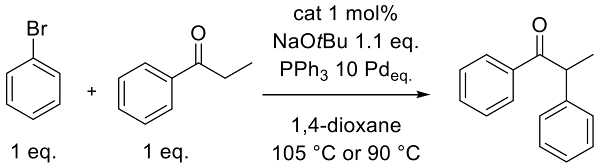

Scheme S12: General method for PPh_3 poisoning test.

Same procedure and conditions as used for the mercury poisoning experiment (see S.10.7). Instead of mercury, triphenylphosphine (26.2 mg, 100 µmol) was added.

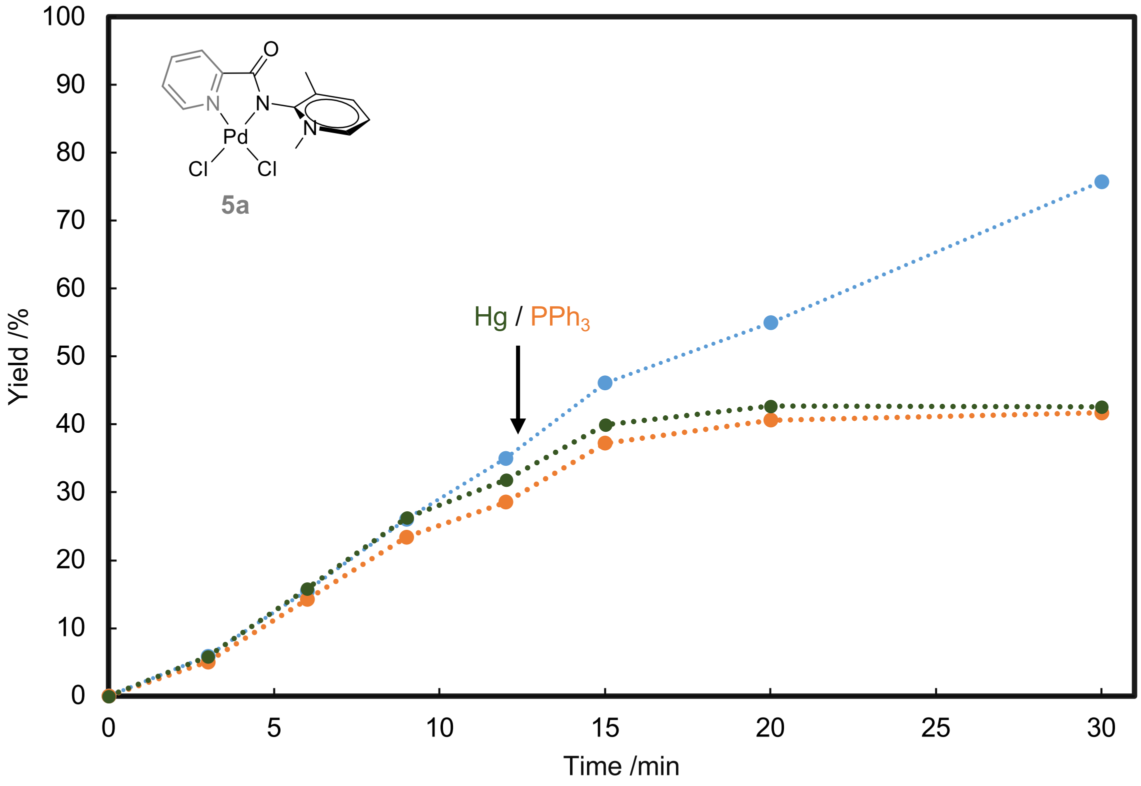

Figure S77: Conversion profile of complex **5a** including the normal run (blue), Hg poisoning (green) and PPh_3 poisoning (orange). For experimental details see sections S.10.6 and S.10.7.

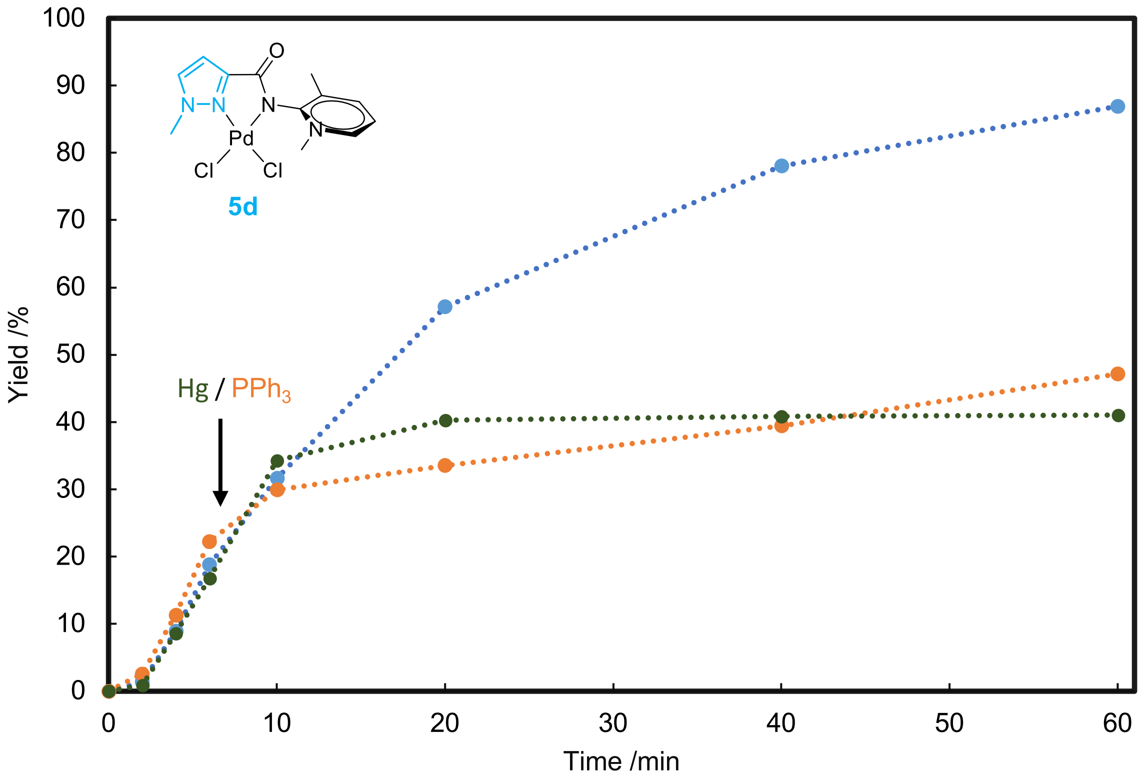

Figure S78: Catalytic profile of complex **5d** including the normal run (blue), Hg poisoning (green) and PPh_3 poisoning (orange). For experimental details see sections S.10.6 and S.10.7.

S.4.6 General procedure for temperature screening experiments

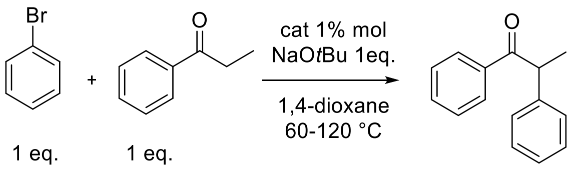

Scheme S13: General method for the temperature screening.

Complex **5d** (0.01 mmol), hexamethylbenzene (0.2 mmol) and NaO*t*Bu (1.32 mmol) were placed in a 10 mL vial. The vial was closed, evacuated and backfilled with N_2 three times. 1,4-dioxane (1mL), propiophenone (1 mmol) and bromobenzene (1 mmol) were added. A sample was taken (time = 0) and the reaction mixture was heated to 105 °C. After 30 seconds, the reaction time was set to zero to account for the heating of the sample. Aliquots were taken after given times, diluted in 1,4-dioxane, filtered over celite and analyzed with GC-FID. The reaction was repeated at different temperatures ranging from 60 °C to 120 °C using the same conditions and set-up.

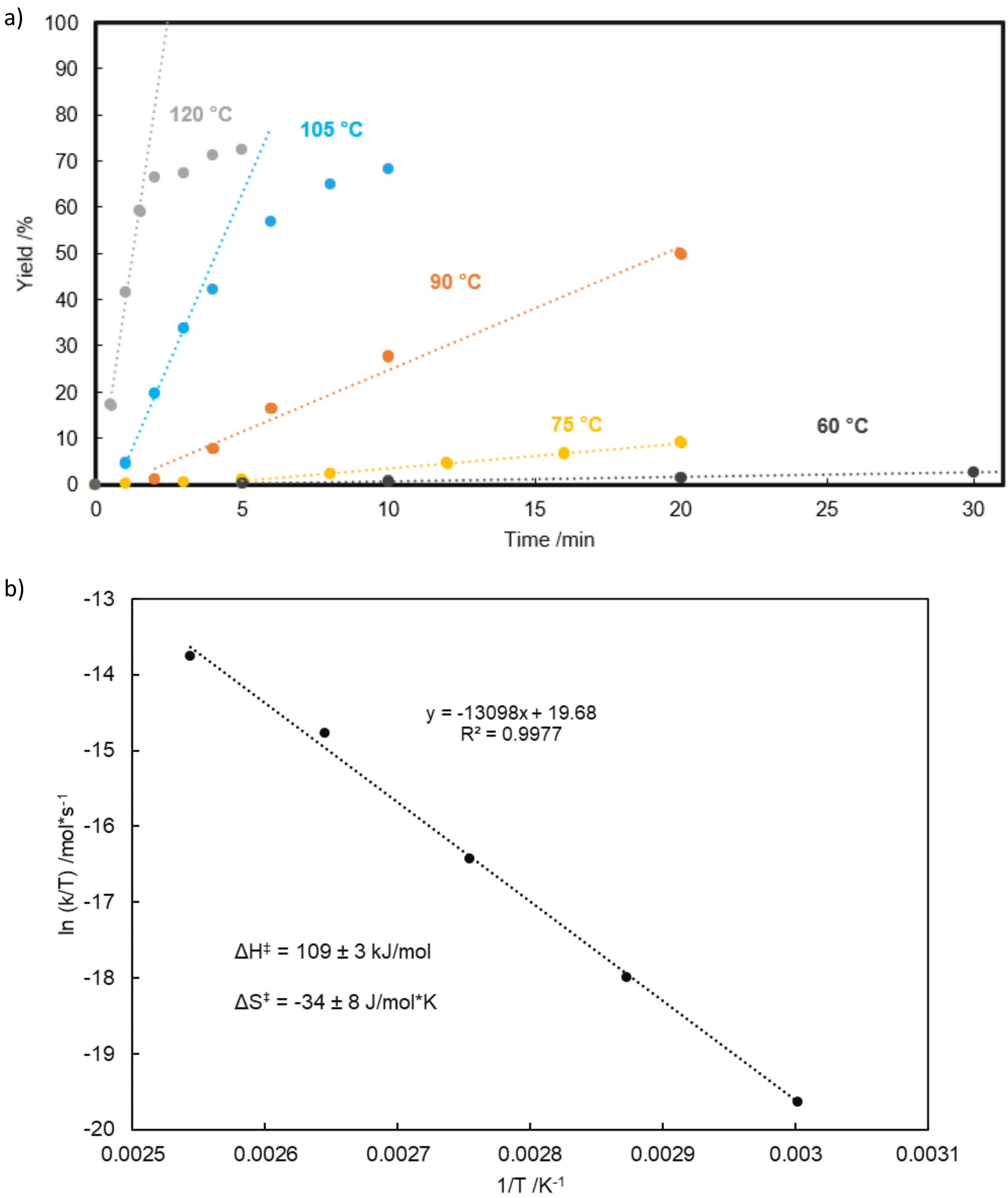

Figure S79: a) Time-conversion profile for the coupling of propiophenone and bromobenzene catalyzed by **5d** at different reaction temperatures. Reaction conditions: PhBr (1.0 mmol), Propiophenone (1.0 mmol), [Pd] (0.01 mmol), NaO*t*Bu (1.32 mmol), 1,4-dioxane (1.0 mL) in a 10 mL vial, 60-120 °C under N_2, constant sampling was performed, yield determined by GC analysis using hexamethylbenzene as internal standard. b) Eyring plot based on the temperature-dependent data.

S.4.7 Procedure for evaluating air and moisture tolerance

Same set-up and conditions used as previously described in the general α-arylation procedure (see S.10.1) were used except that for the reaction under air (complex **5d)**, the vial was closed but not evacuated and backfilled with N_2. Regarding the moisture tolerance experiment, 1,4-dioxane (99.5%) purchased from Carl Roth GmbH and kept under air was used. Additionally, Karl Fischer analysis for the dioxane kept under air revealed 0.3% H_2O content.

S.4.8 Procedure for substrates refilling experiment

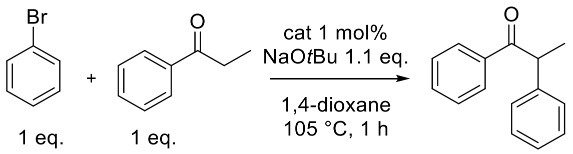

Scheme S14: General method for refilling experiment

Complex **5d** (4.12 mg, 0.01 mmol), hexamethylbenzene (32.9 mg, 0.2 mmol) and NaO*t*Bu (106.9 mg, 1.1 mmol) were placed in a 20 mL vial which was evacuated and backfilled with N_2. Dry 1,4-dioxane (2 mL), propiophenone (1 mmol) and bromobenzene (1 mmol) were added. A sample was taken (time = 0) and the reaction mixture was heated to 105 °C. After 30 seconds, the reaction time was set to zero to account for the heating of the sample. Aliquots were taken after given times, diluted in 1,4-dioxane, filtered over celite, and analyzed with GC-FID. After 30 minutes at 105 °C, 1 mL of a pre-heated stock solution of NaO*t*Bu (530.4 mg, 5.5 mmol) and bromobenzene (5 mmol) in 1,4-dioxane (5 mL) as well as propiophenone (1 mmol) were added to the reaction mixture. Samples were taken after the same reaction times and the whole refilling process was repeated four times.

S.4.9 Procedure for catalyst recycling experiments

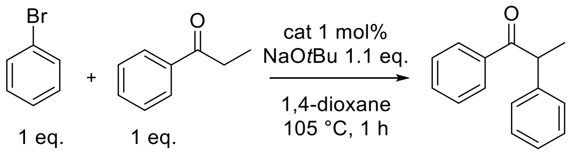

Scheme S15: General method for recycling experiment

Complex **5d** (4.09 mg, 0.01 mmol) and NaO*t*Bu (106.7 mg, 1.1 mmol) were placed in a 10 mL vial which was evacuated and backfilled with N_2. Dry 1,4-dioxane (2 mL), propiophenone (1 mmol) and bromobenzene (1 mmol) were added. The reaction mixture was heated to 105 °C for 30 minutes without sampling. Meanwhile, another vial was charged with hexamethylbenzene (33.2 mg, 0.2 mmol), NaO*t*Bu (106.3 mg, 1.1 mmol), propiophenone (1 mmol) and bromobenzene (1 mmol). After 30 minutes, the reaction mixture was cooled to room temperature and filtered under N_2 or under air into the new vial

trough a 0.2 µm syringe filter. A sample was taken (time = 0) and the reaction mixture was heated to 105 °C. After 30 seconds, the reaction time was set to zero to account for the heating of the sample. Aliquots were taken after given times, diluted in 1,4-dioxane, filtered over celite, and analyzed with GC-FID. The experiment was repeated using the same conditions, but the filtration was performed under air (Fig. S80).

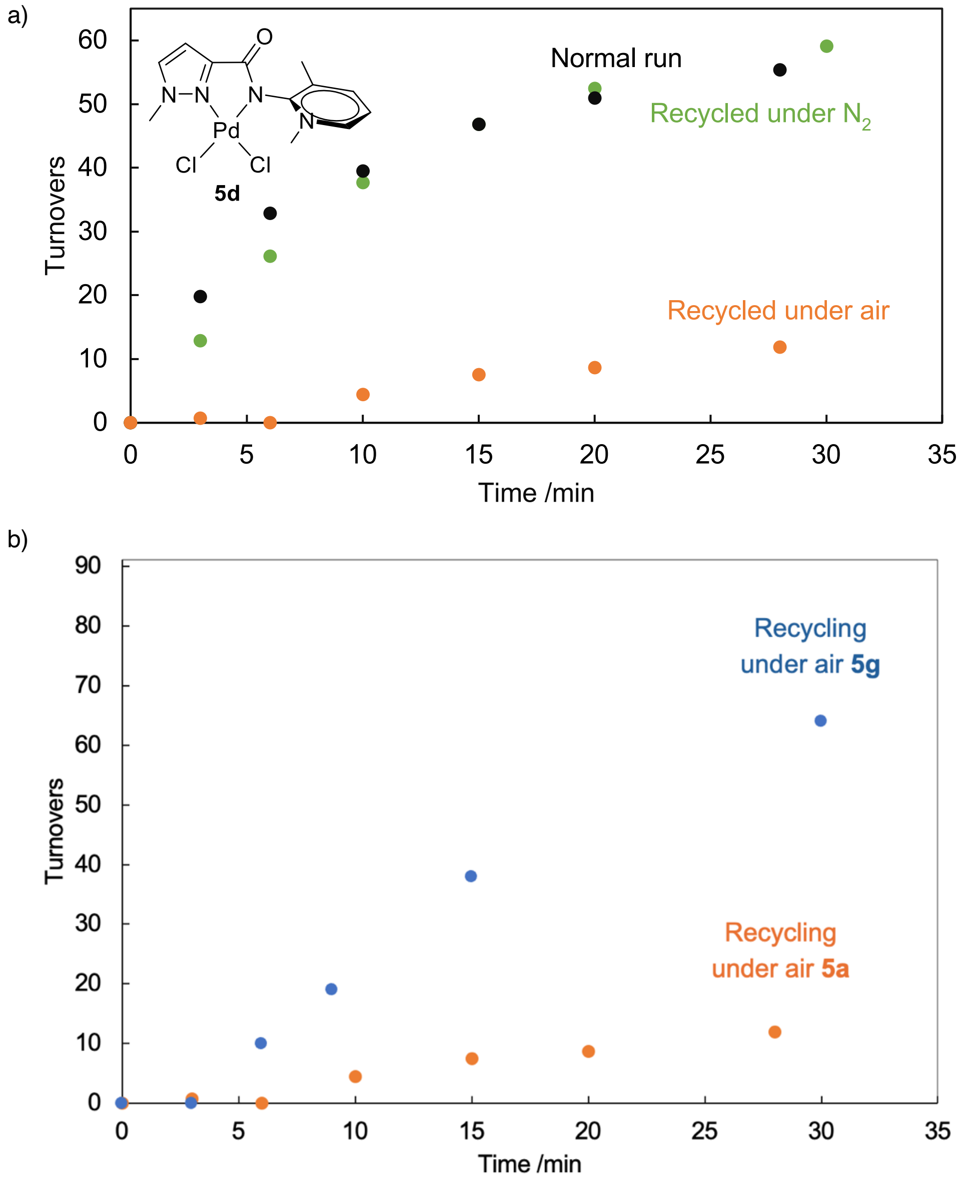

Figure S80: a) Catalyst recycling experiment for complex **5d** (black: first run; green: second run after filtration under inert atmosphere; orange: second run after filtration under air); b) recycling experiments comparing second runs after filtration in air for 5d and 5g, indicating higher robustness of 5g.

S.4.10 Studies on catalyst activation using 5d

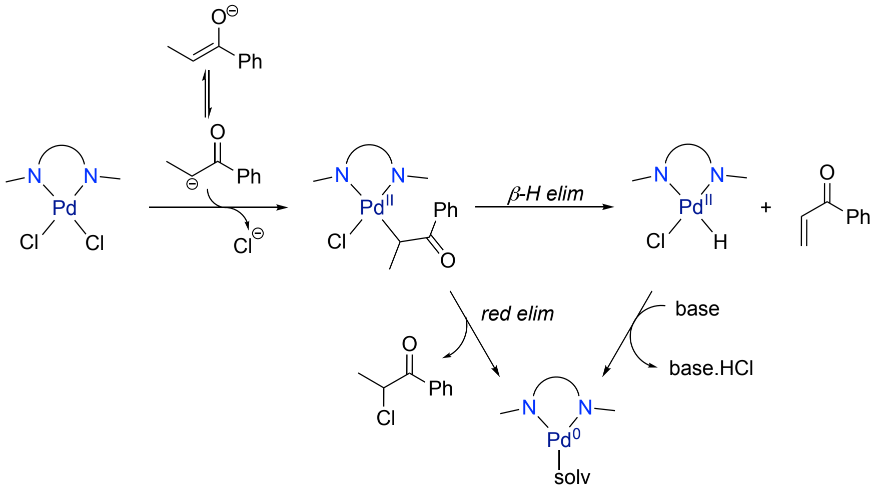

Figure S81: Potential pathways for Pd^0 generation from complexes **5** via reductive C–Cl bond elimination or via β hydrogen elimination followed by reductive HCl elimination.

Procedure for the reactivity study between 5d and NaO*t*Bu: A J-Young NMR tube charged with complex **5d** (3.1 mg, 7.6 µmol) and NaO*t*Bu (1.1 mg, 11.4 µmol) was evacuated and backfilled with N_2 three times. The solids were suspended in pre-dried and degassed C_6D_6 (0.5 mL), sonicated and analyzed by ^{1}H-NMR spectroscopy. The reaction mixture heated gradually always for 5 minutes at 30 °C, 40 °C, 50 °C, 60 °C, 70 °C, 80 °C and 90 °C and followed by ^{1}H-NMR spectroscopy.

Procedure for the reactivity study between 5d and 4-fluoropropiophenone: A stock solution of durene (20.4 mg, 152 µmol) and 4-fluoropropiophenone (10 µL, 80 µmol) was prepared in pre-dried and degassed C_6D_6 (5 mL). A J-Young NMR tube was charged with complex **5d** (1.97 mg, 4.8 µmol), evacuated and backfilled with N_2 three times. The prepared stock solution (0.2 mL; 3.2 µmol 4'-fluoropropiophenone, 6.1 µmol durene) and pre-dried and degassed C_6D (0.3 mL) were added and the reaction mixture was briefly sonicated. ^{1}H and ^{19}F{^{1}H}-NMR spectrum were recorded after 10 minutes at room temperature then the reaction mixture was gradually heated to 100°C to evaluate the thermal stability.

Procedure for the reactivity study between 5d, 4-fluoropropiophenone and NaO*t*Bu: A J-Young NMR tube was charged with complex **5d** (2.2 mg, 5.4 µmol) and NaO*t*Bu (4.5 mg, 46.8 µmol) and evacuated and backfilled with N_2 three times. The previously described 4-fluoropropiophenone stock solution (0.2 mL -> 3.2 µmol 4'-fluoropropiophenone, 6.1 µmol durene) and pre-dried and degassed benzene-d6 (0.3 mL) were added to the tube. The initially yellowish solution was analyzed by ^{1}H and ^{19}F{^{1}H}-NMR spectroscopy after 10 minutes, 60 minutes, 120 minutes and 24 hours at 23°C. After 10 minutes a colour change from yellowish to orange was observed together with the formation of an orange precipitate. Over time the reaction mixture slowly turned black.

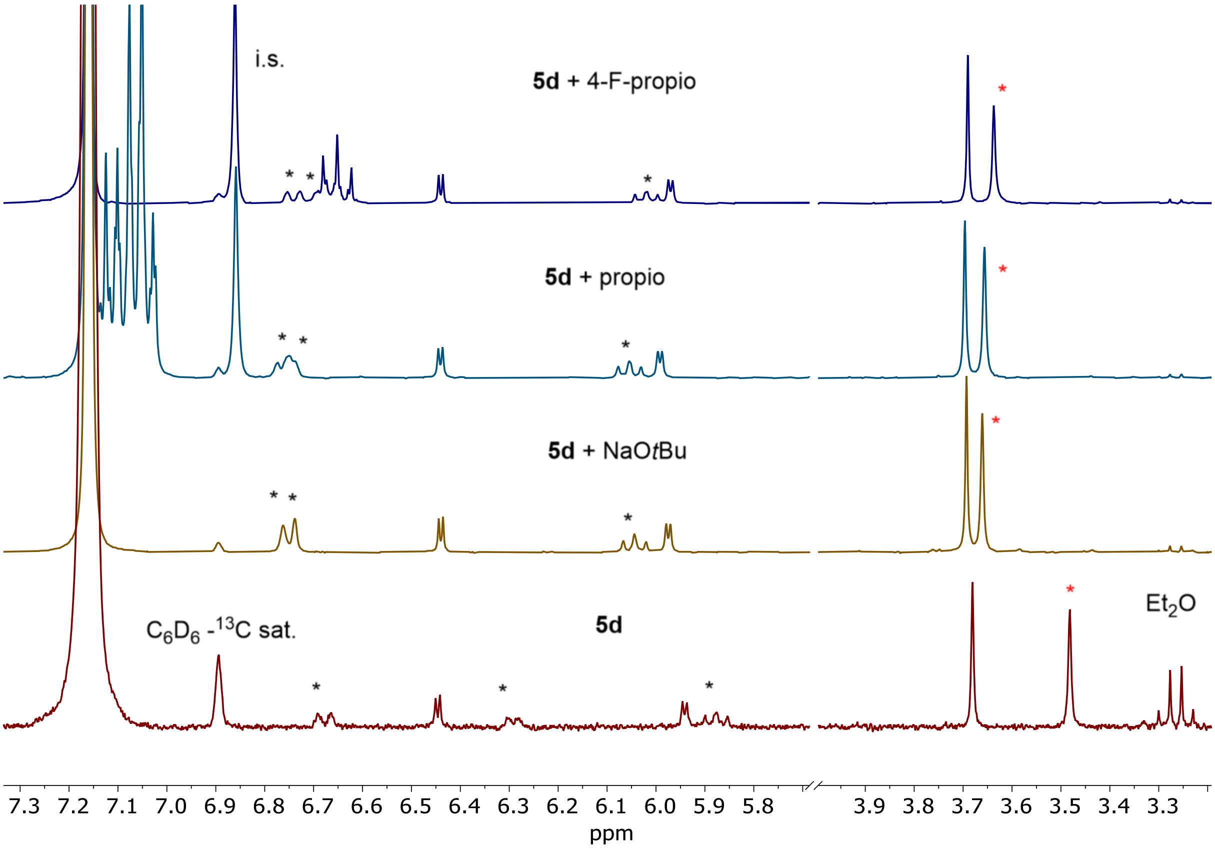

Figure S82: Stacked ^{1}H NMR spectra (C_6D_6, 298 K, 300 MHz) of from bottom to top: saturated suspension of **5d**, a mixture of **5d** (7.6 µmol) and NaO*t*Bu (11.4µmol), a mixture of **5d** (6.8 µmol), durene (380 µmol) and propiophenone (1.6 mmol), a mixture of **5d** (4.8 µmol), durene (152 µmol) and 4-fluoro-propiophenone (80 µmol). Black stars are used to highlight the aromatic proton resonances of the PYA ligand, red stars are used to highlight the N-CH_3 resonance of the PYA ligand. The signal of the residual Et_2O from the synthesis was used as an indicator for the drastic solubility increase of the complex in the presence of either a base or a ketone.

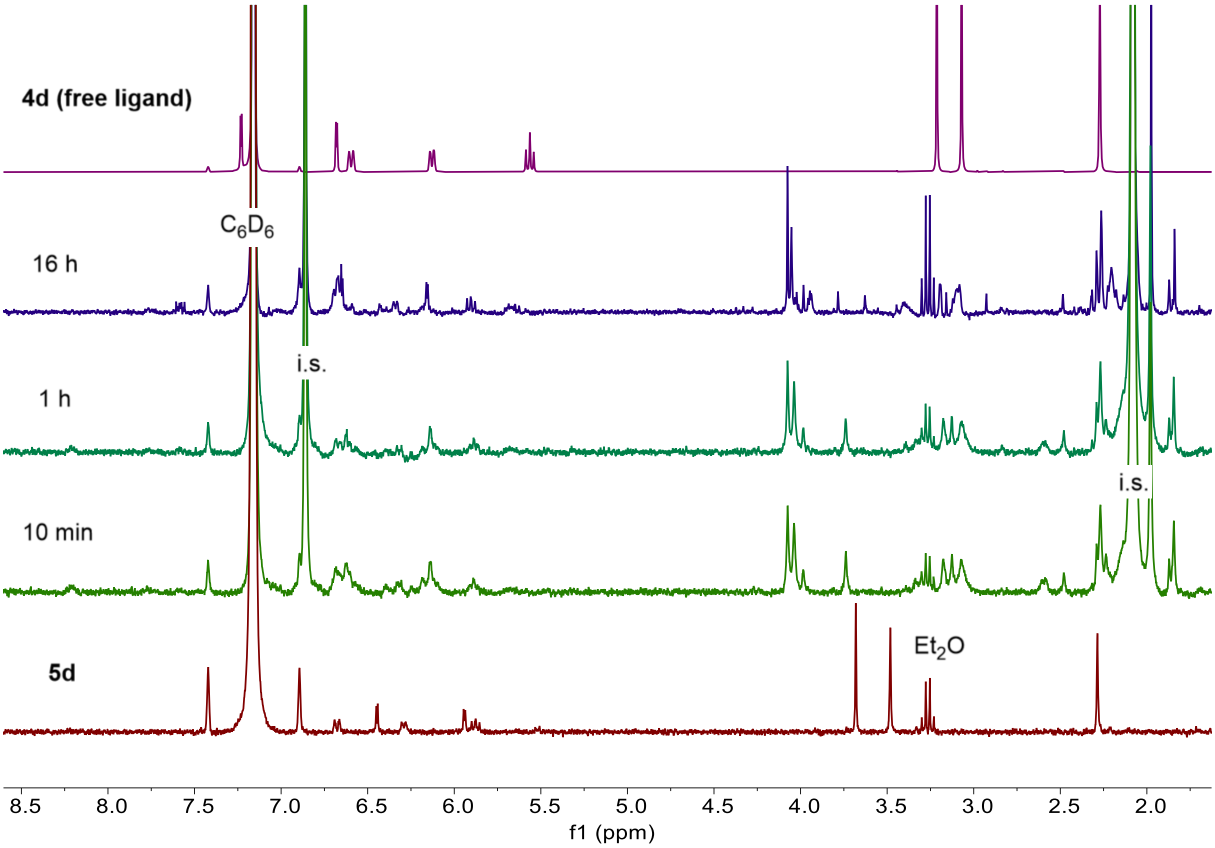

Figure S83: Stacked ^{1}H NMR spectra (C_6D_6, 298 K, 300 MHz) of from bottom to top: saturated suspension of **5d**, a mixture of **5d** (5.4 µmol) + NaO*t*Bu (47 µmol) + durene 6.1 µmol and 4-fluoropropiophenone 3.2 µmol after 10 min at 23°C, the same reaction mixture after 1h at 23 °C, the same reaction mixture after 16 h at 23 °C, the free ligand **4d** for comparison. Ketone conversion was monitored via comparison with the internal standard and revealed > 90% conv. No more accurate assessment can be made due to the poor shimming.

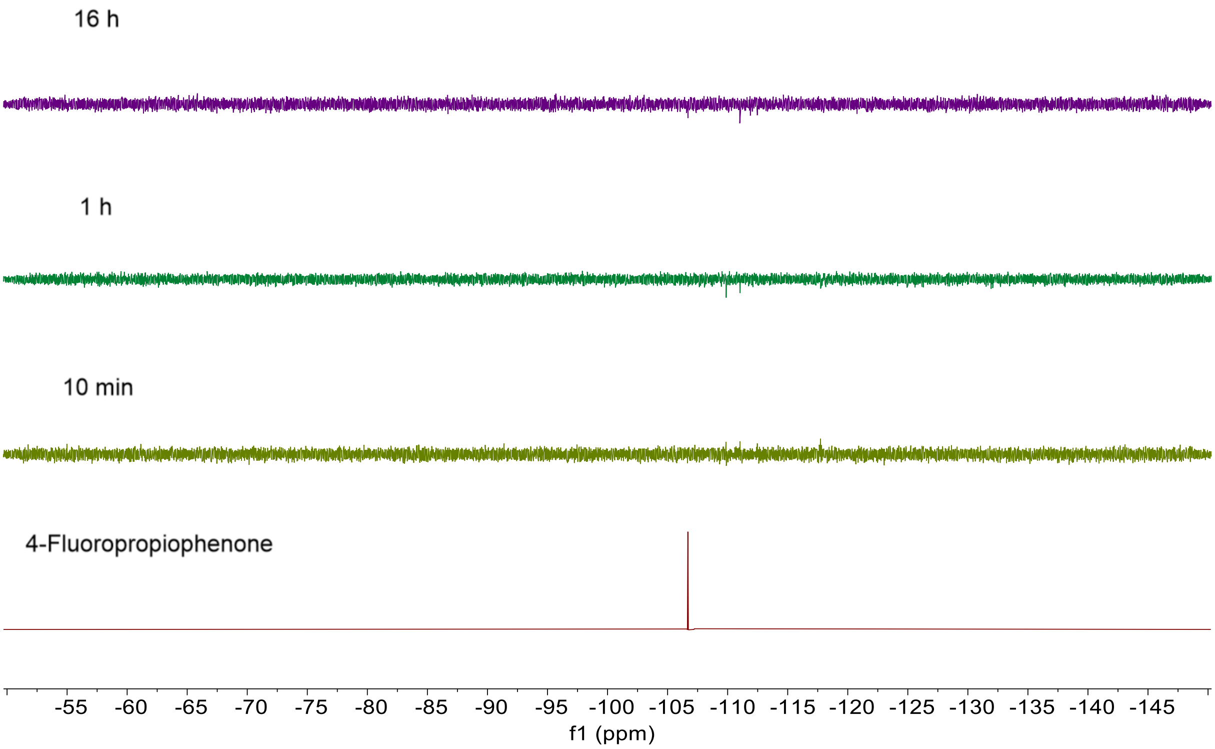

Figure S84: Stacked ^{19}F NMR spectra (C_6D_6, 298 K, 282 MHz) of from bottom to top: the 4-fluoropropiophenone stock solution, a mixture of **5d** (5.4 µmol) + NaOtBu (47 µmol) + durene (6.1 µmol) and 4-fluoropropiophenone (3.2 µmol) after 10 min at 23 °C, the same reaction mixture after 1 h at 23 °C, the same reaction mixture after 16 h at 23 °C.

S.4.11 General procedure for aryl chloride activation and competitive C–Br vs C–Cl conversion

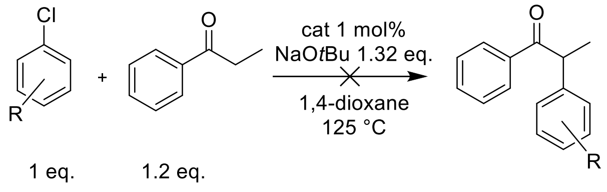

Scheme S16: General method for reaction of *o*-trifluoro-chlorobenzene using catalyst **5g**.

Complex **5g** (4.18 mg, 10 µmol), hexamethylbenzene (32.1 mg, 0.2 mmol) and NaO*t*Bu (126.5 mg, 1.32 mmol) were placed in a 10 mL vial and evacuated and backfilled with N_2. Dry 1,4-dioxane (2 mL), propiophenone (1.2 mmol) and the appropriate aryl chloride (1 mmol) were added. A sample was taken (time = 0) and the reaction mixture was heated to 125 °C. After 30 seconds, the reaction time was set to zero to account for the heating of the sample. Samples were taken after 30 minutes and 60 minutes and analyzed with ^{1}H-NMR spectroscopy in $CDCl_3$. No product was detected after 60 minutes at 125 °C.

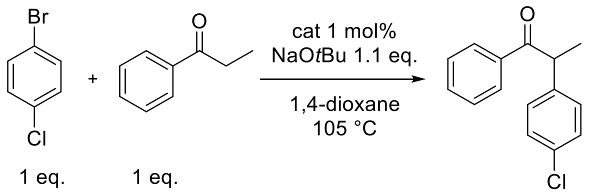

Scheme S17: General method for the competitive C-Cl vs C-Br activation using catalyst **5g**.

Complex **5g** (4.32 mg, 10 µmol), hexamethylbenzene (30.8 mg, 0.2 mmol), 1-bromo-4-chlorobenzene (190.8 mg, 1 mmol) and NaO*t*Bu (106.4 mg, 1.1 mmol) were placed in a 10 mL vial and evacuated and backfilled with N_2. Dry 1,4-dioxane (2 mL) and propiophenone (1 mmol) were added. A sample was taken (time = 0) and the reaction mixture was heated to 105 °C. After 30 seconds, the reaction time was set to zero to account for the heating of the sample. Samples were taken after 1, 2 and 20 hours and analyzed by ^{1}H NMR spectroscopy in $CDCl_3$. After 20 hours at 105 °C, the reaction mixture was cooled to room temperature and extracted with EtOAc (3 mL), organic layer was filtered over celite and analyzed by GC-MS. ^{1}H-NMR spectra in $CDCl_3$ in agreement with literature.[S14] Due to incomplete conversion of the starting material, only the aliphatic protons of the product were integrated.

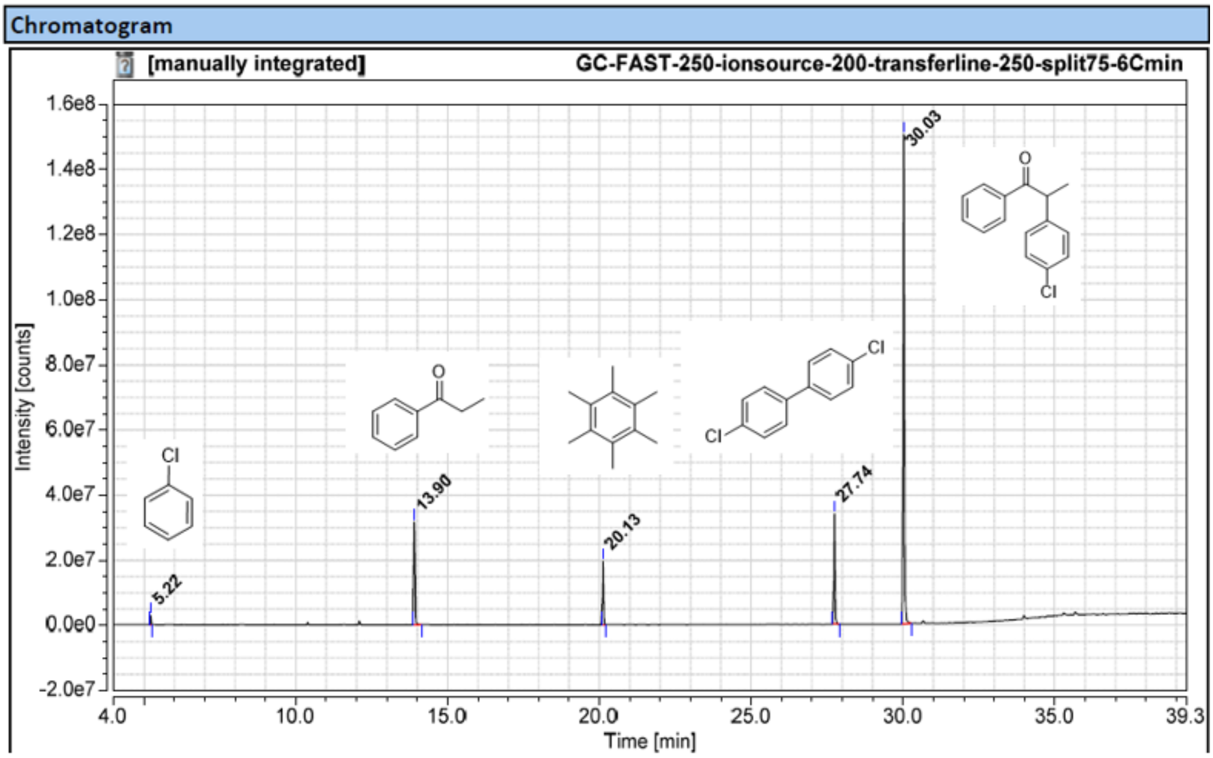

Figure S85: GC-MS chromatogram for the reaction of 1-bromo-4-chlorobenzene and propiophenone catalyzed by complex **5g** showing the absence of the brominated product.

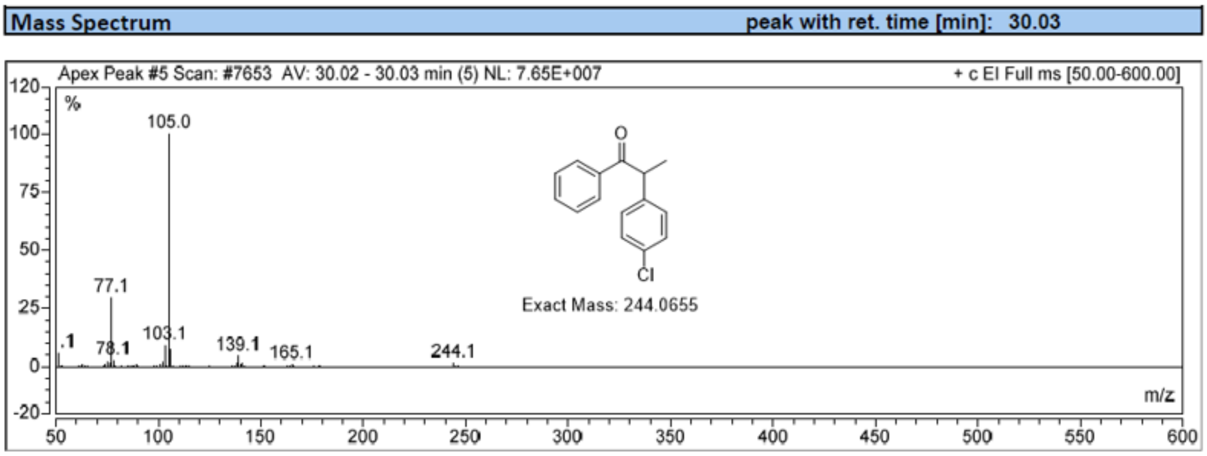

Figure S86: MS trace for compound with retention time 30.03 min for the reaction of 1-bromo-4-chlorobenzene and propiophenone catalyzed by complex **5g**.

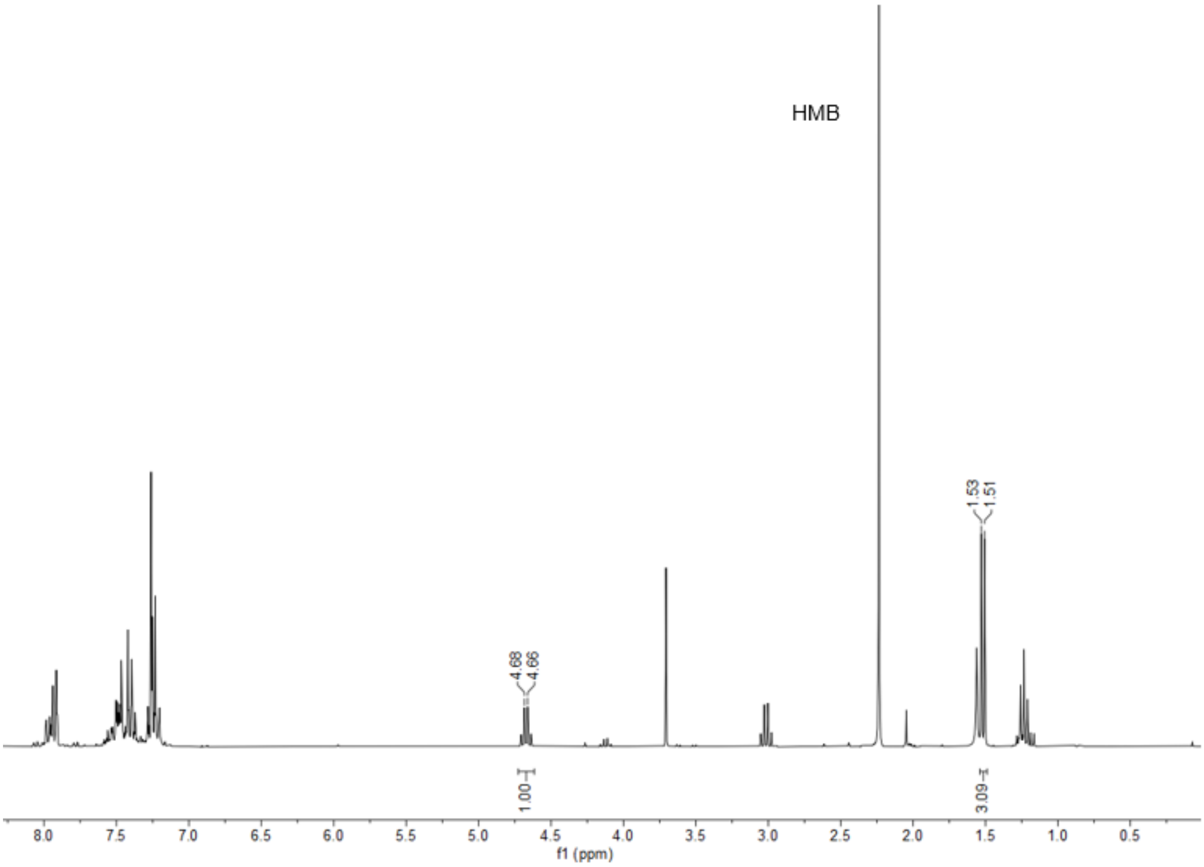

Figure S87: ^{1}H-NMR spectrum ($CDCl_3$, 298 K, 300 MHz) for the reaction of 1-bromo-4-chlorobenzene and propiophenone catalyzed by complex **5g**.

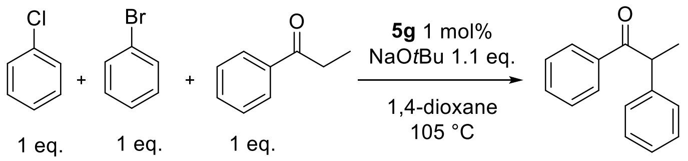

Scheme S18: Competitive propiophenone arylation in the presence chlorobenzene and bromobenzene using catalyst **5g**.

Complex **5g** (4.28 mg, 10 µmol), hexamethylbenzene (33.1 mg, 0.2 mmol) and NaO*t*Bu (105.8 mg, 1.1 mmol) were placed in a 10 mL vial and evacuated and backfilled with N_2. Dry 1,4-dioxane (2 mL), bromobenzene (1 mmol), chlorobenzene (1 mmol) and propiophenone (1 mmol) were added. A sample was taken (time = 0) and the reaction mixture was heated to 105 °C. After 30 seconds, the reaction time was set to zero to account for the heating of the sample. Samples were taken after 1, 2 and 20 h, diluted in 1,4-dioxane, filtered over celite and analyzed by GC-FID.

S.5 References

S1 E. Reusser and M. Albrecht, *Dalton Trans.*, 2023, **52**, 16688–16697.

S2 P. Sambasiva Rao, C. Kurumurthy, B. Veeraswamy, G. Santhosh Kumar, Y. Poornachandra, C. Ganesh Kumar, S. B. Vasamsetti, S. Kotamraju and B. Narsaiah, *Eur. J. Med. Chem.*, 2014, **80**, 184–191.

S3 W. A. A. Arafa and A. E.-A. A. Nayl, *Appl. Organomet. Chem.*, 2019, **33**, e5156.

S4 Lifechemicals Shop, https://shop.lifechemicals.com/compound/1/F2146-0680, (accessed 19 December 2023).

S5 A. Kolarovič, M. Schnürch and M. D. Mihovilovic, *J. Org. Chem.*, 2011, **76**, 2613–2618.

S6 KYN THERAPEUTICS, US Pat., WO2019036657A1, 2019.

S7 G. M. Ó. Máille, A. Dall'Anese, P. Grossenbacher, T. Montini, B. Milani and M. Albrecht, *Dalton Trans.*, 2021, **50**, 6133–6145.

S8 Oxford Diffraction (2018). CrysAlisPro (Version 1.171.40.37a). Oxford Diffraction Ltd., Yarnton, Oxfordshire, UK.

S9 G. M. Sheldrick, *Acta Cryst.,* 2015*,* **A71***,* 3-8.

S10 G. M. Sheldrick, *Acta Cryst.,* 2015*,* **C71***,* 3-8.

S11 O. V. Dolomanov, L. J. Bourhis, R. J. Gildea, J. a. K. Howard and H. Puschmann, *J. Appl. Crystallogr.*, 2009, **42**, 339–341.

S12 L. Falivene, Z. Cao, A. Petta, L. Serra, A. Poater, R. Oliva, V. Scarano and L. Cavallo, *Nat. Chem.*, 2019, **11**, 872–879.

S13 J. Burés, *Angew. Chem. Int. Ed.*, 2016, **55**, 2028–2031.

S14 S.-R. Wang and P.-Q. Huang, *Chin. J. Chem.*, 2019, **37**, 887–891.
